# Chameleonic Photo- and Mechanoluminescence in Pyrazolate-Bridged NHC Cyclometalated Platinum Complexes

**DOI:** 10.1021/acs.inorgchem.1c01470

**Published:** 2021-08-02

**Authors:** Violeta Sicilia, Lorenzo Arnal, Daniel Escudero, Sara Fuertes, Antonio Martin

**Affiliations:** † Departamento de Quimica Inorganica, Escuela de Ingenieria y Arquitectura de Zaragoza, Instituto de Sintesis Quimica y Catalisis Homogenea (ISQCH), CSIC - Universidad de Zaragoza, Campus Rio Ebro, Edificio Torres Quevedo, 50018, Zaragoza, Spain; ‡ Departamento de Quimica Inorganica, Facultad de Ciencias, Instituto de Sintesis Quimica y Catalisis Homogenea (ISQCH), CSIC - Universidad de Zaragoza, Pedro Cerbuna 12, 50009, Zaragoza, Spain; § Department of Chemistry, KU Leuven, Celestijnenlaan 200f - box 2404, 3001 Leuven, Belgium

## Abstract

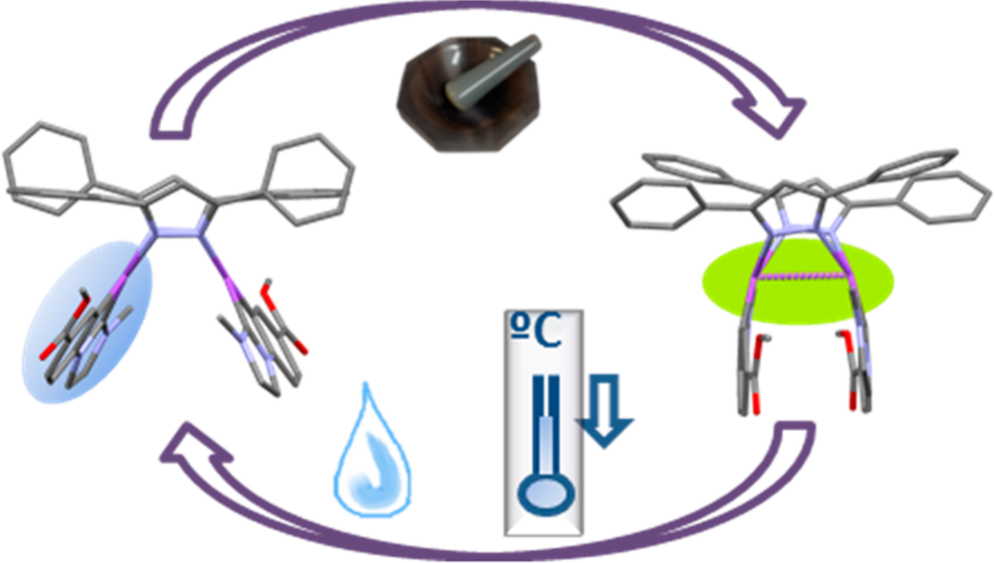

DFT investigations on the ground (GS) and the first triplet (T_1_) excited state potential energy surfaces (PES) were performed on a new series of platinum-butterfly complexes, [{Pt(C^∧^C*)(μ-Rpz)}_2_] (Rpz: pz, **1**; 4-Mepz, **2**; 3,5-dmpz, **3**; 3,5-dppz, **4**), containing a cyclometalated NHC in their wings. The geometries of two close-lying local minima corresponding to butterfly spread conformers, **1s**–**4s**, and butterfly folded ones, **1f**–**4f**, with long and short Pt–Pt separations, respectively, were optimized in the GS and T_1_ PES. A comparison of the GS and T_1_ energy profiles revealed that an opposite trend is obtained in the relative stability of folded and spread conformers, the latter being more stabilized in their GS. Small Δ*G* (**s**/**f**) along with small-energy barriers in the GS support the coexistence of both kinds of conformers, which influence the photo- and mechanoluminescence of these complexes. In 5 wt % doped PMMA films in the air, these complexes exhibit intense sky-blue emissions (PLQY: 72.0–85.9%) upon excitation at λ ≤ 380 nm arising from ^3^IL/MLCT excited states, corresponding to the predominant **1s**–**4s** conformers. Upon excitation at longer wavelengths (up to 450 nm), the minor **1f**–**4f** conformers afford a blue emission as well, with PLQY still significant (40%–60%). In the solid state, the as-prepared powder of **4** exhibits a greenish-blue emission with QY ∼ 29%, mainly due to ^3^IL/^3^MLCT excited states of butterfly spread molecules, **4s**. Mechanical grinding resulted in an enhanced and yellowish-green emission (QY ∼ 51%) due to the ^3^MMLCT excited states of butterfly folded molecules, **4f**, in such a way that the mechanoluminescence has been associated with an intramolecular structural change induced by mechanical grinding.

## Abstract 2

The intriguing luminescence of a new series of platinum-butterfly molecules, [{Pt(C^∧^C*)(μ-Rpz)}_2_], have been studied and deciphered with DFT and TD-DFT calculations. The low energy barrier for the thermal interconversion between the spread (**4s**) and folded (**4f**) structures in the GS of the 3,5-diphenylpyrazolate compound, **4**, seems to be the basis of the stimuli-responsive luminescence of complex **4**.

## Introduction

Cyclometalated complexes of Pt(II) are characterized by outstanding photoluminescent properties,^1^ arising from the radiative deactivation of triplet excited states, which are at the origin of many challenging applications such as optical sensors,^2^ biological imaging,^3^ or light emitting devices.^4^ The planar geometry of the mononuclear complexes allows them to assemble through Pt···Pt and π–π interactions. Recent computational investigations demonstrate that mononuclear complexes often possess very different photophysical properties than their aggregates. Thus, while mononuclear complexes are likely to deactivate nonradiatively through triplet metal-centered (^3^MC) excited states,^5^ the formation of the latter states is likely more hindered in condensed phases. Additionally, it was found that the formation of excimers in Pt(II) complexes is more favored than the formation of ground state aggregates.^6^ As a result, the nature of the emissive triplet state changes from a monomer-based triplet emission to a triplet metal–metal-to-ligand charge transfer (^3^MMLCT) like emission in the molecular ensembles, leading to red-shifted emissions.^7,8^ This kind of platinum compound very often suffers the so-called aggregation-cause quenching (ACQ)^9^ effect, which limits their applications. However, many transition metal complexes exhibit aggregation-induced phosphorescent emission (AIPE),^10−14^ and they can successfully be used to achieve white light^15,16^ or NIR^17,18^ organic-light-emitting diodes by adjusting the doping concentration. The emission color strongly depends on the extent of these intermolecular interactions, and in their turn, on environmental factors able to affect them, such as temperature variations,^8^ mechanical force,^12,19^ or volatile solvent molecules embedded into the lattice.^2,20^ Thus, these compounds become thermo-, mechano-, and/or vapoluminescent complexes, enlarging the technological interest of these smart functional materials.

Compared to mononuclear complexes, binuclear luminescent complexes have been less explored.^21−28^ In binuclear Pt(II) cyclometalated compounds, the metallophilic interactions and thereto their luminescent properties can partially be controlled by selecting the bridging ligands, which result in different degrees of rigidity and steric hindrance.^21,23,24^ Among them, platinum complexes with bridging pyrazolates have been deeply studied by Castellano,^25^ Thompson,^26,27^ and Ma^28^ and co-workers. It was found that the Pt–Pt distance and the extent of the metallophilic interactions can be tuned by the bulkiness of the pyrazolate unit (butterfly body), in such a way that when the bulkiness increases, the cycloplatinated units (butterfly wings) are pushed closer together. As a result, the emission color of this kind of complexes can be tuned from blue to green or red.^23^ In addition, in solution they exhibit sometimes a photoinduced structural change (PSC) on the lowest triplet-state potential energy surface (PES), resulting in a dramatic change of the Pt–Pt bond distance and thereto on the emission.^28^ This unique butterfly-like structure allows the contraction of the Pt–Pt distance with temperature, thus leading to solid-state thermochromism and thermoluminescence. This is the case of [{Pt(ppy)(μ-Ph_2_pz)}_2_],^29^ which at a low temperature exhibits monomer-based ^3^LC/MLCT emission, and it changes to excimer-like ^3^MMLCT emission above 160 K.

The cyclometalating groups play also an important role in the stability and the control of the photophysical properties.^30^ In this sense, the platinum-butterfly complexes reported by Strassner et al., i.e., [{Pt(C^∧^C*)(μ-Rpz)}_2_]^31,32^ are exemplary ones. They revealed that the cyclometalated N-heterocyclic carbenes (C^∧^C*), forming two strong metal–carbon bonds, are excellent wingsa for the synthesis of highly efficient blue and orange emitters. In this field, we reported compound [{Pt(C^∧^C*)(μ-pz)}_2_] (HC^∧^C* = 1-(4-(ethoxy-carbonyl)phenyl)-3-methyl-1H-imidazol-2-ylidene; pz: pyrazolate **1**) which undergoes two-center, two-electron [2c, 2e] oxidation in the presence of haloforms (CHX_3_, X = Cl, Br, I).^33^ Herein, we report three new complexes, [{Pt(C^∧^C*)(μ-Rpz)}_2_] (Rpz: 4-methylpyrazolate (4-Mepz), **2**; 3,5-dimethylpyrazolate (3,5-dmpz), **3**; and 3,5-diphenylpyrazolate (3,5-dppz), **4**) bearing the same wings, C^∧^C*, but different bodies (Rpz). Besides the experimental synthesis and characterization of compounds **1**–**4**, the intriguing luminescence and mechanoluminescence have been studied and deciphered with density functional theory (DFT) and time-dependent DFT (TD-DFT) investigations. For all of the complexes, two close-lying local minima corresponding to the folded (**f**) and spread (**s**) conformers were located on both the ground-state (GS) and the lowest adiabatic triplet excited state (T_1_) PES. A low energy barrier for the thermal interconversion between both structures in the GS seems to be at the core of the stimuli-responsive luminescence of complex **4** in the solid state.

## Experimental Section

Compounds [{Pt(EtO_2_C–C^∧^C*)(μ-Cl)}_2_] (**A**),^34^ [Pt(EtO_2_C–C^∧^C*)(4-MepzH)_2_]ClO_4_ (**B2**),^35^ and [{Pt(EtO_2_C–C^∧^C*)(μ-pz)}_2_] (**1**)^36^ were prepared as described elsewhere.

### Synthesis of *syn*-/*anti*-[{Pt(EtO_2_C–C^∧^C*)(μ-4-Mepz)}_2_] (**2**)

NEt_3_ (0.5 mL, 3.62 mmol) was added to a solution of **B2** (133.5 mg, 0.19 mmol) in acetone (30 mL) at room temperature. After 2 h of reaction, the solvent was removed in vacuo to 2 mL. The solution was treated with H_2_O (20 mL), filtered, and washed with H_2_O to give **2-***anti* (83%)/**2**-*syn*(17%) as a yellow solid. Yield: 73.7 mg, 75%. Anal. Calcd for C_34_H_36_N_8_O_4_Pt_2_: C, 40.40; H, 3.59; N, 11.08. Found: C, 40.00; H, 3.75; N, 11.06. ^1^H NMR data for **2-***anti* (500 MHz, acetone-*d*_6_): δ 8.02 (d, ^4^*J*_H7,H9_ = 1.8, ^3^*J*_H7,Pt_ = 55.1, 2H, H_7_), 7.65 (d, ^3^*J*_H2,H3_ = 2.1, 2H, H_2_), 7.63 (dd, ^3^*J*_H9,H10_ = 8.1, ^4^*J*_H9,H7_ = 1.8, 2H, H_9_), 7.50 (s, 2H, H_3′_, 4-Mepz), 7.46 (s, 2H, H_5′_, 4-Mepz), 7.18 (d, ^3^*J*_H10,H9_ = 8.1, 2H, H_10_), 7.06 (d, ^3^*J*_H3,H2_ = 2.1, 2H, H_3_), 4.23 (m, CH_2_, CO_2_Et), 3.35 (s, 6H, H_4_), 2.12 (s, 6H, Me, 4-Mepz), 1.31 (t, ^3^*J*_H,H_ = 7.1, 6H, CH_3_, CO_2_Et). ^1^H NMR data for **2-***syn*: δ 7.97 (d, ^4^*J*_H7,H9_ = 1.8, 2H, H_7_), 7.68 (d, ^3^*J*_H2,H3_ = 2.1, 2H, H_2_), 7.58 (dd, ^3^*J*_H9,H10_ = 8.1, ^4^*J*_H9,H7_ = 1.8, 2H, H_9_), 7.54 (s, 2H, H_3′_, 4-Mepz), 7.42 (s, 2H, H_5′_, 4-Mepz), 3.63 (s, 6H, H_4_), 1.35 (t, ^3^*J*_H,H_ = 7.1, 6H, CH_3_, CO_2_Et). The rest of the signals appear overlapped with those of the **2-***anti* isomer. ^13^C{^1^H} NMR plus HSQC and HMBC data for **2-***anti* (125.75 MHz, acetone-*d*_6_): δ 161.2 (C_1_), 152.9 (C_5_), 139.6 and 138.3 (C_3′_ and C_5′_), 136.3 (C_7_), 133.8 and 126.8 (C_6_ and C_8_), 126.3 (C_9_), 123.3 (C_3_), 116.2 (C_2_), 116.1 (C_4’_), 110.9 (C_10_), 60.7 (CH_2_, CO_2_Et), 36.5 (C_4_), 14.6 (CH_3_, CO_2_Et), 9.5 (Me, 4-Mepz). ^13^C{^1^H} NMR plus HSQC and HMBC data for **2-***syn* (125.75 MHz, acetone-*d*_6_): δ = 160.3 (C_1_), 123.1 (C_3_), 36.7 (C_4_). ^195^Pt{^1^H} NMR (108 MHz, acetone-*d*_6_): δ −3775 (**2-***anti*), −3785 (**2-***syn*) ppm. (MS (MALDI+): *m*/*z* 1010.4 [{Pt(C^∧^C*)(μ-4-Mepz)}_2_].

### Synthesis of *syn*-/*anti*-[{Pt(EtO_2_C–C^∧^C*)(μ-3,5-dmpz)}_2_] (**3**)

Compound **A** (106.3 mg, 0.12 mmol) was added to a solution containing NaO^*t*^Bu (22.2 mg, 0.23 mmol) and 3,5-dmpzH (22.5 mg, 0.23 mmol) in acetone/EtOH (10 mL/5 mL). After 3 h of reaction at −10 °C, the solvent was removed to 3 mL under reduced pressure, filtered, and washed with 2 × 5 mL of H_2_O to give **3-**anti(80%)/**3**-syn(20%) as a yellow solid. Yield: 70 mg, 58%. Anal. Calcd for C_36_H_40_N_8_O_4_Pt_2_: C, 41.62; H, 3.88; N, 10.79. Found: C, 41.24; H, 4.02; N, 10.76. ^1^H NMR data for **3-***anti* (500 MHz, methylene chloride- *d*_2_): δ 7.83 (d, ^4^*J*_H7,H9_ = 1.7, ^3^*J*_H7,Pt_ = 52.3, 2H, H_7_), 7.67 (d, ^3^*J*_H9,H10_ = 7.5, 2H, H_9_), 7.20 (s, br, 2H, H_2_), 6.94 (d, ^3^*J*_H10,H9_ = 7.5, 2H, H_10_), 6.67 (s, br, 2H, H_3_), 6.11 (s, 2H, H_4′_, dmpz), 4.24 (m, CH_2_, CO_2_Et), 3.33 (s, 6H, H_4_), 2.32 and 2.27 (s, 12H, Me, dmpz), 1.35 (t, ^3^*J*_H,H_ = 7.1, 6H, CH_3_, CO_2_Et). ^1^H NMR data for **3**-*syn*: δ 6.13 and 6.04 (s, 2H, H_4′_, dmpz), 3.58 (s, 6H, H_4_). The rest of the signals appear overlapped with those of the **3**-*anti*.

^13^C{^1^H} NMR plus HSQC and HMBC data for **3**-*anti* (125.75 MHz, methylene chloride-*d*_2_): δ 160.7 (C_1_), 152.2 (C_5_), 146.7 (C_3′_ and C_5′_). 136.9 (C_7_), 133.4 and 126.7 (C_6_ and C_8_), 125.9 (C_9_), 122.1 (C_3_), 115.4 (C_2_), 110.2 (C_10_), 104.6 (C_4’_), 60.8 (CH_2_, CO_2_Et), 35.7 (C_4_), 14.7 (CH_3_, CO_2_Et), 14.2 (Me, dmpz). ^13^C{^1^H} NMR plus HSQC and HMBC data for **3-***syn* (125.75 MHz, methylene chloride-*d*_2_): 160.2 (C_1_), 35.8 (C_4_). ^195^Pt{^1^H} NMR (108 MHz, methylene chloride- *d*_2_): δ = −3771 ppm (**3**-*anti*), −3799 (**3**-*syn*) ppm. MS (MALDI+): *m*/*z* 1038.2 [{Pt(C^∧^C*)(μ-dmpz)}_2_].

### Synthesis of *syn-/anti*- [{Pt(EtO_2_C–C^∧^C*)(μ-3,5-dppz)}_2_] (4)

AgClO_4_ (52.7 mg, 0.25 mmol) was added to a stirred suspension of **A** (115.8 mg, 0.12 mmol) in acetone (30 mL) in the dark at room temperature. After 2 h of reaction, 3,5-dppzH (110.9 mg, 0.50 mmol) was added to the mixture and allowed to react overnight in the darkness. Then, the resulting suspension was filtered through Celite and concentrated to ca. 20 mL. NEt_3_ (0.5 mL, 3.62 mmol) was added to the reaction mixture and stirred for 2 h. Then, the solvent was removed in vacuo. The residue was treated with cold MeOH (5 mL) and filtered to give **4**-*anti* (94%)/**4**-*syn* (6%) as a yellow solid. Yield: 90.0 mg, 74%. Anal. Calcd for C_56_H_48_N_8_O_4_Pt_2_: C, 52.25; H, 3.76; N, 8.71. Found: C, 52.64; H, 3.90; N, 8.82. ^1^H NMR data for **4-***anti* (500 MHz, DMSO-*d*_6_, 353 K): δ 8.59 (d, ^3^*J*_H*o*,H*m*_ = 7.2, 4H, H_*o*_), 8.25 (dd, ^3^*J*_H*o*,H*m*_ = 7.2, ^4^*J*_H*o*,H*p*_ = 1.8, 4H, H_*o*_), 7.84 (s, br, 1H, H_C^∧^C*_), 7.72–7.53 (m, 4H, H_C^∧^C*_), 7.50–7.24 (m, 9H, H_pz_ and H_C^∧^C*_), 7.20–7.03 (m, 8H, H_pz_ and H_C^∧^C*_), 6.99 (s, br, 2H, H_pz_), 4.18 (q, ^3^*J*_H,H_ = 7.0, 4H, CH_2_, CO_2_Et), 3.18 (s, 6H, H_4_), 1.29 (t, ^3^*J*_H,H_ = 7.0, 6H, CH_3_, CO_2_Et). ^1^H NMR data for **4-***syn*: δ 3.32 (s, 6H, H_4_). The rest of the signals appear overlapped with those of the **4**-*anti* isomer. ^13^C{^1^H} NMR plus HSQC and HMBC data for **4**-*anti* (125.75 MHz, DMSO-*d*_2_,353 K): δ 155.5 (C_1_), 135.1 (C_C^∧^C*_), 132.7 (C_pz_), 132.6 (C_pz_), 127.6 (C_pz_), 127.0 (C_pz_), 126.8 (C_C^∧^C*_), 126.7 (C_*o*_), 125.4 (C_*o*_), 124.7 (C_C^∧^C*_), 124.5 (C_C^∧^C*_), 122.1 (C_C^∧^C*_), 109.7 (C_C^∧^C*_), 103.2 (C_C^∧^C*_), 59.3 (CH_2_, CO_2_Et), 34.3 (C_4_), 13.6 (CH_3_, CO_2_Et). ^195^Pt{^1^H} NMR (108 MHz, DMSO-*d*_6_, 353 K): δ −3680 ppm (**4**-*anti*). (MS (MALDI+): *m*/*z* 1286.5 [{Pt(C^∧^C*)(μ-3,5-dppz)}_2_].

## Results and Discussion

**Scheme 1:**
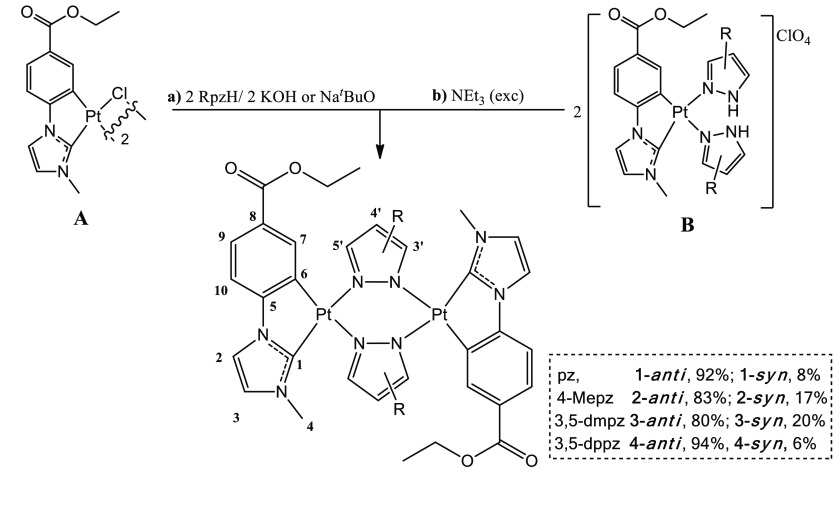
Synthetic Routes Followed for Compounds **1**–**4** –2 KCl (NaCl)/–2 H_2_O (^*t*^BuOH). –2 NHEt_3_Rpz/–2 NHEt_3_ClO_4_. Just the major isomer “*anti*” appears, represented for clarity along with its numerical scheme for NMR analysis. Compound **1** is included for overview.

Compounds [{Pt(C^∧^C*)(μ-Rpz)}_2_] (HC^∧^C* = 1-(4-(ethoxycarbonyl) phenyl)-3-methyl-1*H*-imidazol-2-ylidene; Rpz: 4-methylpyrazolate (4-Mepz), **2**; 3,5-dimethylpyrazolate (3,5-dmpz), **3**; 3,5-diphenylpyrazolate (3,5-dppz), **4**) were prepared following path a (for **3**) or b (for **2** and **4**) in Scheme 1).

**Figure 1:**
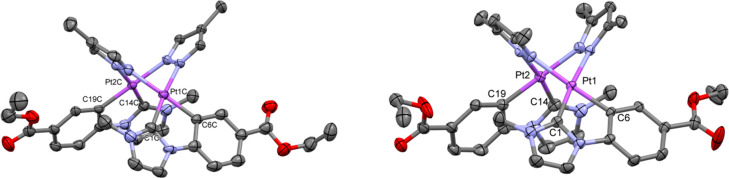
Molecular structures of **2A** (left) and **3** (right). Ellipsoids are drawn at their 50% probability level; solvent molecules and hydrogen atoms have been omitted for clarity.

The inability to get compound **3** through path b is in agreement with the greater basicity of 3,5-dmpzH^37^ with respect to pzH, 4-MepzH, and 3,5-dppzH, which prevents it from being removed from the coordination sphere of the platinum center (experimental details for **2**–**4** in the SI). All the complexes were obtained as a mixture of *syn*/*anti* isomers with respect to the relative orientation of the cyclometalated C^∧^C* groups, with the anti-isomer being the predominant one, as can be seen in the ^1^H and the ^195^Pt{^1^H} NMR spectra of **2**–**4** (Figures S1–S4). The single-crystal X-ray diffraction study of **2** and **3** confirmed the expected spread butterfly-like structure (Figure 1). Like compound **1**,^36^ complex **2** showed three different molecules in the asymmetric unit (A, B, C) with intermetallic distances of 3.355(4) Å (**2A**), 3.224(3) Å (**2B**), and 3.156(3) Å (**2C**). However, complex **3** exhibited only one dinuclear molecule with an intermetallic separation of 3.131(17) Å, in the low range of distances observed in other platinum-butterfly complexes with the same body (3,5-dmpz) but bearing different wings (3.128–3.203 Å).^26,38−40^ Unfortunately, no good quality crystals were obtained for **4**, but we could confirm the atom connectivity. Two different molecules with a Pt–Pt separation of 3.054 and 2.982 Å were found in the asymmetric unit. Therefore, once again it can be established that when the steric demand of the bridging pyrazolate increases the platinum centers are pushed closer together, like in other butterfly-like platinum complexes reported by Thompson et al.,^26^ Umakoshi et al.,^40^ and Strassner et al.^32^ An extended description of these molecules has been included in the SI (see Table S2 and Figures S5–S7).

### Theoretical Calculations

DFT calculations on the GS and the lowest adiabatic triplet excited state (T_1_) PESs for **1**–**4** were performed, and the geometries of relevant stationary points, such as, e.g., local minima and transition states (TS), were optimized (see SI for computational details) accounting for solvent effects in THF.

**Figure 2:**
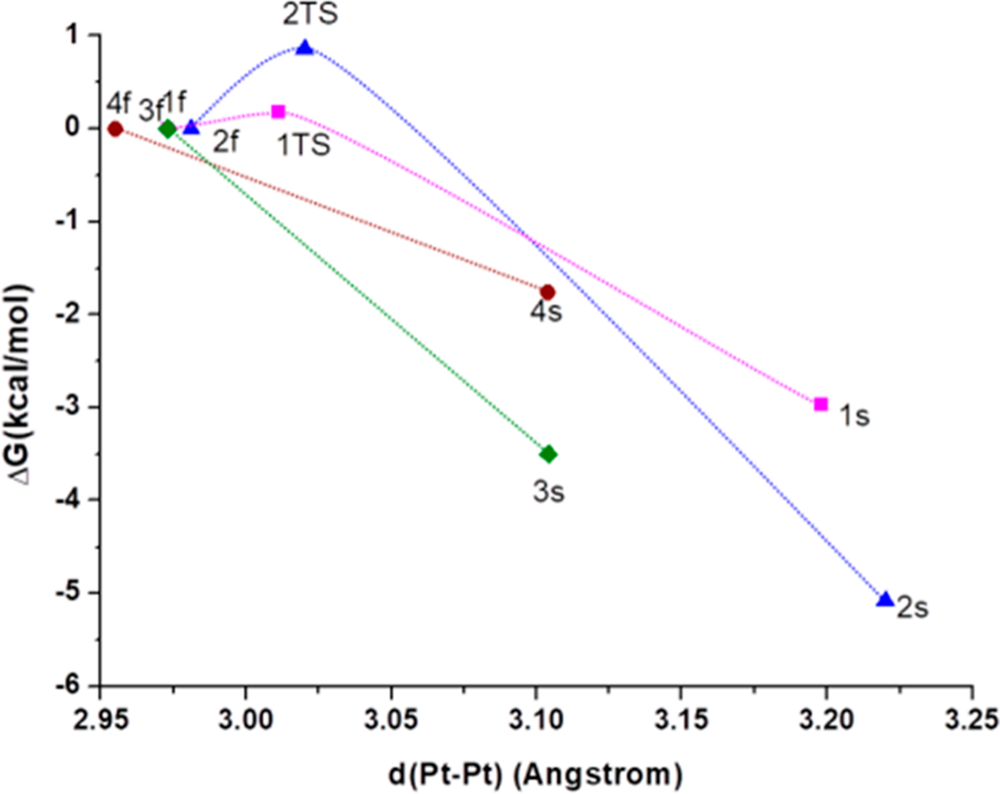
Calculated relative energy profile (PCM-M06/6-31G(d) and MWB60(Pt)) in the GS for the interconversion between the **1f**–**4f** and **1s**–**4s** conformers. Values calculated in THF.

For all of the complexes, the geometries of two close-lying local minima were optimized in the GS PES (see Figure S8 in SI) which corresponded to the butterfly spread structures **1s**–**4s** and the butterfly folded ones **1f**–**4f**. Those corresponding to the butterfly spread conformers **1s**–**4s** show long Pt–Pt distances (3.10 Å for **4s**, 3.10 Å for **3s**, <3.20 Å for **1s**, <3.22 Å for **2s**) and intramolecular C^∧^C* separations (>4.5 Å) following the same trend as the one observed in the experimental values. Also, they are characterized by a small Pt–Pt bond order (BO: 0.036 **4s**, <0.106 **3s**, <0.110 **1s**, <0.111 **2s**). On the other hand, the GS optimized geometries corresponding to the butterfly folded conformers **1f**–**4f** are characterized by shorter Pt–Pt distances (2.96 Å for **4f**, <2.97 Å for **3f**, 2.97 Å for **1f**, <2.98 Å for **2f**) and intramolecular C^∧^C* contacts (<3.8 Å) along with larger Pt–Pt bond orders (BO: 0.170 **4f**, 0.174 **3f**, <0.228 **1f**, <0.233 **2f**) than those of **1s**–**4s**. The computed energy profiles in the GS PES are shown in Figure 2. For all compounds, the conformers **1s**–**4s**, featuring longer Pt–Pt distances, are more stable than the conformers **1f**–**4f** (Δ*G*: 0.076 eV (1.76 kcal/mol) for **4**, 0.129 eV (2.97 kcal/mol) for **1**, 0.152 eV (3.50 kcal/mol) for **3**, and 0.220 eV (5.08 kcal/mol) for **2**). Especially, this is remarkable for **2s**, which bears the longest Pt–Pt separation and the largest dihedral angle between the two platinum coordination planes. In addition, for complexes bearing bulkier Rpz units (**3** and **4**), their **3s** and **4s** minima are stabilized at shorter Pt–Pt distances than **1s** and **2s**.

Furthermore, for complexes **1** and **2**, we have successfully located the transition state (TS) associated with the interconversion between both conformers (see their optimized geometries in Figure S8). These TSs lie exemplarily 0.0077 eV (0.18 kcal/mol) above **1f** and 0.037 eV (0.86 kcal/mol) above **2f** (see Figure 2). Their optimized geometries display Pt–Pt distances which lie in between those found for the butterfly folded and butterfly spread optimized minima. In the case of **1**,^33^ a small Δ*G* (**1s**/**1f**) value along with a small activation barrier supports, within the experimental error, a fast thermal equilibration in the ground state PES, thus resembling an intramolecular *butterfly flapping-like* motion.

These results are fully consistent with the presence of both conformers in solution, with the butterfly spread being the predominant one. Attempts to optimize the geometries of the TSs for the interconversion between conformers of complexes **3** and **4** were unsuccessful. In view of this piece of evidence, the flapping process likely occurs in a barrierless manner for the latter complexes.

**Figure 3:**
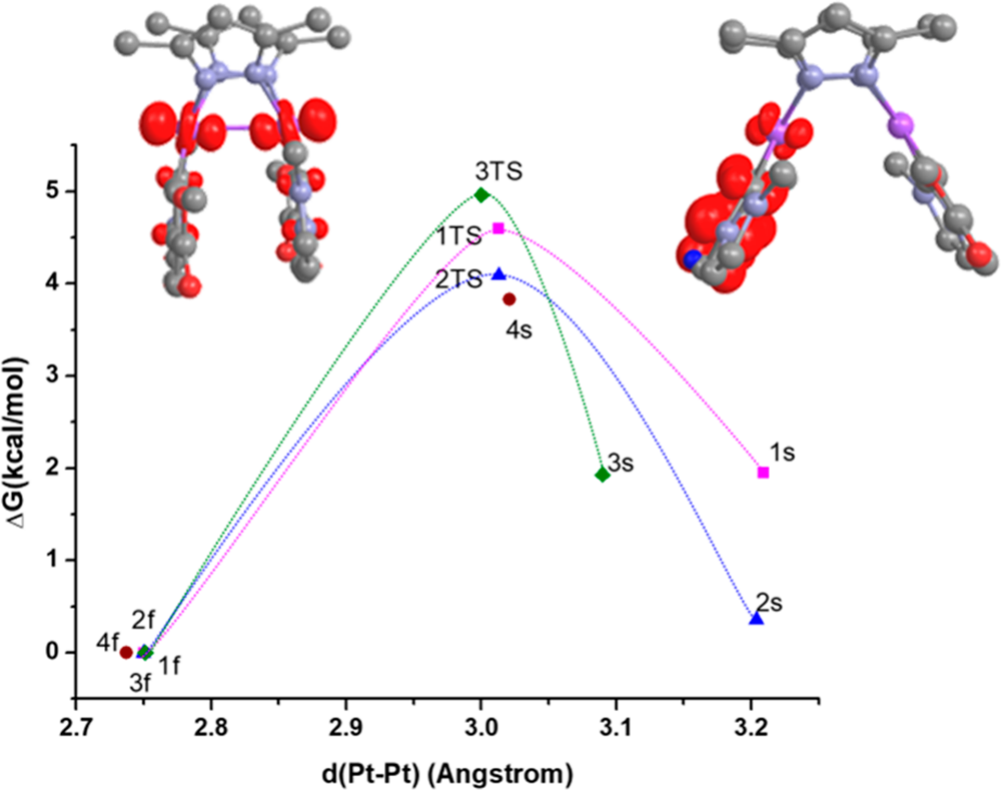
Calculated relative energy profile (PCM-M06/6-31G(d) and MWB60(Pt)) in the lowest adiabatic triplet excited state (T_1_) for the interconversion between the **1f**–**4f** and **1s**–**4s** conformers. Values calculated in THF. Spin density distribution plots of **3f** (left) and **3s** (right).

Let us now discuss the results for the calculations on the lowest adiabatic triplet excited state (T_1_) PES. The geometries of two local minima, i.e., **s**/**f**, were optimized for all of the complexes (Figure 3 and Figure S9 in the SI). The optimized geometries for the butterfly spread conformers, **1s**–**4s**, show Pt–Pt distances (3.02 Å for **4s**, <3.09 Å for **3s**, <3.20 Å for **2s**, <3.21 Å for **1s**) and Pt–Pt bond orders (BO: 0.116 **3s**, 0.110 **2s**, 0.102 **4s**, 0.099 **1s**), similar to those observed for most of them in the GS.

However, the T_1_ optimized geometries for the butterfly folded conformers, **1f**–**4f**, exhibit intermetallic separations (2.74 Å for **4f**, <2.75 Å for **1f**–**3f**), which are shortened by ca. 0.22 Å with respect to those in the GS, and Pt–Pt bond orders (BO: 0.586 **4f**, 0.591 **3f**, 0.626 **2f**, 0.621 **1f**), which are increased by 0.4 with respect to those in the GS. The calculated spin density distribution for **1s**–**4s** indicates a mixed ^3^IL/^3^MLCT [π(C^∧^C*) → π*(C^∧^C*)]/[5d(Pt) → π*(C^∧^C*)] character for their T_1_ states (see Figures 3 and S10) but a ^3^MMLCT [dσ*(Pt–Pt) → π*(C^∧^C*)] character for the T_1_ states of **1f**–**4f**. Note that the changes in the Pt–Pt distances and the BO values from the GS to T_1_ states in the butterfly folded conformers **1f**–**4f** almost agree with a one-electron excitation from the dσ*(Pt–Pt) orbital.

Like in the previously reported C,N-cycloplatinated butterfly-like complexes by Ma et al.,^28^ as the steric bulk of the Rpz ligand increases, their spread-like minima (^3^IL/^3^MLCT) display shorter Pt–Pt bond distances (compare e.g., **1s** and **2s** vs **3s** and **4s** in Figure 3). Importantly, comparing the **1s**–**4s** and the **1f**–**4f** optimized geometries in their T_1_ states, there is a considerable shortening of the Pt–Pt distances in the folded-like structures. The change of excited state character when going from the **1s**–**4s** minima (^3^IL/^3^MLCT) to the **1f**–**4f** ones (^3^MMLCT) leads to an extra stabilization of the latter conformers^41^ by 0.085 eV (1.95 kcal/mol), 0.015 eV (0.36 kcal/mol), 0.084 eV (1.93 kcal/mol), and 0.166 eV (3.83 kcal/mol) for complexes **1**–**4**, respectively. Note also that a certain amount of Pt–Pt bonding is only possible in the T_1_ state but not in the GS.

All in all, a comparison of the GS and T_1_ energy profiles reveals that an opposite trend is obtained in the relative stability of folded and spread conformers, the former being clearly more stabilized in their T_1_ states, regardless of the steric hindrance of the bridging Rpz, but specially for complex **4**. In addition, we located the transition states (TSs) for the interconversion between conformers in the T_1_ state for **1**–**3**, which are shown in Figure S9. These TSs all bear one imaginary frequency associated with the interconversion between both conformers. These TSs lie 0.115 eV (2.65 kcal/mol), 0.162 eV (3.73 kcal/mol), and 0.132 eV (3.04 kcal/mol) above the local minima **1s**–**3s**, respectively. These energy barriers for PSC are larger than those for the flapping-like intramolecular motion in the GS

**Figure 4:**
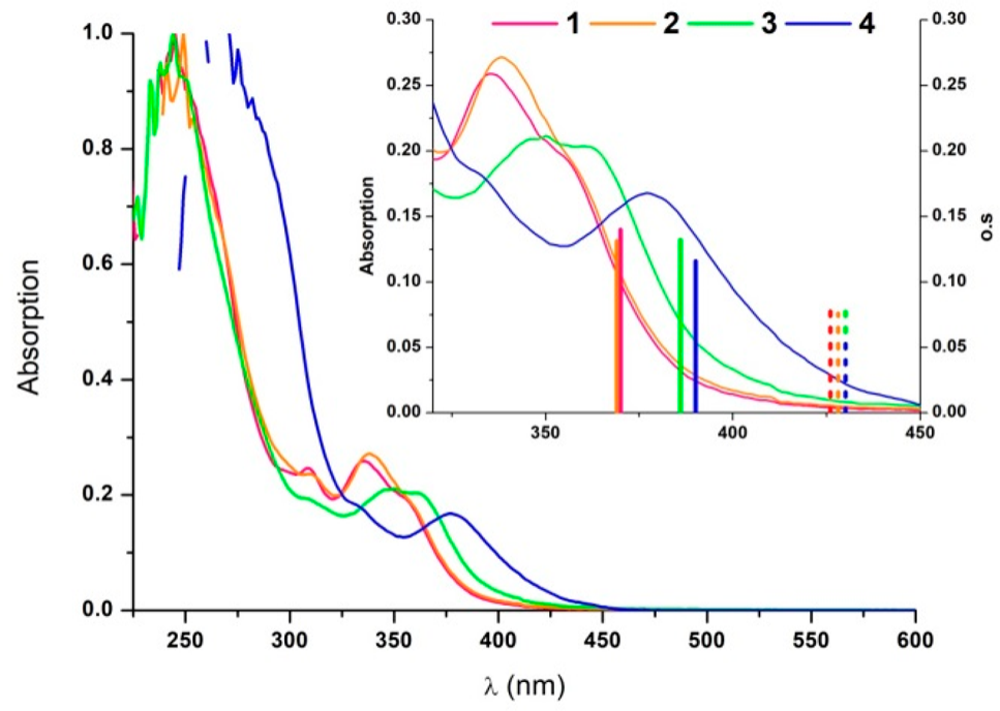
UV–visible spectra (path length: 1 mm) of **1**–**3** in 2-MeTHF 10^–3^ M and **4** in 2-MeTHF 10^–5^ M (path length: 1 cm). Inset: Expanded view of the UV–vis spectra along with the TD-M06/6-31G(d) and MWB60(Pt) S_0_ → S_1_ transitions of the butterfly spread (solid bars) and butterfly folded (dashed bars) conformers.

The absorption properties of **1**–**4** were also investigated with PCM-TD-DFT calculations in the presence of THF (see details in the SI). The results are collected in Tables S3 and S4 and Figure S11. The frontier molecular orbitals (Figure S11) for **1s**–**4s** and **1f**–**4f** along with the energies of their lowest singlet excited states were also calculated (see inset of Figure 4 and Table S4 in SI). The lowest singlet excited states have predominant HOMO to LUMO character and can be described mainly as ^1^MLCT/^1^IL [5d(Pt) → π*(C^∧^C*)]/[π(C^∧^C*) → π*(C^∧^C*)] for **1s**–**4s**, while some additional ^1^MMLCT [dσ*(Pt–Pt) →π*(C^∧^C*)] character is found for those of **1f**–**4f**. The vertical ΔSCF-M06 emission energies from the T_1_ optimized geometries were calculated as well, rendering values of ca. 510 nm for **1s**–**4s** and of ca. 570 nm for **1f**–**4f** (see Table S4 in SI).

### Photophysical Properties

The absorption and emission properties of **1**–**4** were investigated and explained on the basis of the DFT calculations. The UV–vis spectra of **1**–**4** (Figure 4 and Table S26 in SI) do not show differences between diluted (10^–5^ M) and concentrated solutions (10^–3^ M). They show their lowest-energy absorption bands (ε ∼ 9 × 10^3^ M^–1^ cm^–1^) in the range 325–390 nm. These absorptions bands match the S_0_ → S_1_ transitions calculated for the butterfly spread molecules **1s**–**4s** (see Figure 4), which are the predominant species according to the calculations.

However, in spite of the low contribution of the Rpz to the frontier molecular orbitals (FMOs), this absorption appears clearly red-shifted as the bulkiness of the R groups on the bridging pyrazolate increases. So, for species **3s** and **4s**, exhibiting shorter intermetallic distances and smaller interplanar angles in the GS, some ^1^MMLCT [dσ*(Pt–Pt) → π*(C^∧^C*)] character could be reasonably attributed (see Figures S8 and S11).

**Figure 5:**
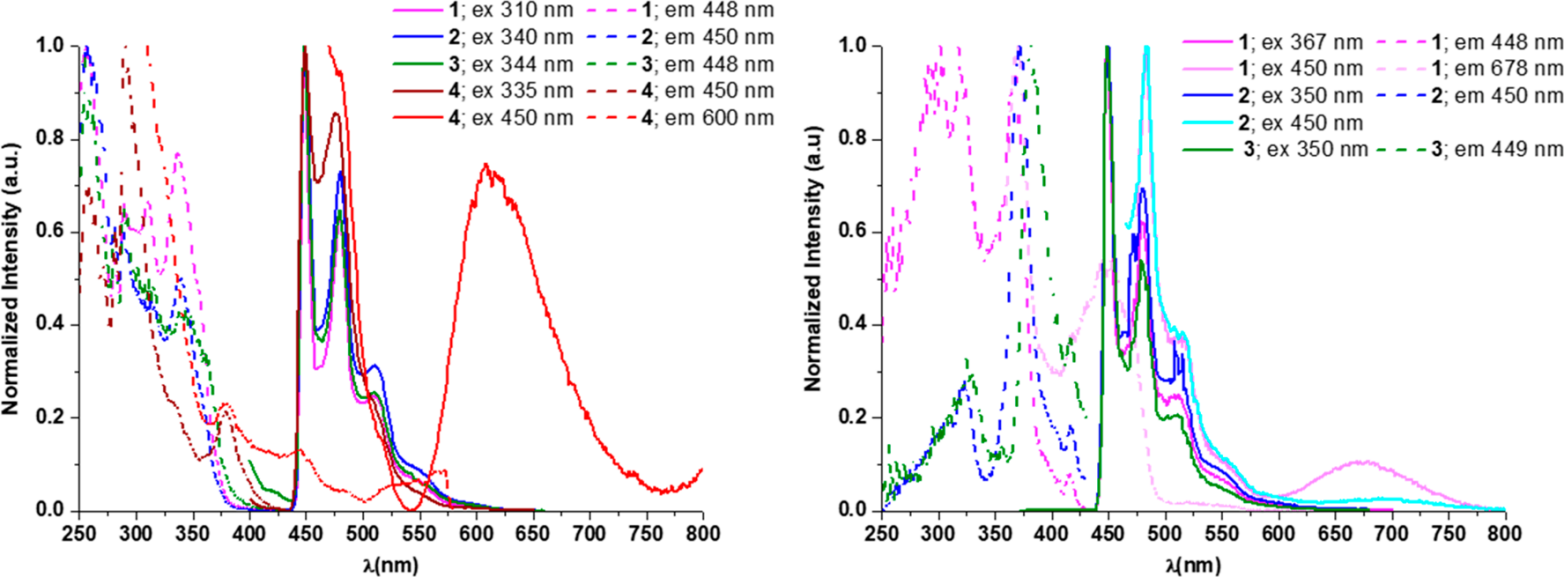
Normalized excitation (dotted lines) and emission (solid lines) spectra at 77 K under an Ar atmosphere. Left: **1**–**4** in 2-MeTHF 10^–5^ M. Right: **1**–**3** in 2-MeTHF 10^–3^ M.

Diluted solutions (10^–5^ M) of **1**–**4** in 2-MeTHF were fast-cooled to 77 K. Upon excitation at λ ≤ 340 nm, each of their emission spectra were characterized by highly structured emission bands with λ_max_ ∼ 450 nm and vibronic spacings [∼1450 cm^–1^], likely corresponding to the C=C/C=N bond stretching modes of the cyclometalated NHC ligands (Figure 5, left). The emission energies are not affected by the nature of the Rpz ligands, and they are very similar to those observed in the mononuclear compounds bearing the same “(C^∧^C*)Pt” fragment.^34,35,42,43^

The computed emission energies for the butterfly spread conformers, i.e., **1s**–**4s**, agree better with the experimental findings at 77 K than those calculated from **1f**–**4f**. Thus, these results highlight that the barriers for interconversion between **s/f** conformers at the T_1_ state (see Figure 3) are large enough to prevent their thermal equilibrium at 77 K.

Complexes **1** and **4** show additional excitation and emission bands at lower energies (λ_exc_ ∼ 450 nm, λ_em_ > 600 nm), attributable to the butterfly folded molecules (calculated *S*_1_ ∼ 426 nm and *T*_1_ = 572 nm for **1f**; *S*_1_ ∼ 429 nm and *T*_1_ = 570 nm for **4f**), although for **1** they are only perceptible in concentrated solutions (10^–3^ M; Figure 5, right). The coexistence of butterfly spread and butterfly folded molecules for **1** and **4** is in accordance with the small Δ*G* value computed between the two conformers, **s**/**f** in the ground state (Δ*G*: 0.076 eV (1.76 kcal/mol) **4s**/**4f**, 0.129 eV (2.97 kcal/mol) **1s**/**1f**) within the margin of error for the calculation of the energies of similar complexes with the M06 functional (MUE = 2.48 kcal/mol, see SI). For complexes **2** and **3** because of the greater Δ*G* between them (0.220 eV (5.08 kcal/mol) **2s**/**2f**, 0.152 eV (3.50 kcal/mol) **3s**/**3f**), it seems to be more unlikely and undetectable at 77 K.

**Figure 6:**
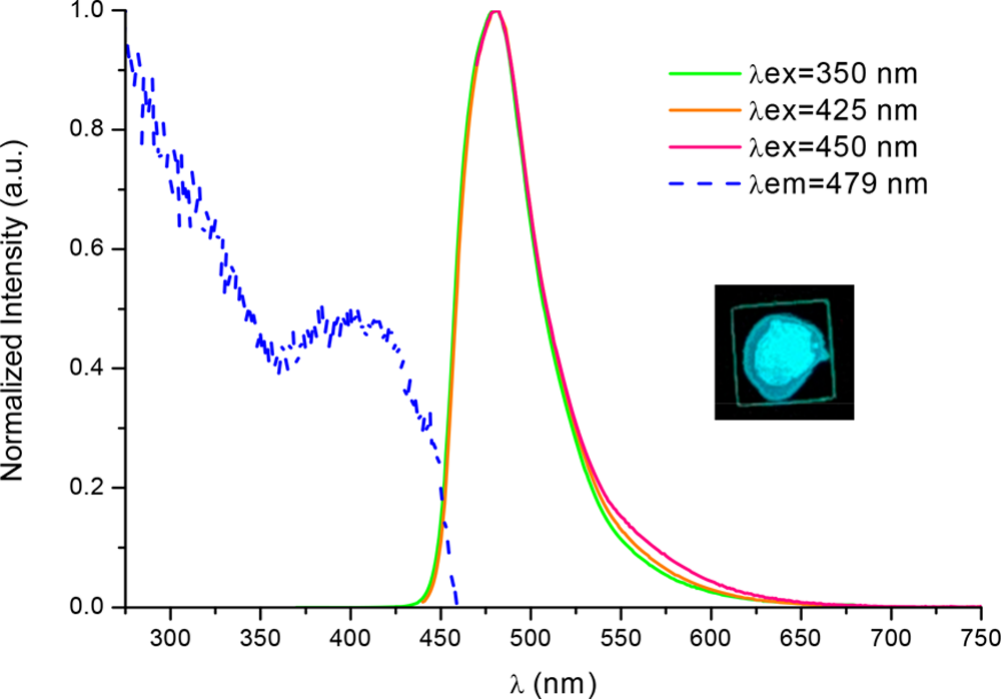
Normalized emission and excitation spectra of complex **4** in 5 wt % PMMA film in the air, Picture was taken under 365 nm UV light.

In 5 wt % doped PMMA films in the air, excitation of complexes **1**–**4** at λ ≤ 380 nm affords intense sky-blue emissions with quantum yields of 72.0% **1**, 83.4% **2**, 79.0% **3**, and 85.9% **4** (see Table 1). These emissions match with those observed in 2-MeTHF (10^–5^ M) at 77 K. The slight blue shift in the emission spectra upon cooling is in accordance with an emissive state of ^3^IL/ ^3^MLCT character. The excitation of these films at longer wavelengths, up to 450 nm, render less intense but matched emission bands (see Figure 6; Table S27 and Figure S12 in the SI). The short radiative decay of these emissions at room temperature should be noted, which are similar to those observed in analogous complexes [{Pt(C^∧^C*)(μ-Rpz)}_2_](HC^∧^C* = 3-dibenzofuran-4-yl-1-methyl-3*H*-imidazol-2-ylidene, imidazopyridine-2-ylidene; R = H, Me, tBu)^31,32^ but clearly shorter than those measured for mononuclear compounds containing the same “(C^∧^C*)Pt” fragment.^42,43^

**Table 1:** Photophysical Data for **1**–**4** in PMMA Films and Solid State in the Air at 298 K

C	media	λ_exc_ (nm)	λ_em_ (nm)	CIE (x;y)	τ (μs)	φ(%)	*k*_r_^b^	*k*_nr_^c^
1	PMMA^a^	390	483_max_, 517_sh_, 567_sh_	0.18; 0.32	3.7	20	5.4 × 10^4^	21.6 × 10^4^
	PMMA^a^	350	483_max_,517_sh_, 567_sh_	0.18; 0.32		72		
	solid	390	469, 527_sh_, 556_max_	0.41; 0.52	0.4 (20%)	3	2.5 × 10^4^	79.2 × 10^4^
					1.4 (80%)			
2	PMMA^a^	390	469, 485_max_, 524_sh_	0.16; 0.29	3.5	54	15.4 × 10^4^	13.1 × 10^4^
	PMMA^a^	370	473, 492_max_, 536_sh_	0.16; 0.27		83		
	solid	390	472, 527_sh_, 559_max_	0.41; 0.53	0.3 (22%)	3	3.2 × 10^4^	103.2 × 10^4^
					1.1 (78%)			
3	PMMA^a^	390	464, 484_max_, 523_sh_	0.15; 0.25	3.4	53	15.7 × 10^4^	13.8 × 10^4^
	PMMA^a^	380	464, 484_max_, 523_sh_	0.15; 0.25		79		
	solid	390	468, 487_max_	0.19; 0.35	0.3 (32%)	16	30.1 × 10^4^	158.1 × 10^4^
					0.6 (68%)			
	ground	390	468, 492_max_, 519	0.29; 0.47	0.2 (20%)	6	11.2 × 10^4^	175.4 × 10^4^
	solid				0.6 (80%)			
4	PMMA^a^	390	480_max_	0.14; 0.26	2.2	69	31.7 × 10^4^	14.1 × 10^4^
	PMMA^a^	380	480_max_	0.14; 0.26		86		
	solid	390	469,482_max_, 553_sh_	0.24; 0.37	0.5 (30%)	29	32.9 × 10^4^	80.7 × 10^4^
					1.1 (70%)			
	ground	390	553_max_	0.39; 0.55	1.1 (33%)	51	28.3 × 10^4^	27.2 × 10^4^
	solid				2.2 (67%)			

a 5 wt %.

b Radiative decay rate constant given as *k*_r_ = φ/τ_exp_.

c *k*_nr_ = (1 – φ)/τ_exp_.

All of these pieces of evidence highlight a greater metallic contribution to the excited state and, then, a greater ^3^MLCT character of the blue emissions of complexes **1**–**4** in PMMA films compared to those of the mononuclear complexes.^42,43^

Note that the large radiative rate constant values (*k*_r_, Table 1) support this. Also, compared to **2**–**4** (*k*_r_ > 1.0 × 10^5^ s^–1^), a smaller radiative rate constant was obtained for **1** (*k*_r_ = 1.0 × 10^4^ s^–1^), suggesting that the spin–orbit coupling (SOC) efficiency was lower because of a larger energy separation between the manifold of triplet and singlet states.^44^ Exemplarily, from the absorption and the PMMA emission data, the energy differences between S_1_ and T_1_ (Δ*E*_S–T_ = 0.93 eV **1**, 0.81 eV **2**; 0.75 eV **3**, 0.70 eV **4**) were found to follow the same trend observed for the *k*_r_.

In solution at room temperature, compounds **1**–**4** are scarcely luminescent even in an argon atmosphere, a usual feature for blue-emitting Pt(II) complexes since the population of dd* states and formation of exciplexes are very common thermal quenching processes.^43^ However, in a fluid solution of 2-MeTHF (10^–5^ M) at room temperature under an argon atmosphere (Figures S13 in SI), excitation in the low-energy absorption range (λ ≤ 380 nm) renders for complexes **1** and **2** a weak emission from **1s** and **2s**, for complex **3** a dual emission with maxima at 456 and 552 nm, likely corresponding to **3s** and **3f**, and for complex **4** an emission with a maximum at 559 nm arising from **4f**, according to theoretical calculations.

**Scheme 2:**
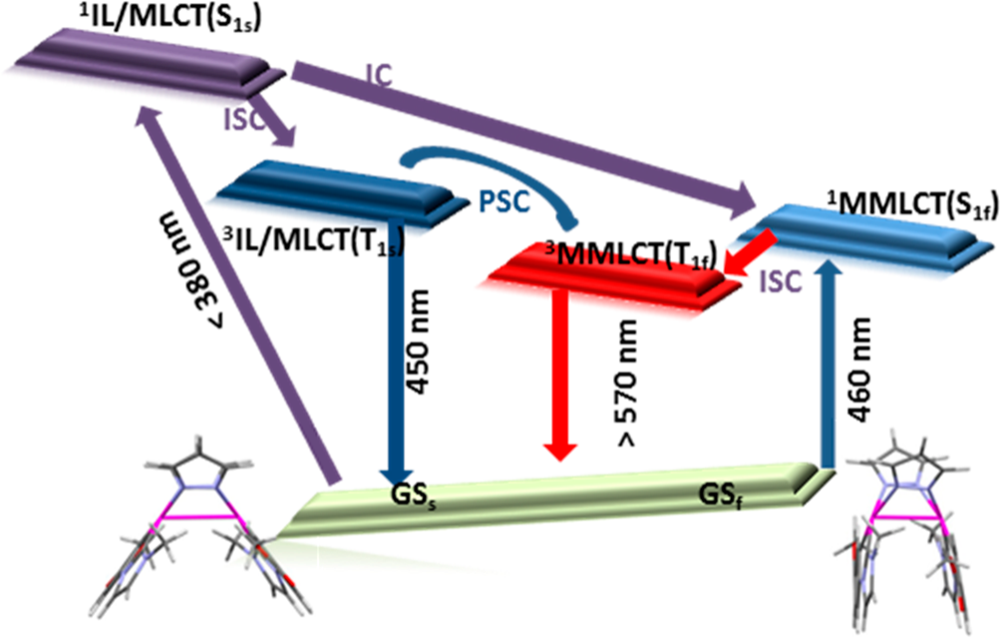
Schematic Diagrams of Photophysical Processes Based on the Steady-State Excitation and Emission Spectra along with the Results of the Theoretical Investigations

In summary, photoexcitation of complexes **1**–**4** at λ < 380 nm allows the major **1s**–**4s** conformers to reach the high-energy ^1^IL/MLCT excited state; then, by a rapid intersystem crossing (ISC), the ^3^IL/MLCT (Ts) state will be populated, which is calculated to have a similar Pt–Pt separation than that of its corresponding ground state geometry (see Scheme 2). In fluid solution, where the geometries of neither ground states nor those of the excited states are constrained, a photoinduced structural change (PSC) process between T_s_ and T_f_ conformers could happen depending on both, Δ*G* (T_f_–T_s_) and the PSC energy barrier.^28^ In the case of **4**, the computed barrierless PSC process along with the large Δ*G* values (T_4f_–T_4s_ = −0.166 eV, −3.83 kcal) leads to T_4f_ almost in an exclusive manner. This piece of evidence explains why the emission from T_4f_ is the only one observed experimentally. In the case of complex **3**, characterized by a smaller Δ*G* (T_3f_–T_3s_ = −0.84 eV, −1.93 kcal) and a non-negligible PSC barrier (0.132 eV, 3.04 kcal), a thermal equilibrium between T_3s_ and T_3f_ is likely at room temperature, thus explaining its dual emission.

In the case of complex **2** with a lower Δ*G* (T_2f_–T_2s_ = −0.015 eV, −0.36 kcal) but larger energy barrier (0.162 eV, 3.73 kcal/mol), the PSC seems not to take place and the emission arises only from T_2s_. For complex **1**, with Δ*G* (T_1f_/T_1s_= 0.085 eV, 1.95 kcal) and the PSC energy barrier (0.115 eV, 2.65 kcal) on the same order of magnitude as those calculated for complex **3**, the PSC was expected to occur, but, the emission arises only from T_1s_.

In this case, we recently reported that internal conversion (IC) from ^1^IL/MLCT to ^1^MMLCT competes with ISC to ^3^IL/MLCT.^33^ Therefore, a faster quenching of the ^1/3^MMLCT states of complex **1**, as compared to that occurring in complexes **3** and **4**, enabled by the lack of steric hindrance of the reactive positions in complex **1**, could account for the absence of this low-energy emission.

In rigid media (2-MeTHF 10^–5^ M at 77 K or PMMA films), photoexcitation of complexes **1**–**4** at λ < 380 nm leads in an analogous manner to the emission from the higher-lying triplet state ^3^IL/MLCT, despite the greater stability of the ^3^MMLCT state. This indicates that the energy barriers to connect the T_s_/T_f_ wells are large enough to prevent the PSC in 2-MeTHF at 77 K, and it is also in agreement with PMMA being a rigid glass at r.t. (Tg = 378 K).^29^ Notably, the frozen glass environment leads to a deceleration of the nonradiative pathways, thus leading to large PLQY values in PMMA films (see Table 1).

On the other hand, irradiation at λ > 400 nm will populate the low-energy states of the minor **1f**–**4f** conformers, ^1^IL/MLCT with some ^1^MMLCT character (see right panel in Scheme 2). A fast ISC to the close-lying triplet state ^3^IL/MLCT^45^ would lead to the high-energy emission, which is the only one observed in 5 wt % PMMA films of **1**–**4**. The low PLQYs when compared with those observed by irradiation at λ < 380 nm (see Table S27 and Figure S12 in SI) are in agreement with the low ratio of butterfly folded molecules in the samples but still being significant. Therefore, for complexes **2**–**4**, the existence of close-lying ^1^IL/MLCT/MMLCT–^3^IL/MLCT states makes it possible to get intense blue emissions (PLQY: 40%–60%) from doped films by irradiation with wavelengths in the visible region.

### Mechanoluminescence in the Solid State

The as-prepared powders of **1** and **2** are scarcely emissive. They exhibit a weak (PLQY < 5%), broad, structureless emission centered at λ ∼ 555 nm (Table 1 and Figure S14) with a shoulder at λ ∼ 450 nm. The shape and energy of the main band could match with the one arising from the low-lying triplet state for the butterfly folded molecules that is the ^3^MMLCT state. Yet, the role of excimeric ^3^π–π* states can not be fully disregarded, given the extended and numerous intermolecular π–π interactions observed in the single-crystal X-ray structures of **1** and **2** (see Figure S6) and the lower quantum yield of compounds **1** and **2** in solid state compared to those in 5 wt % PMMA films. Keeping in mind that other mononuclear Pt(II) compounds containing cyclometalated NHCs reported previously^42,46,47^ exhibit a similar behavior mainly as a consequence of π–π intermolecular interactions, it seems likely that the emission of **1** and **2** arises from excimeric ^3^π–π* states with intermolecular π–π interactions affording efficient nonemissive deactivation channels^12^ through an *aggregation-caused quenching* (ACQ) effect.^9,48^ Neither the excitation nor the emission spectra of **1** and **2** exhibit changes after grinding the solids with a mortar and pestle (see Figure S15 for **2** as an example). However, complexes **3** and **4** exhibit mechanoluminescence in the solid state. After grinding, the pale-yellow solids do not visually change their colors, but their photoluminescence changes from blue to yellowish-green. Before being ground, a powdered sample of **3** exhibits a sky-blue emission, similar but weaker than that exhibited in PMMA film (5 wt %), which we attribute to ^3^IL/MLCT. After grinding, the emission becomes green (Figure S16) due to the presence of an intense lower-energy band with λ ∼ 540 nm that could be assigned to the ^3^MMLCT state of molecules with the butterfly folded configuration in accordance with the theoretical calculations. However, in view of the intermolecular π–π interactions observed in the single-crystal X-ray structure of **3** (see Figure S6) and the decreased PLQY upon grinding, the participation of excimeric ^3^π–π* states to the low-energy band cannot be ruled out.^9,48^

**Figure 7:**
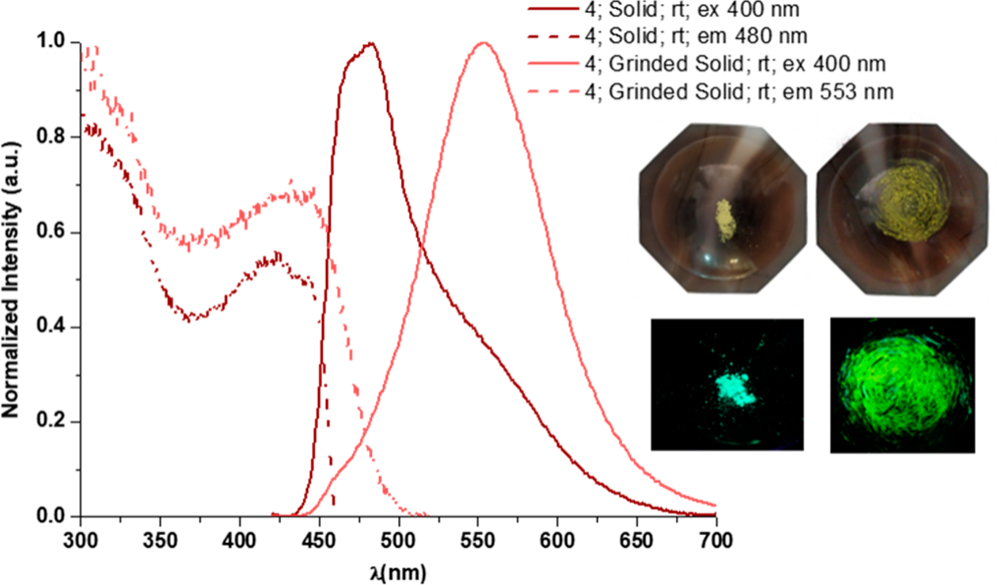
Normalized emission and excitation spectra of complex **4** in the solid state in the air at room temperature. Pictures were taken under 365 nm UV light.

In case of compound **4**, photoexcitation of as-prepared powder leads to a greenish-blue emission with λ_max_ at 480 nm and an incipient shoulder at 553 nm that can be assigned to ^3^IL/MLCT and ^3^MMLCT emissions, respectively, in accordance with the theoretical calculations and with the ^3^MMLCT emission observed for [{Pt(C^∧^C*)(μ-NPh-CH-NPh)}_2_] exhibiting a short Pt···Pt distance, of about 2.8 Å.^24^ Mechanical grinding resulted in a suppression of the ^3^IL/MLCT band along with an increase of the ^3^MMLCT one and, unlike complex **3**, an enhancement of the PLQY (see Figure 7). As a result, the photoluminescence of powdery samples of **4** is intensified and changed from greenish-blue to yellowish-green upon grinding.

From the excitation and emission spectra, it seems that the as-prepared powder of **4** shows phosphorescence from the two kinds of conformers, **4s** and **4f**, that from **4s** being predominant. This is in agreement with the butterfly spread conformer, **4s** being the major one in the GS and the fact that no PSC can take place in rigid media. Mechanical grinding seems to induce changes in the GS that somehow shorten the Pt–Pt distances and enforces the intramolecular Pt–Pt interactions, in such a way that in the ground solid, **4-g**, the phosphorescence arises mainly from **4f**.

Structural changes involving the intramolecular Pt–Pt separation in the GS as the origin of mechanoluminesce seems plausible on the bases of experimental and theoretical data, and once other causes were dismissed, like desolvation, since there is no solvent embedded in the solid (see CHN elemental analysis and NMR experiments) or intermolecular interactions, we proved that the luminescence spectrum of **4** in 40 wt % doped PMMA films match that at 5 wt % (see Figure S17), and its PLQY drops to 40%.

The ground solid, **4-g**, undergoes the reverse change partially upon cooling to 77 K, as deduced from the emission and excitation spectra collected at room temperature and 77 K (Figure S18).

**Figure 8:**
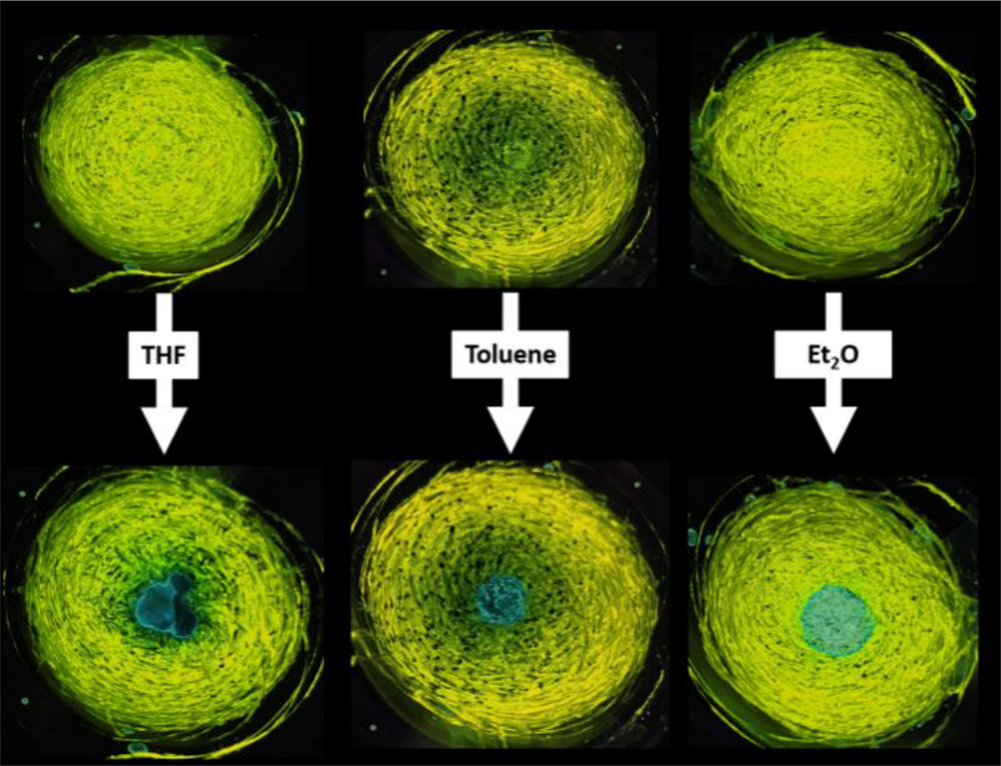
Photographic images of mechanical grinding samples of **4** in response to solvent treatment taken under 365 nm UV light

Structural changes involving the intramolecular Pt–Pt separation in the GS were reported for the thermochromic platinum-butterfly compound [{Pt(ppy)(μ-Ph_2_pz)}_2_],^29^ but those induced by mechanical grinding have never been reported. In this case, like in complex **4**, elongation of the Pt–Pt distance occurs when the temperature drops. Also, the transformations resulted to be reversible by the addition of THF, toluene, or diethyl ether to the ground samples of **3** and **4** that led to the recovery of the blue emission (see Figure 8 and S19), and thus presumably restoring the previous structure arrangement.

Therefore, it could be argued that the bulkiness of the μ-pyrazolates has a strong impact not only on the luminescence, but also on the mechanoluminiscence of these platinum butterflies in the solid state. As the bulkiness increases, the intermolecular π–π interactions become more hindered, affording less efficient nonemissive deactivation channels and consequently a more efficient emission. In addition, as the steric demand of the μ-pyrazolates increases, the Pt–Pt interaction, enhanced by mechanical stimulation, causes a bathochromic shift of the emission (2750 cm^–1^**4**) along with a remarkable increment of its PLQY.^49−54^

## Conclusions

Photoluminescence and DFT calculations on a new series of platinum butterflies, [{Pt(C^∧^C*)(μ-Rpz)}_2_] (Rpz: pz **1**, 4-Mepz **2**, 3,5-dmpz **3**, 3,5-dppz **4**) containing a cyclometalated NHC in their wings, prove the presence of two conformers in the ground state at room temperature, the butterfly spread, **1s**–**4s** and the butterfly folded, **1f**–**4f** ones, which are characterized by long and short Pt–Pt separations, respectively. DFT calculations revealed that the former are the more stable in the GS but, in most of them, low Δ*G* (**s**/**f**) and low energy barriers in solution of THF support a fast thermal equilibration in the ground state PES, thus resembling an intramolecular *butterfly flapping-like* motion. By contrast, the butterfly folded, **1f**–**4f**, conformers are the more stable in the T_1_PES. In 5 wt % doped PMMA films in the air, these complexes show intense sky-blue emissions (PLQY: 72.0–85.9%) upon excitation at λ ≤ 380 nm mainly arising from an ^3^IL/MLCT excited state, the first triplet state of the major butterfly spread conformer **1s**–**4s**, in accord with no PSC occurring in a rigid matrix. The existence of close-lying ^1^IL/MLCT/MMLCT-^3^IL/MLCT states for the **1f**–**4f** species enables the obtaining of intense blue emissions (PLQY: 40%–60%) under excitation with wavelengths in the visible region, up to 450 nm.

In addition, it could be argued that the bulkiness of the μ-pyrazolates has a strong impact on the luminescence and mechanoluminescence of these platinum butterflies in a solid state. In complexes **3** and **4**, the intermolecular π–π interactions become more hindered than in complexes **1** and **2**, affording more efficient emissions. In addition, in the 3,5-dppz derivative, **4**, mechanical grinding causes a bathochromic shift of the emission from greenish-blue to yellowish-green along with a remarkable increment of its PLQY. This mechanoluminescence mechanism has been associated with an intramolecular structural change in the GS that somehow shortens the Pt–Pt distances and enhances the Pt–Pt interactions in such a way that the thermal butterfly flapping can be induced by mechanical grinding.

## Supporting Information Available

General procedures and materials; crystallographic data; computational methods, NMR figures; description of single-crystal X-ray structures, DFT and PCM-DFT studies, additional figures for the photophysical study (DOCX)

## Accession Codes

CCDC 2077779–2077780 contain the supplementary crystallographic data for this paper. These data can be obtained free of charge via www.ccdc.cam.ac.uk/data_request/cif, or by emailing data_request@ccdc.cam.ac.uk, or by contacting The Cambridge Crystallographic Data Centre, 12 Union Road, Cambridge CB2 1EZ, UK; fax: +44 1223 336033.
