# Supplementary material for: Chameleonic Photo- and Mechanoluminescence in Pyrazolate-Bridged NHC Cyclometalated Platinum Complexes
Source: Inorg Chem. 2021 Aug 2;60(16):12274–84. doi: 10.1021/acs.inorgchem.1c01470 (PMC8892954; doi:10.1021/acs.inorgchem.1c01470)
Supplement: Supplementary file 1 — ic1c01470_si_001.docx [file ic1c01470_si_001.docx]

Supporting Information

**Chameleonic Photo- and Mechanoluminescence in Pyrazolate-Bridged NHC Cyclometalated Platinum Complexes**

Violeta Sicilia,*^a^ Lorenzo Arnal,^b^ Daniel Escudero,*^c^ Sara Fuertes*^b^ and Antonio Martin^b^

*^a^Departamento de Quimica Inorganica, Escuela de Ingenieria y Arquitectura de Zaragoza, Instituto de Sintesis Quimica y Catalisis Homogenea (ISQCH), CSIC - Universidad de Zaragoza, Campus Rio Ebro, Edificio Torres Quevedo, 50018, Zaragoza (Spain)*. E-mail: [*sicilia@unizar.es*](mailto:sicilia@unizar.es)

^b^Departamento de Quimica Inorganica,, Facultad de Ciencias, Instituto de Sintesis Quimica y Catalisis Homogenea (ISQCH), CSIC - Universidad de Zaragoza, Pedro Cerbuna 12, 50009, Zaragoza (Spain). E-mail: [sfuertes@unizar.es](mailto:sfuertes@unizar.es)

^c^Department of Chemistry, KU Leuven, Celestijnenlaan 200f - box 2404, 3001 Leuven. E-mail: [daniel.escudero@kuleuven.be](mailto:daniel.escudero@kuleuven.be)

CONTENTS Page

1. Experimental section: S3

1.1 General procedures and materials S3

1.2 Crystallographic data (Table S1) S3

1.3 Computational Methods S5

2. Results and discussion S7

2.1 NMR Figures for characterization (Figures S1 − S4) S7

2.2 Single-crystal X-ray structures (Figure S5-S7, Table S2) S10

2.3 Theoretical calculations (Figures S8-S11, Tables S3-S25) S14

2.4. Photophysical study (Figures S12-S18, Tables S26, S27) S47

3. References S53

**1.-Experimental section.**

**1.1. General procedures and materials.**

IR spectra were recorded on a Perkin-Elmer Spectrum 100 FT-IR Spectrometer (ATR in the range 250-4000 cm^-1^). Mass spectral analyses were performed with a Microflex MALDI-TOF Bruker or an Autoflex III MALDI-TOF Bruker instruments. C, H, and N analyses were carried out in a Perkin-Elmer 2400 CHNS analyzer. ^1^H, ^13^C{^1^H}, ^195^Pt{^1^H} NMR spectra were recorded on Bruker Avance 300 and 400 MHz instrument using the standard references: SiMe_4_ for ^1^H and ^13^C and Na_2_PtCl_6_ in D_2_O for ^195^Pt. *J* is given in Hz and assignments are based on ^1^H-^1^H COSY,^1^H-^1^H NOESY, ^1^H-^13^C HSQC and HMBC experiments. UV-visible spectra were recorded on a Unicam UV4 spectrophotometer. Steady-state photoluminescence spectra were recorded on a Jobin-Yvon Horiba Fluorolog FL-3-11 Tau 3 spectrofluorimeter. Emission lifetimes were recorded with a Datastation HUB-B with a nanoLED controller and software DAS6. The nanoLEDs employed for lifetime measurements were of 390 nm. The lifetime data were fitted using the Jobin-Yvon software package and the Origin Pro 8 program. Quantum yields were measured using the Hamamatsu Absolute PL Quantum Yield Measurement System C11347-11. PMMA films were prepared by drop-casting solutions in Acetone (**1**) and CH_2_Cl_2_ (**2, 3** and **4**) (10^-2^ M) in the dark onto quartz slides and allowing the solvent to evaporate.

**1.2. Crystallographic data.**

Crystal data and other details of the structure analyses are presented in Table S1. Suitable crystals for X-ray diffraction studies were obtained by slow evaporation of a concentrated solution of **2** in DMSO or by slow diffusion of *n*-hexane into concentrated a solution of **3** in of CH_2_Cl_2_. Crystals were mounted at the end of a quartz fibre. The radiation used in all cases was graphite monochromated MoKα (λ = 0.71073 Å). X-ray intensity data were collected on an Oxford Diffraction Xcalibur diffractometer. The diffraction frames were integrated and corrected from absorption by using the CrysAlis RED program.^1^

The structures were solved by Patterson and Fourier methods and refined by full-matrix least squares on *F^2^* with SHELXL.^2^ All non-hydrogen atoms were assigned anisotropic displacement parameters. The positions of the hydrogen atoms were constrained to idealised geometries and assigned isotropic displacement parameters equal to 1.2 or 1.5 times the *U*iso values of their respective parent atoms. The structure of **2·0.97 DMSO**, shows the presence of several moieties of DMSO. The asymmetric unit of the cell contains three molecules of the dinuclear complex, and what seems to be a not very efficient packing. This causes the presence of several voids that are occupied by the DMSO molecules, in occasions a somewhat diffuse way. Of the four molecules of DMSO present in the asymmetric unit, three of them show some degree of disorder. For this reason, restrains in some of the interatomic distances and values of the anisotropic displacement parameters were applied. In the structure of **3**, one of the ethyl residues is disordered over two positions which were refined with 0.5 partial occupancy each. Full-matrix least-squares refinement of the models against *F*2 converged to final residual indices given in Table S1.

**Table S1.** Crystallographic data

|  | **2·0.97 DMSO** | **3** |
| --- | --- | --- |
| Empirical formula | C_35.93_H_41.80_N_8_O_4.97_Pt_2_S_0.97_ | C_36_H_40_N_8_O_4_Pt_2_ |
| Formula weight | 1086.41 | 1038.94 |
| Crystal system | Triclinic | Monoclinic |
| temperature | 100(2) K | 150(2) K |
| Space group | P -1 | P 21/n |
| a (Å) | 16.78130(19) | 15.3406(2) |
| b (Å) | 18.9598(2) | 16.90570(17) |
| c (Å) | 21.5242(3) | 15.4484(2) |
| α (°) | 104.0739(11) | 90 |
| β (°) | 106.6159(11) | 113.8209(18) |
| γ (°) | 108.0713(10) | 90 |
| Volume (Å3)/Z | 5809.95(13)/6 | 3665.15(10)/4 |
| ρ (Mg/m3) | 1.863 | 1.883 |
| μ (Mo-Kα)/mm-1 | 7.319 | 7.675 |
| F(000) | 3148 | 2000 |
| Crystal size (mm3) | 0.440 x 0.310 x 0.070 | 0.44 x 0.29 x 0.21 |
| Theta range (°) | 2.467 to 28.478 | 3.381 to 29.375 |
| Reflections collected | 179925 | 40869 |
| Independent reflections [R(int)] | 26615 [R(int) = 0.0430] | 9026 [R(int) = 0.0316] |
| Final R1, wR2 [I>2sigma(I)] | 0.0374,0.0895 | 0.0225, 0.0486 |
| R1,wR2 (all data) | 0.0475, 0.0947 | 0.0278, 0.0506 |
| GOF (F^2^) | 1.044 | 1.001 |
| Largest diff. peak and hole/e.Å^-3^ | 4.204 and -2.045 | 1.133 and -1.032 |

§ *wR*2 = [ Σ wΔ2 / Σ wFo4]0.5 ; S = [ Σ wΔ2 / (N - NV)]0.5 ; *R*1 = Σ ||Fo| - |Fc|| / Σ |Fo| ; Δ = Fo2 - Fc2; N = NO + restraints; w = [σc2(Fo2)+(gP)2]-1 , σc2(Fo2) = variance in Fo2 due to counting statistics, P = [max(Fo2,0)+2Fc2]/3.

**1.3.-Computational methods**

Density functional calculations were carried out on the ground (S_0_) and triplet (T_1_) states with the Gaussian 16^3^ suite of programs, using the M06 hybrid density functional^4^ (MUE (M06) = 2.48 kcal/mol^5^) together with Grimme’s D3 dispersion correction^6^. The ECP-60-mwb pseudopotential^7^ was used for platinum, and the 6-31G(d)^8, 9^ basis sets were used for all other atoms. In order to facilitate the theoretical study, we have done a simplification on the real system, we have modelled the ethanoate substituent on the cyclometalated ligand as an acetate. General geometry optimizations were performed without any symmetry restriction and in THF by using the polarizable continuum model (PCM)^10^. Frequency calculations were performed in order to determine the nature of the stationary points found in So and T1 (no imaginary frequencies for minima, only one imaginary frequency for **1-TS**, and two imaginary frequencies for **2-TS**). The time-dependent density-functional (TD-DFT) calculations were also carried out in THF solution. Mulliken population analysis was carried out as implemented in Gaussian 16 package^3^. ChemissianLab program package was used for analysis and graphic representation of molecular structures and orbitals and for Mayer Bond Order analysis. Atomic charges were calculated by using the NBO analysis option as incorporated in Gaussian 16. Atomic coordinates (x, y, z) for the optimized structures are listed in the Tables S5−S25.

The phosphorescence spectra were simulated on the basis of ΔSCF-DFT calculations, which yield the energy difference between the triplet excited states at their optimized geometry and the closedshell ground state at the same geometry in THF (PCM).

**2.- Results and discussion**

**2.1.-NMR figures for characterization.**


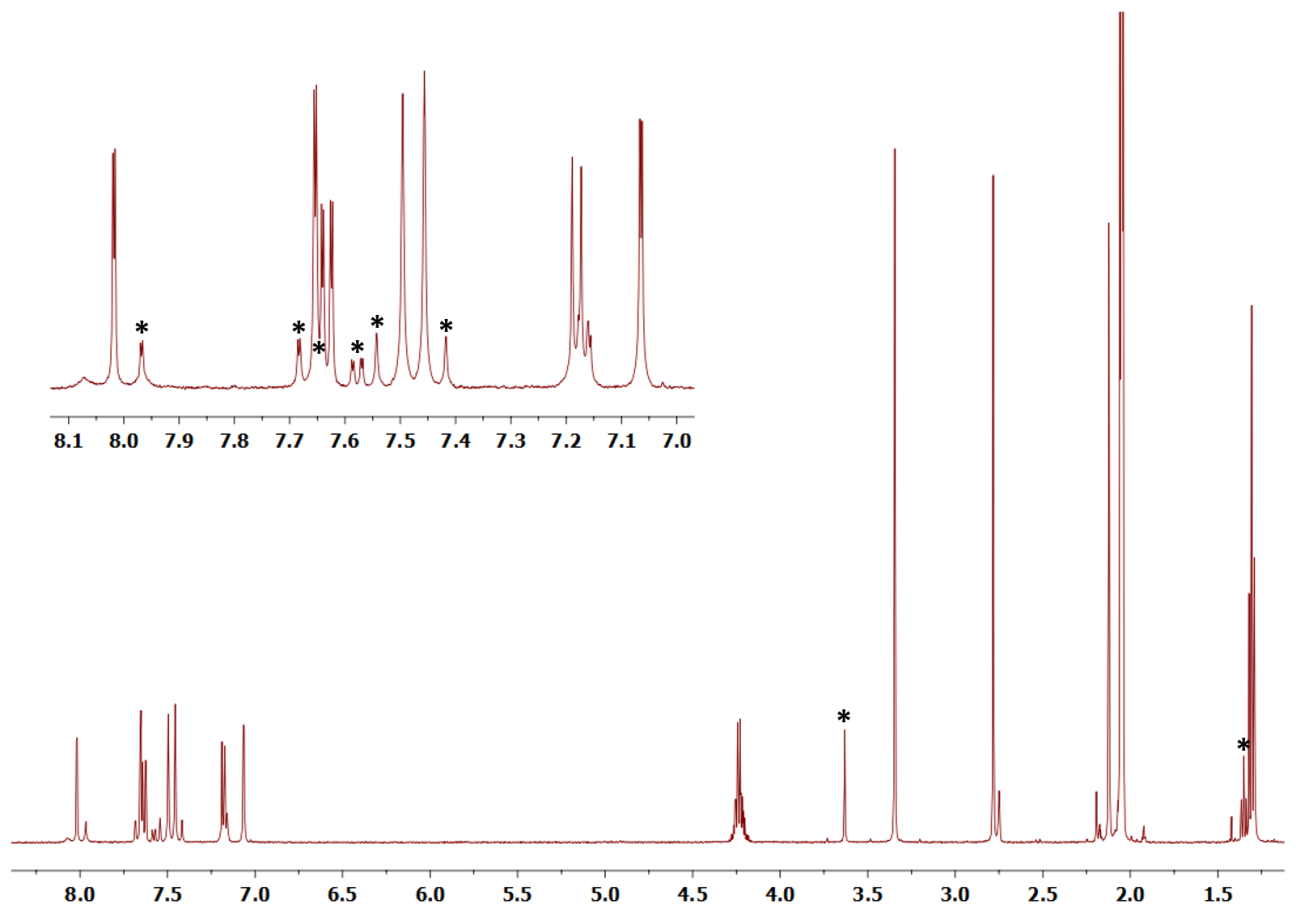


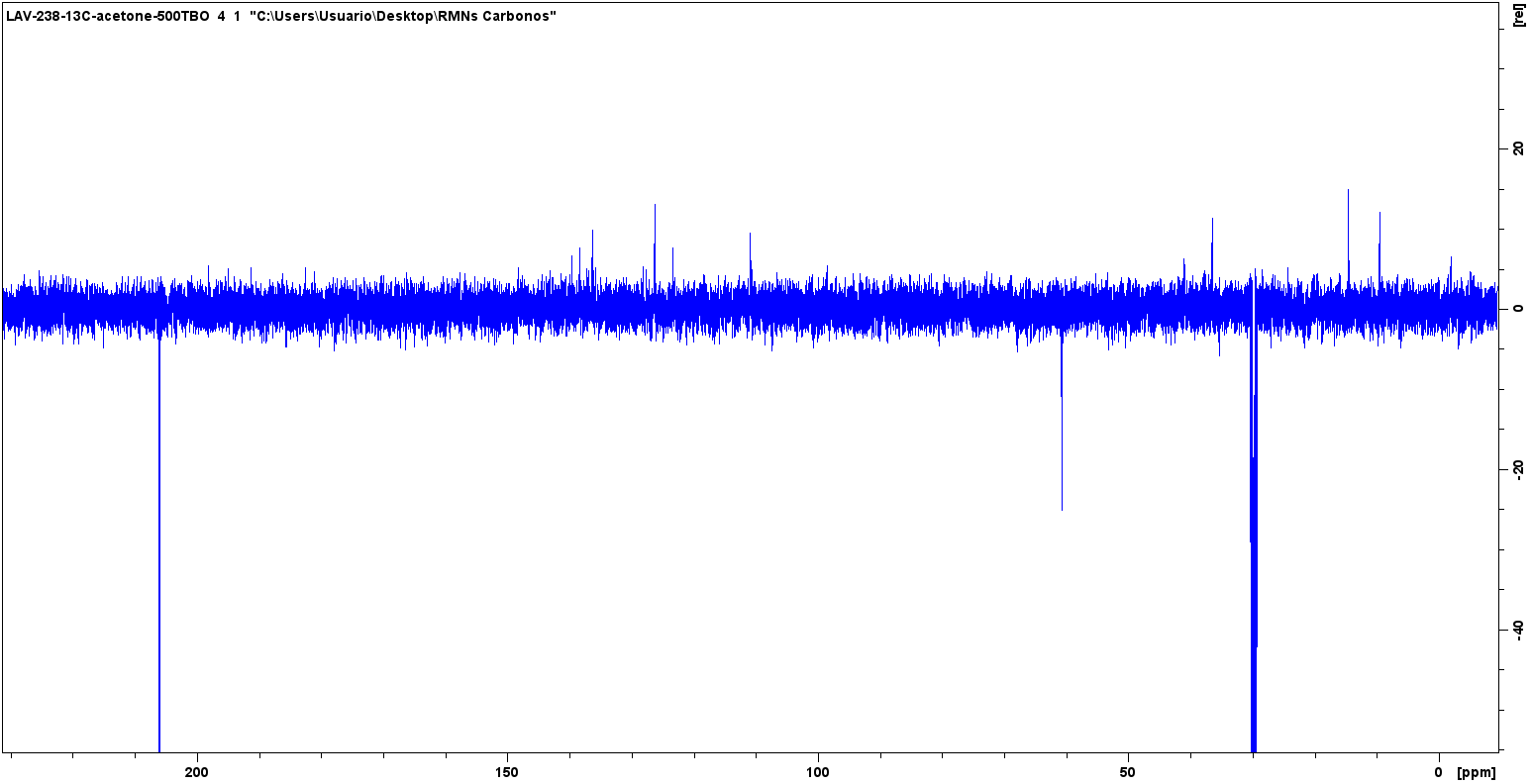


**Figure S1.** Top:  ^1^H NMR spectrum of ***syn-/anti-* [{Pt(EtO_2_C-C^C*)(μ-4-Mepz)}_2_] (2)** in acetone-*d*_6_. *is used to label signals corresponding to the syn species. Buttom: ^13^C{^1^H} APT NMR spectrum of **2** in acetone-*d*_6_


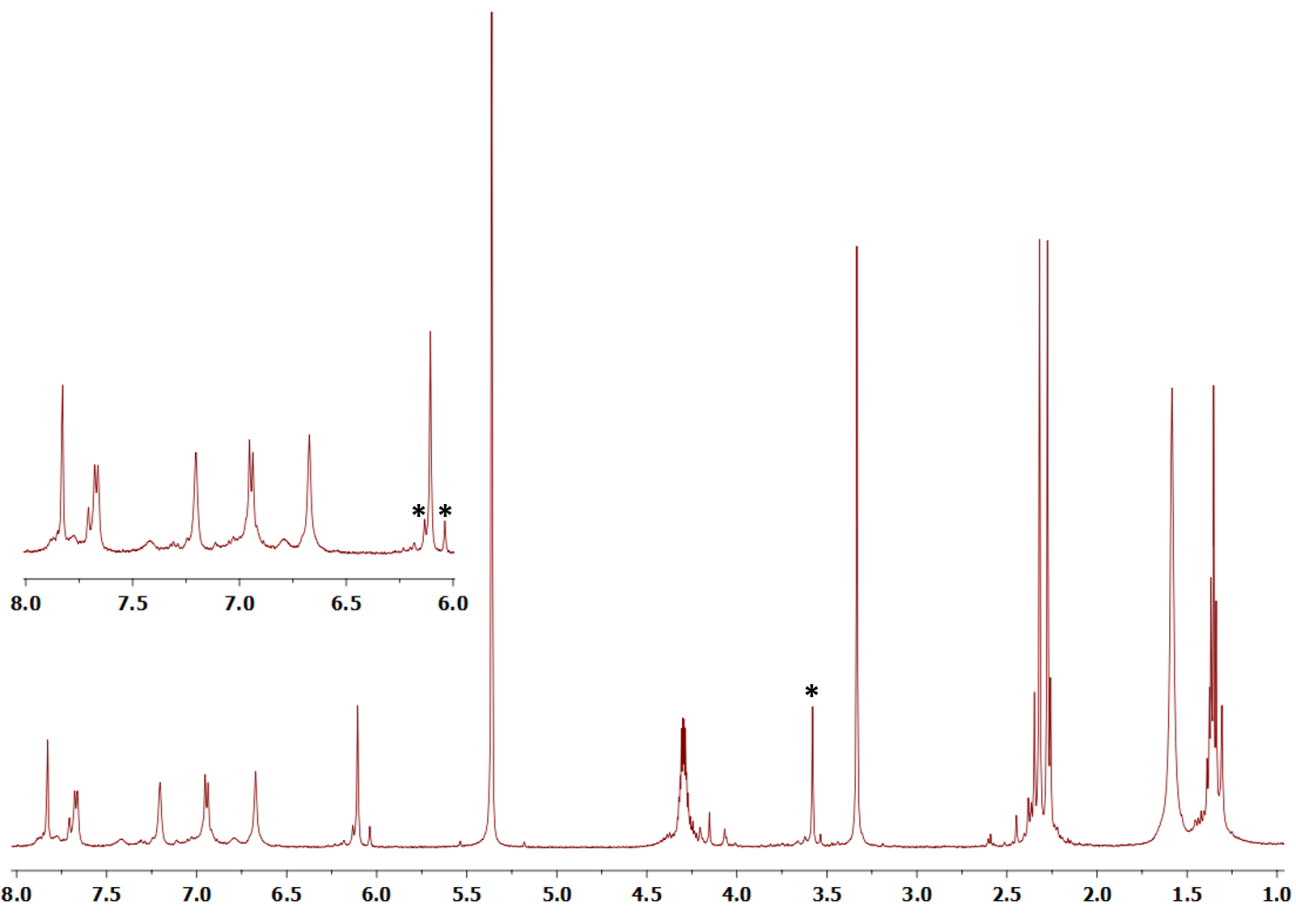


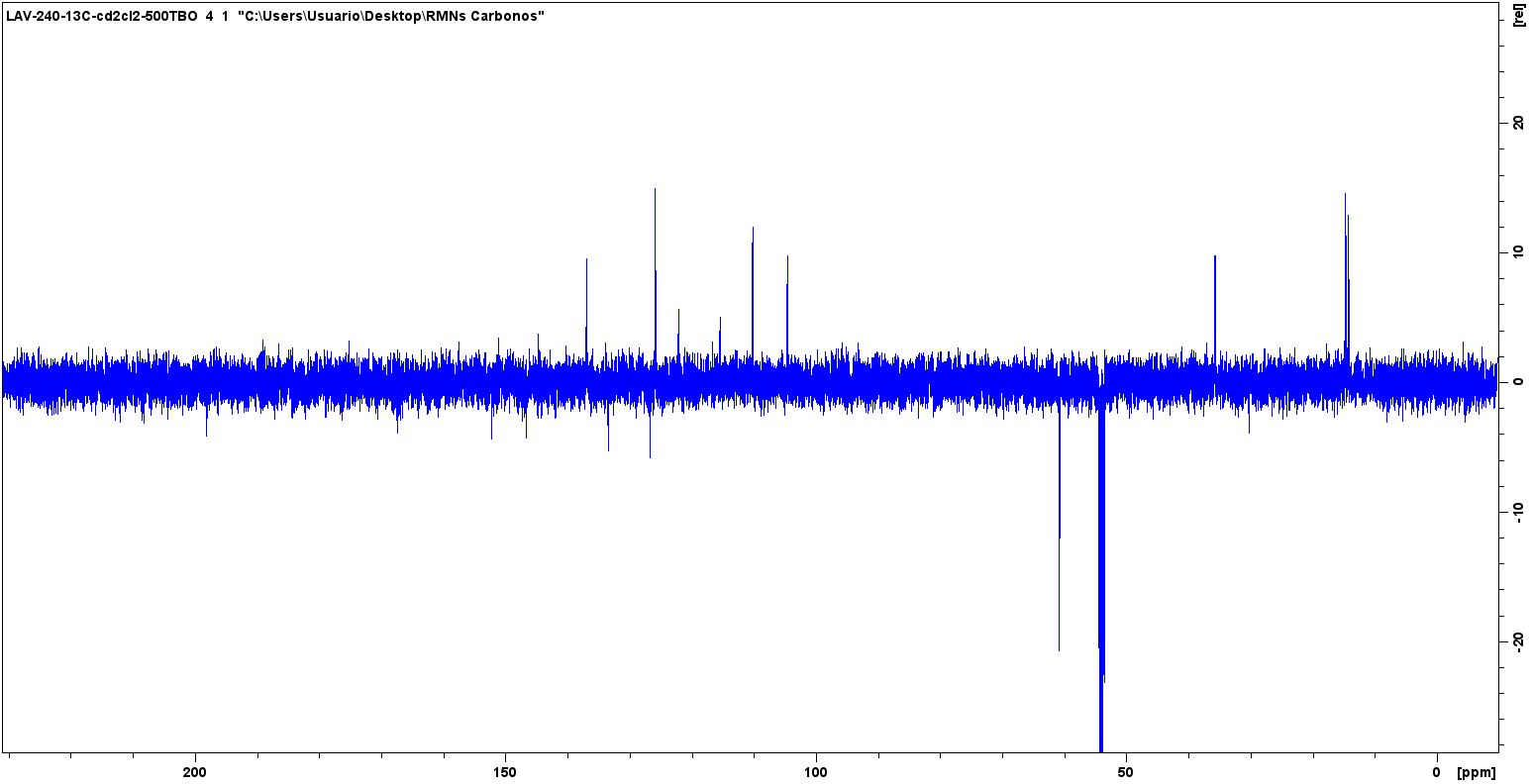


**Figure S2.** Top:  ^1^H NMR spectrum of ***syn-/anti-* [{Pt(EtO_2_C-C^C*)(μ-3,5-dmpz)}_2_] (3)** in CD_2_Cl_2_. *is used to label signals corresponding to the syn species. Buttom: ^13^C{^1^H} APT NMR spectrum of **3** in CD_2_Cl_2_


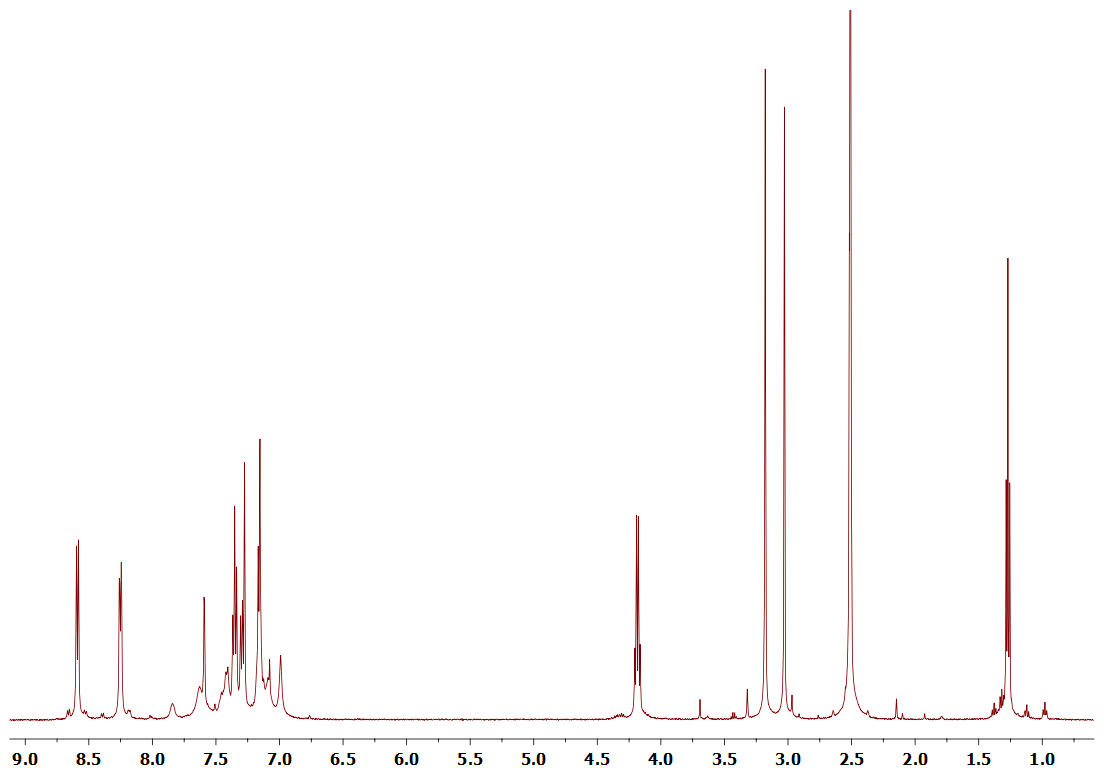


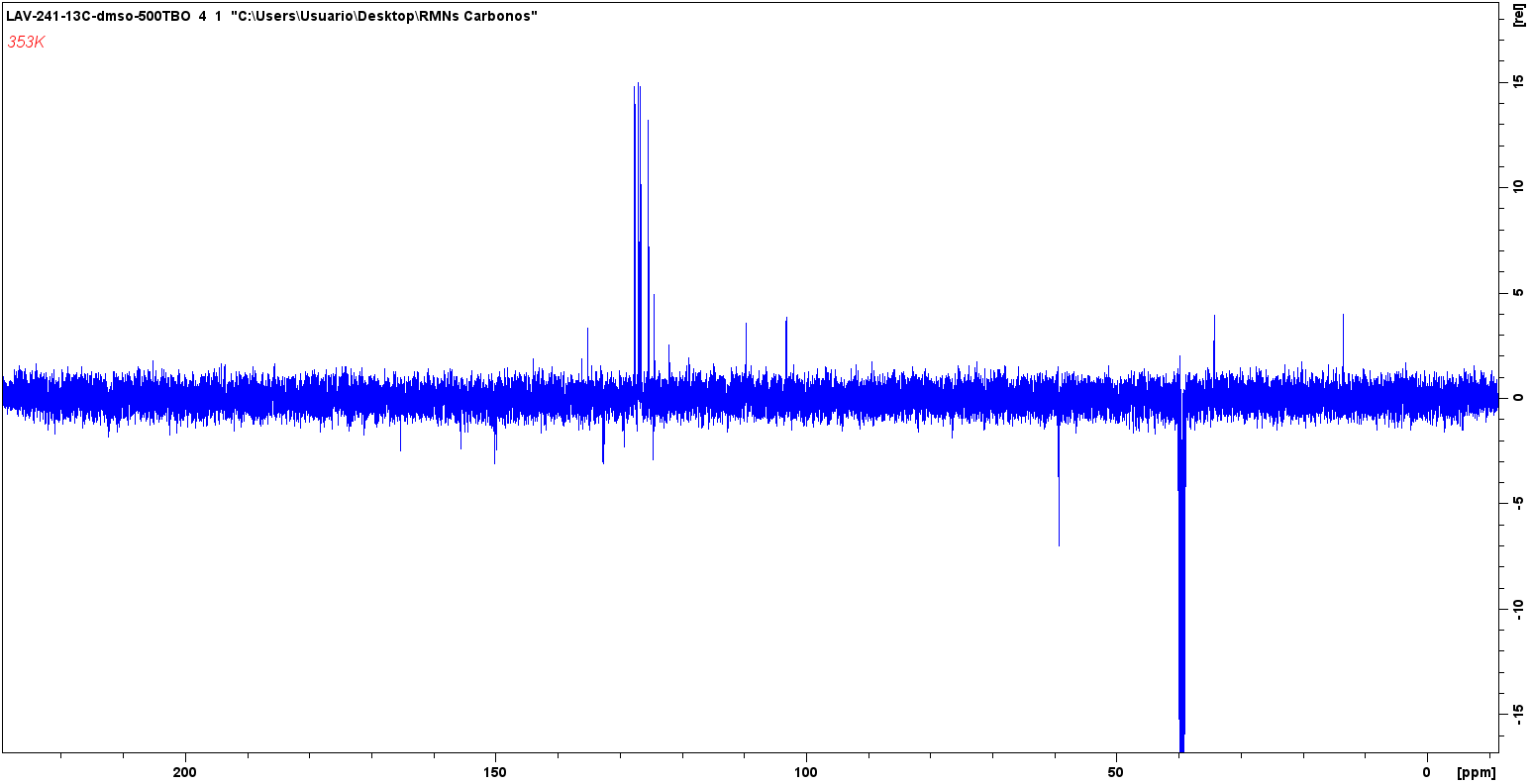


**Figure S3.** Top:  ^1^H NMR spectrum of **[{Pt(EtO_2_C-C^C*)(μ-3,5- dppz)}_2_] (4)** in DMSO-*d*_6_ at 353K. Buttom: ^13^C{^1^H} APT NMR spectrum of **4** in DMSO-d_6_ at 353K


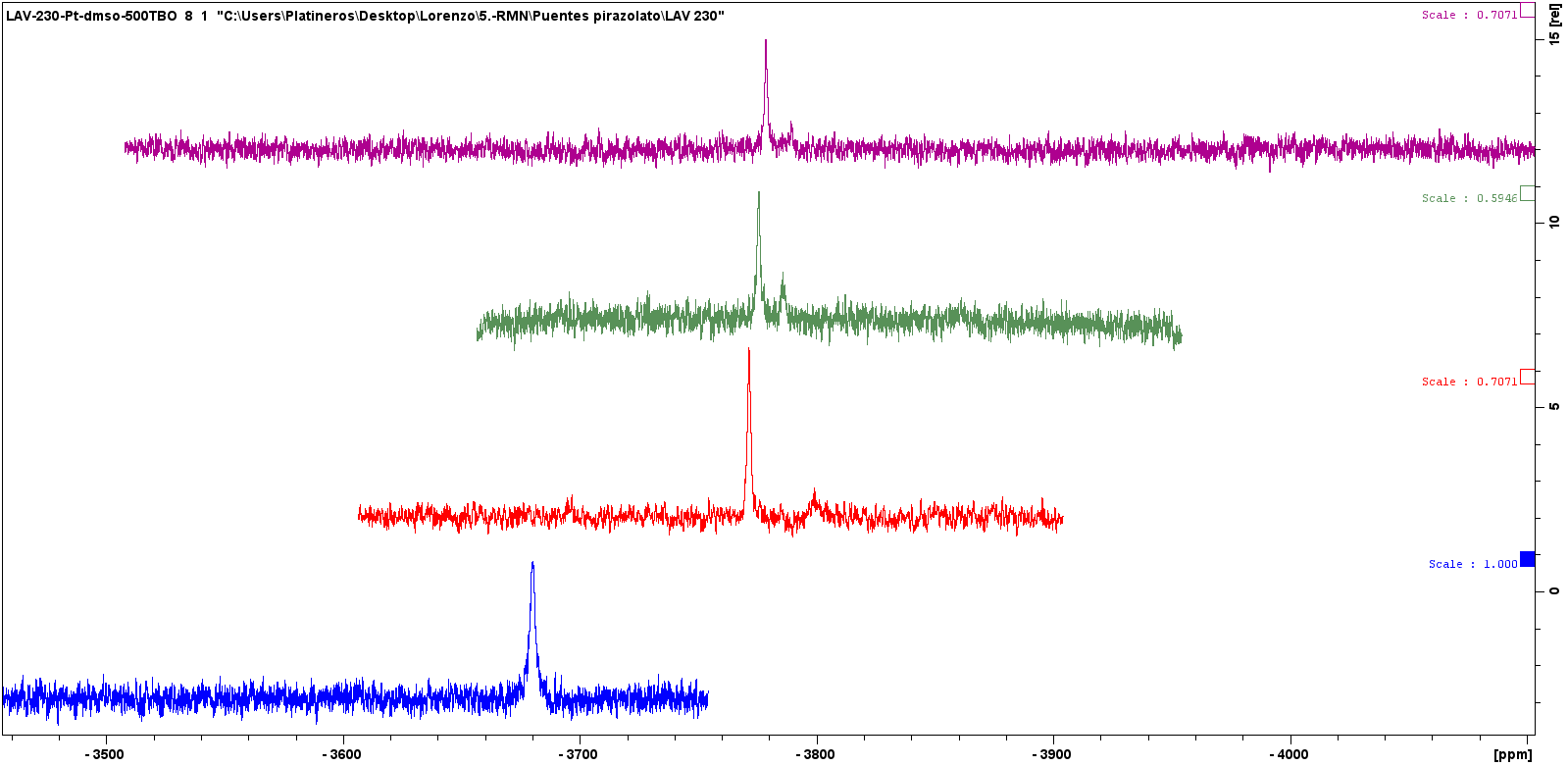


**Figure S4.** ^195^Pt{^1^H} NMR spectra of *syn-/anti-* [{Pt(EtO_2_C-C^C*)(μ-pz)}_2_] (**1**) in acetone-*d*_6_ (purple), *syn-/anti-* [{Pt(EtO_2_C-C^C*)(μ-4-Mepz)}_2_] (**2**) in acetone-*d*_6_ (green), *syn-/anti-* [{Pt(EtO_2_C-C^C*)(μ-3,5-dmpz)}_2_] (**3**) in CD_2_Cl_2_ (red), anti- [{Pt(EtO_2_C-C^C*)(μ-3,5- dppz)}_2_] (**4**) in DMSO-*d*_6_ at 353K (blue).

**2.2 Single-crystal X-ray structures**

Compound **2** shows three butterfly-like molecules (**A**, **B**, **C**) in the asymmetric unit, alike complex **1**,^11^ with intermetallic separations of 3.3555(4) Å (**2A**), 3.2242(3) Å (**2B**) and 3.1560(3) Å (**2C**) while compound **3** shows only one dinuclear molecule with an intermetallic distance of 3.13100(17) Å (see Table S2 and Figure S5). Molecules in both compounds, **2** and **3**, exhibit a dinuclear structure containing two “Pt(EtO_2_C-C^C*)” metalocycles bridged by two Rpz ligands and displaying an *anti* arrangement of the Pt-C* bonds. The six-membered ring Pt_2_N_4_ has the typical boat-like conformation with an angle between the Pt-N-N-Pt fragments of about 80ᵒ [78.67(13)ᵒ (**2A**), 80.43(10)ᵒ (**2B**), 82.68(17)ᵒ (**2C**)] and 85.30(6)ᵒ (**3**)] and an angle between the best least-squares planes of the platinum environments of 81.84(16)ᵒ (**2A**), 74.81(14)ᵒ (**2B**), 69.62(14)ᵒ (**2C**) and 68.77(6)ᵒ (**3**). The angle between the Pt-Pt line and both metal coordination planes are within the range of 33ᵒ to 42ᵒ *aprox*. [39.82(10)ᵒ Pt(1), 42.23(10)ᵒ Pt(2) for **2A**; 38.73(11)ᵒ Pt(1), 36.36(09)ᵒ Pt(2) for **2B**; 33.50(10)ᵒ Pt(1), 36.12(09)ᵒ Pt(2) for **2C**; 35.10(5)ᵒ Pt(1); 33.69(4)ᵒ Pt(2) for **3**]. In the dinuclear molecules, each Pt center lies in a distorted square planar coordination environment as a consequence of the small bite angle of the EtO_2_C-C^C* cyclometalated ligand of about 80ᵒ (Table S2). These angles together with the Pt–C_Ar_ and Pt–C* distances are similar to those found in other compounds containing five-membered cycloplatinated N-heterocyclic carbenes.^11-14^ The Pt-N bond distances are also similar to those found in dinuclear bis-pyrazolate-bridge complexes containing ligands with high *trans* influence.^15^

*
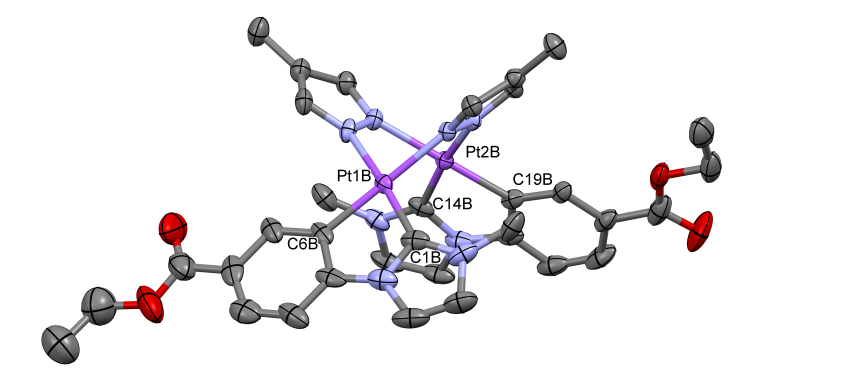

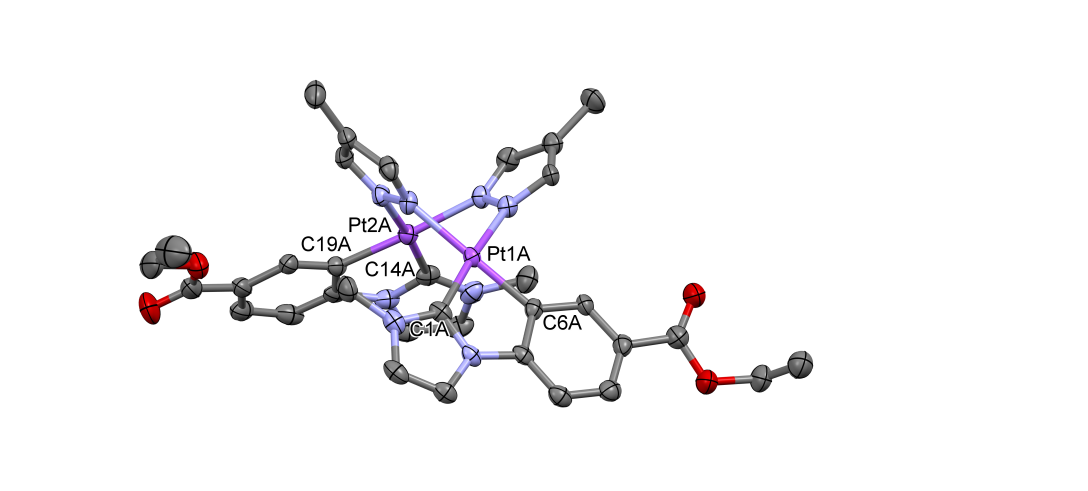
*

1. *
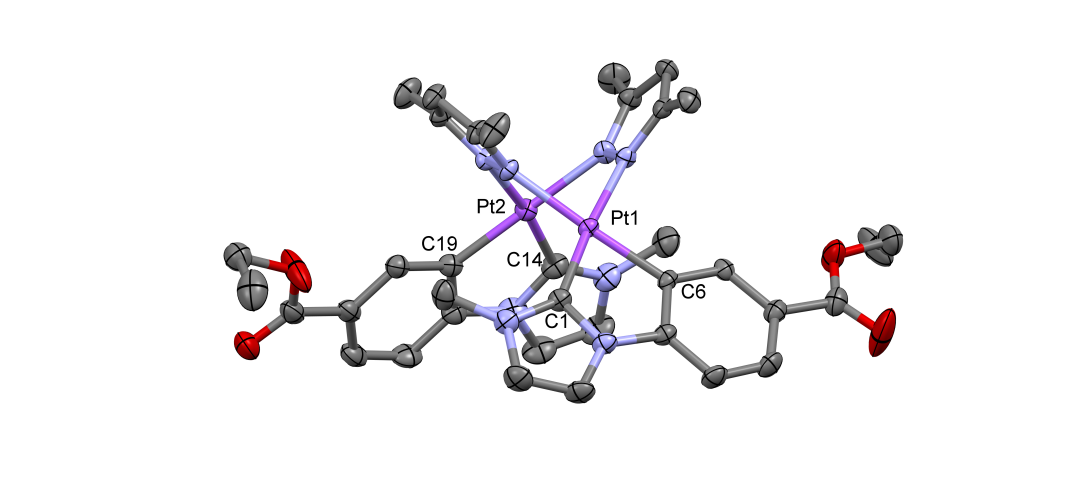

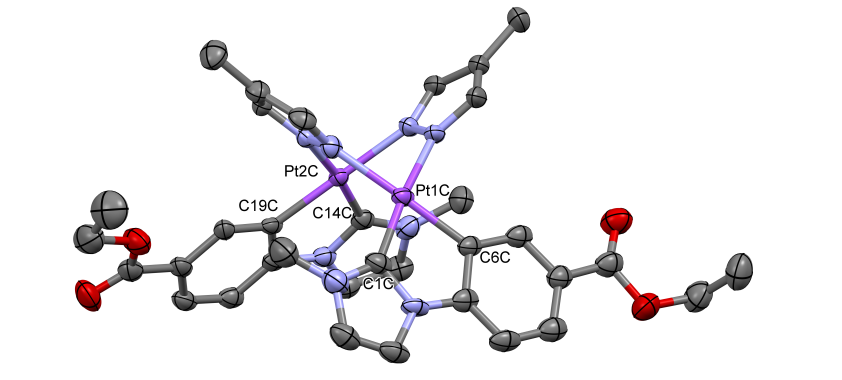
* (b)
2. (d)

**Figure S5**. Molecular structures of **2A** (a), **2B** (b), **2C** (c) and **3** (d) Ellipsoids are drawn at their 50% probability level; solvent molecules and hydrogen atoms have been omitted for clarity.

In complex **3**, the Pt···Pt separation (3.131(17) Å) is a little shorter than the observed in the cyclometalated compound [Pt(E^N)(μ-3,5-dmpz)}_2_] (E^N = 2-(2,4-difluorophenyl)pyridyl,κ-C,N, d_Pt-Pt_ = 3.1914(9)Å,^15^ E^N = 2,2’-bipyrimidine,κ-N,N d_Pt-Pt_ = 3.1968(7)Å;^16^ E^N = 2-phenylpiridinate,κ-C,N d_Pt-Pt_ = 3.1904(13), 3.2029(13)Å^17^). It is big enough to ensure the absence of a metal-metal bond,^18-20^ but not to preclude some degree of intermetallic interaction.^21, 22^

**Table S2**: Selected bond lengths (Å) and angles (ᵒ) for **2·0.97 DMSO** and **3**.

|  | 2A | 2B | 2C | 3 |
| --- | --- | --- | --- | --- |
| Pt1-Pt2 | 3.3555(4) | 3.2242(3) | 3.1560(3) | 3.13100(17) |
| Pt1-C1 | 1.975(5) | 1.977(7) | 1.968(6) | 1.966(3) |
| Pt1-C6 | 2.022(6) | 2.012(6) | 2.015(7) | 2.009(3) |
| Pt1-N5 | 2.055(5) | 2.059(5) | 2.066(5) | 2.047(2) |
| Pt1-N7 | 2.093(5) | 2.085(4) | 2.098(5) | 2.086(2) |
| Pt2-C14 | 1.954(6) | 1.975(6) | 1.964(6) | 1.967(3) |
| Pt2-C19 | 2.014(6) | 2.017(6) | 2.010(6) | 1.995(3) |
| Pt2-N8 | 2.052(5) | 2.061(5) | 2.057(5) | 2.048(2) |
| Pt2-N6 | 2.095(5) | 2.092(5) | 2.100(5) | 2.100(2) |
| C1-Pt1-C6 | 79.9(2) | 79.7(3) | 80.6(3) | 80.05(12) |
| C1-Pt1-N7 | 99.6(2) | 97.7(2) | 100.9(3) | 102.47(11) |
| C6-Pt1-N5 | 95.7(2) | 96.3(3) | 93.1(2) | 94.72(11) |
| N7-Pt1-N5 | 85.02(19) | 86.47(18) | 85.2(2) | 82.76(9) |
| C14-Pt2-C19 | 80.3(3) | 80.1(3) | 80.1(3) | 80.22(13) |
| C14-Pt2-N6 | 99.0(2) | 99.7(2) | 101.1(2) | 103.56(12) |
| C19-Pt2-N8 | 95.1(2) | 94.7(2) | 93.7(2) | 92.25(11) |
| N8-Pt2-N6 | 85.71(18) | 85.57(18) | 85.12(19) | 83.87(10) |

These complexes, including compound **1**, show supramolecular arrangements due to short π-π contacts between the C^C* cyclometalated ligands (Figure S6) with C-C distances between 3.28 Å and 3.40 Å. In compound **3**, the π-π interactions take place on the internal face of the “C^C*” moiety bringing together only two molecules (dist C-C: 3.398 Å, Figure S6(c)). However, in compounds **1** and **2**, the π-π interactions are more numerous and take place on both, external and internal, faces of the molecules within the asymmetric unit giving rise to a much more extended network (dist C-C: 3.295 to 3.395 Å (**1**); 3.280 to 3.400 Å (**2**) Figures S6 (a, b))**.**

Unfortunately, no good quality crystals were obtained for **4**, but we could confirm the atom connectivity. Two different molecules with Pt-Pt separation of 3.054 and 2.982 Å were found in the asymmetric unit, as can be seen in Figure S7.

**
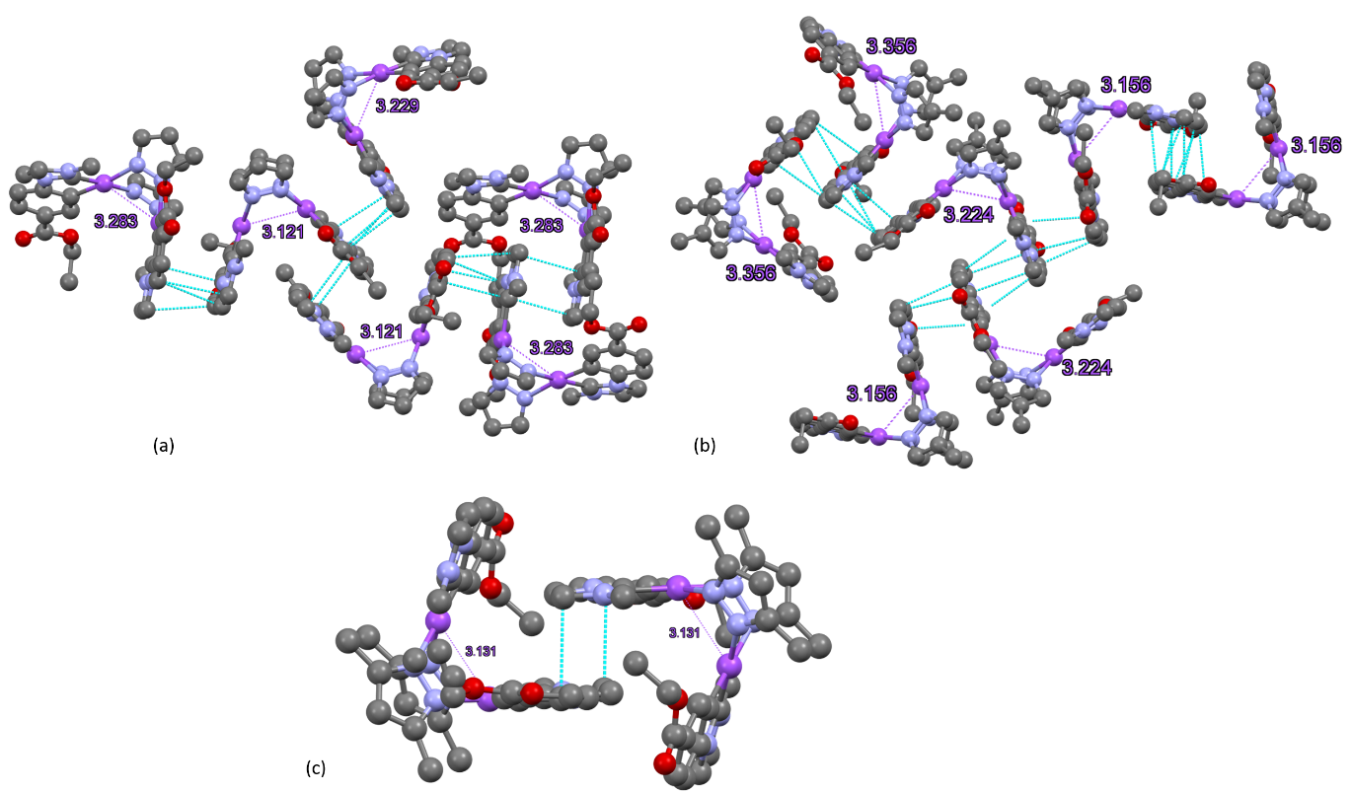
**

**Figure S6**. Supramolecular arrangement of complex **1** (a), **2** (b) and **3** (c).


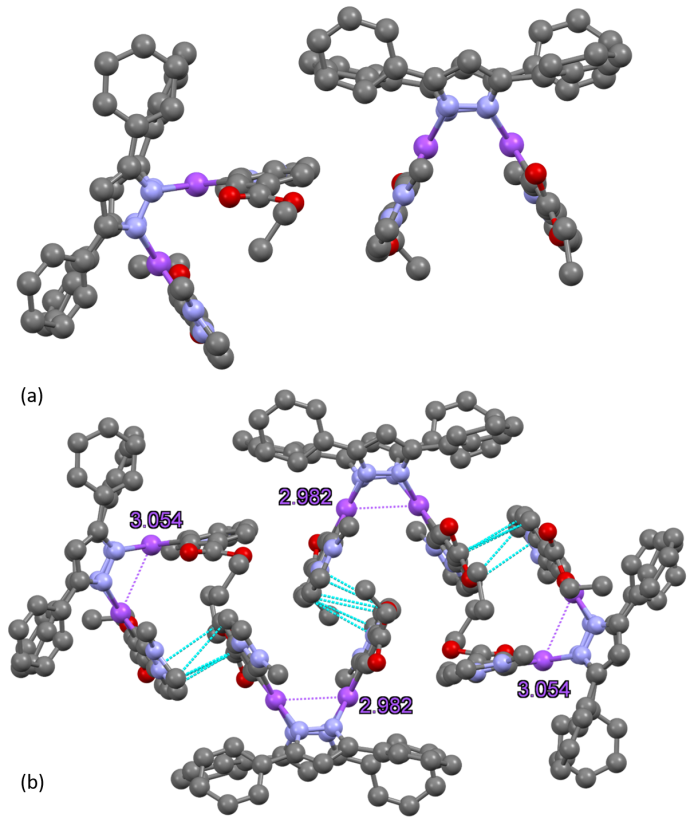


**Figure S7**. Complex **4**: Molecular structure (a) and supramolecular arrangement (b)

**2.3.-** **Theoretical calculations.**


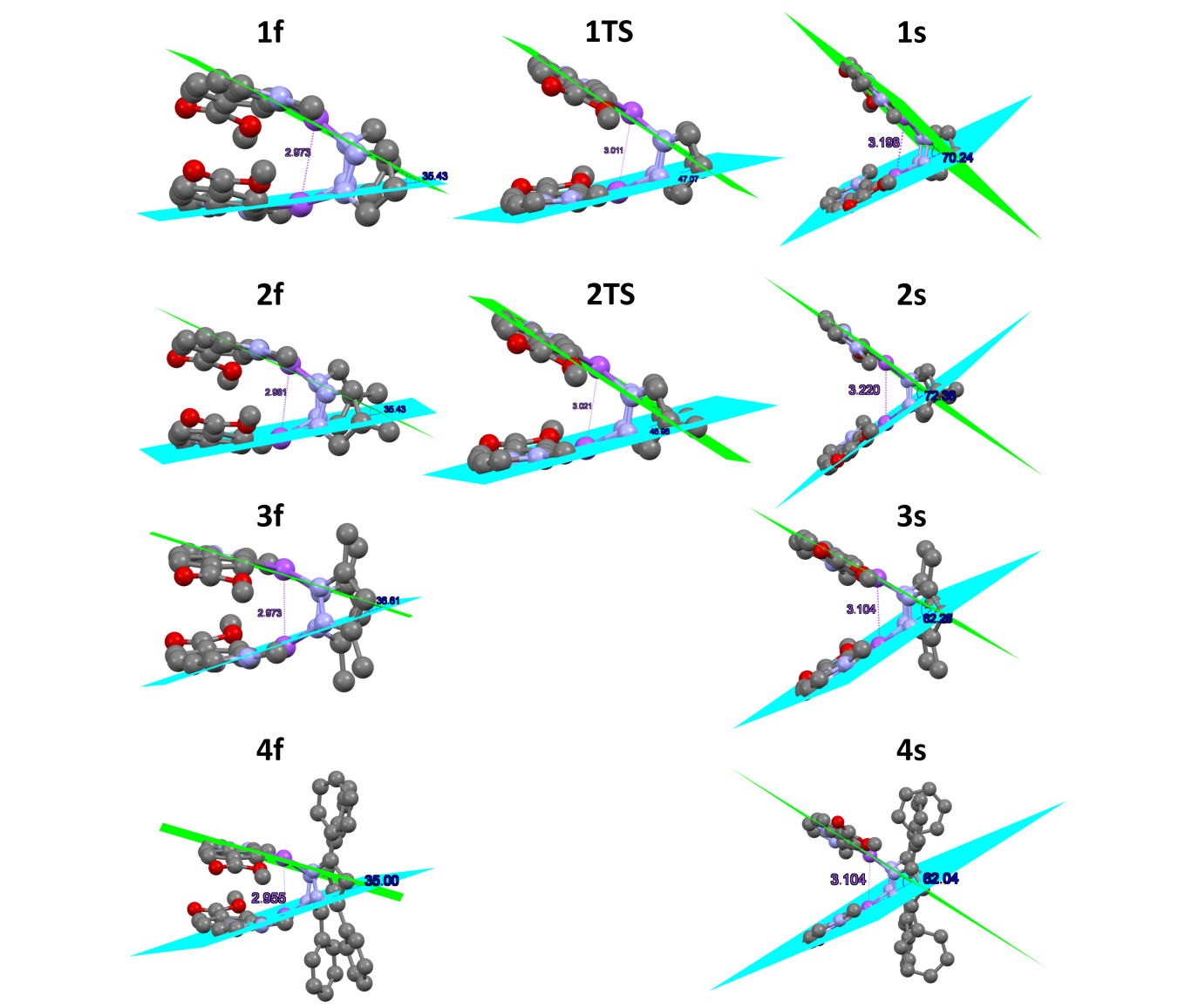


**Figure S8**. Optimized TS (center) and GS structures of the two conformers: the butterfly-folded (1f-4f, left) and the butterfly-spread ones (1s-4s, right)


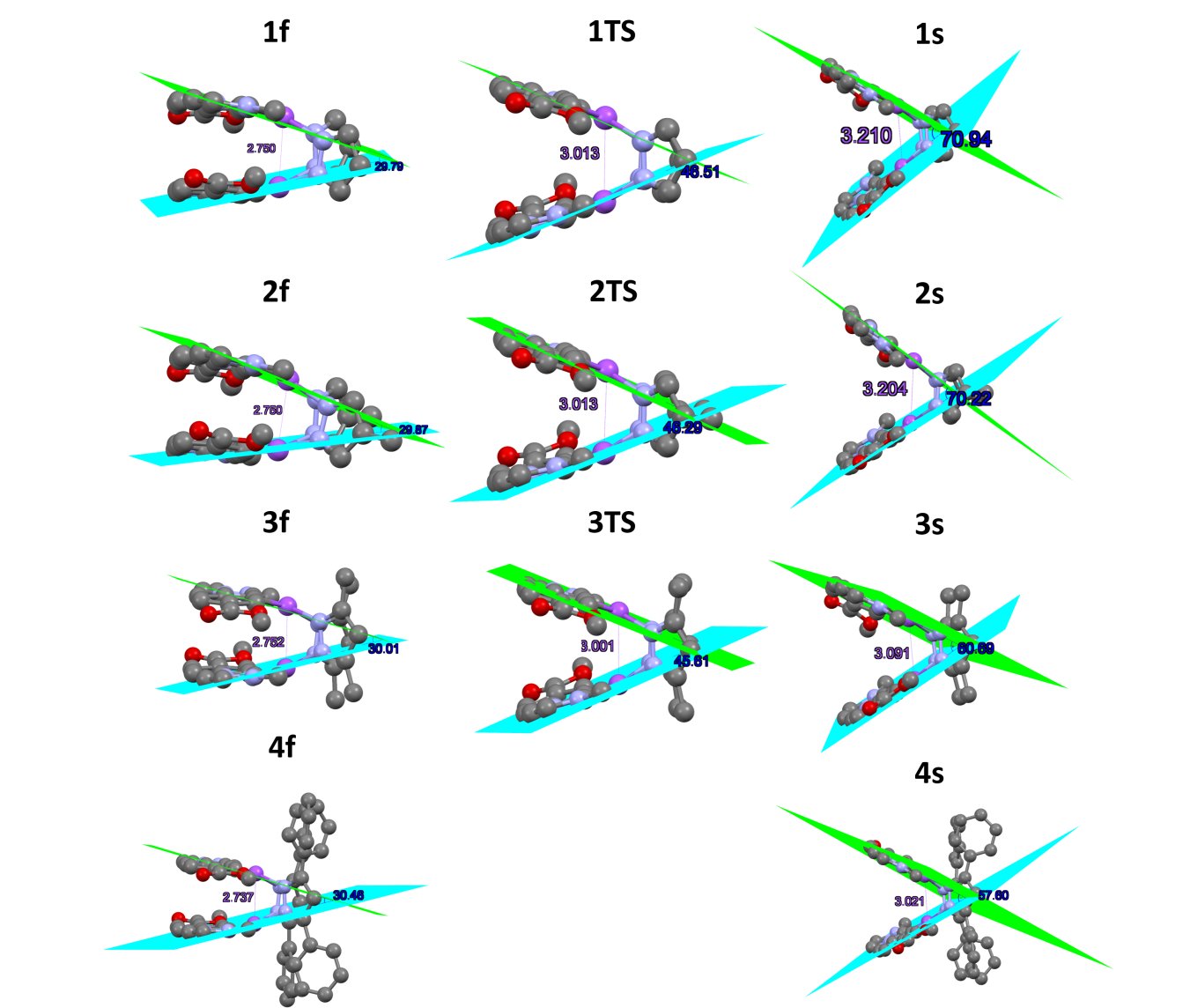


**Figure S9**. Optimized TS (center) and T_1_ structures of the two conformers: the butterfly-folded (1f-4f, left) and the butterfly-spread ones (1s-4s, right)


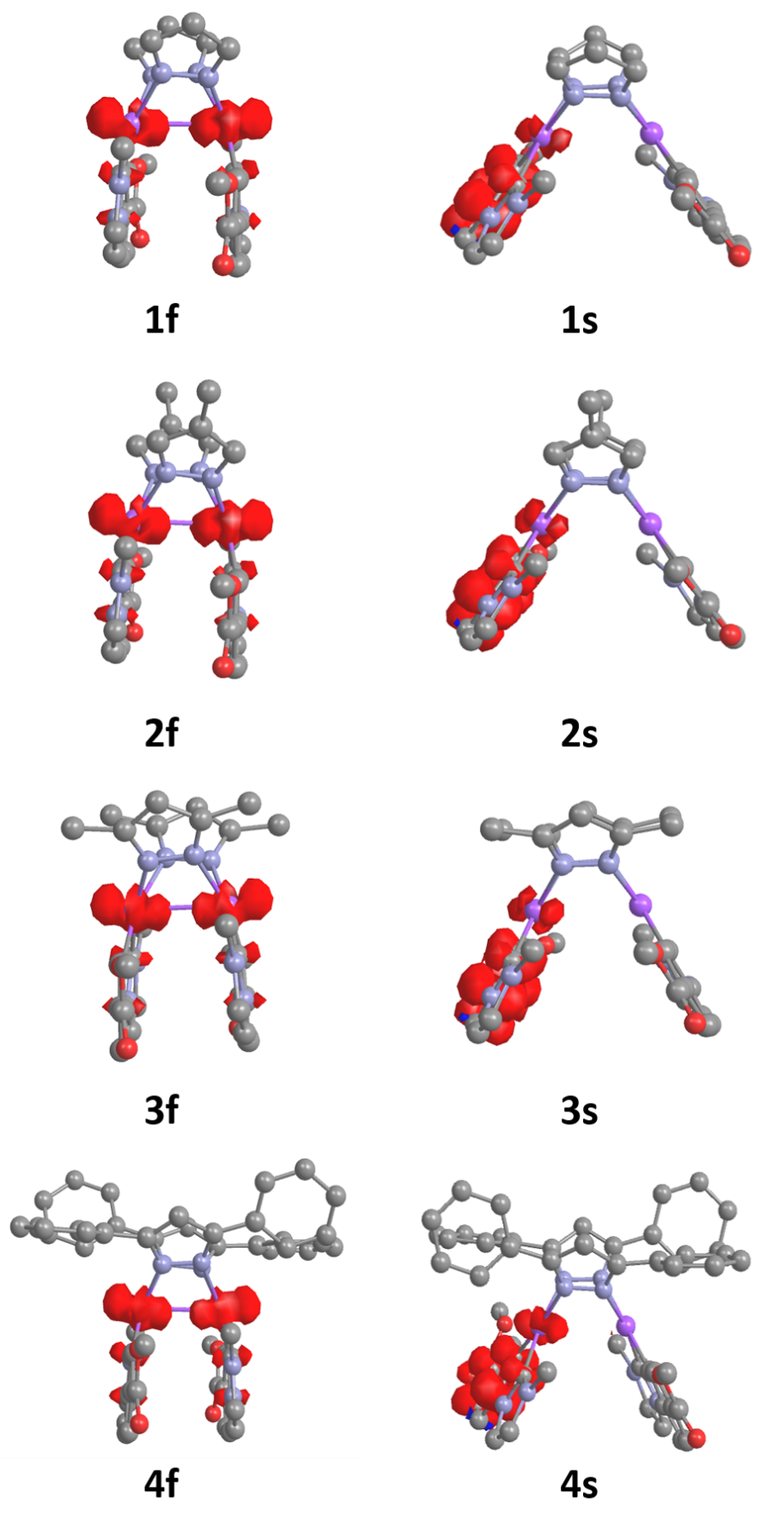


**Figure S10:** Spin density distribution in the first Triplet state (T_1_) for butterfly-folded (left) and butterfly-spread (right) conformers in solution of THF.


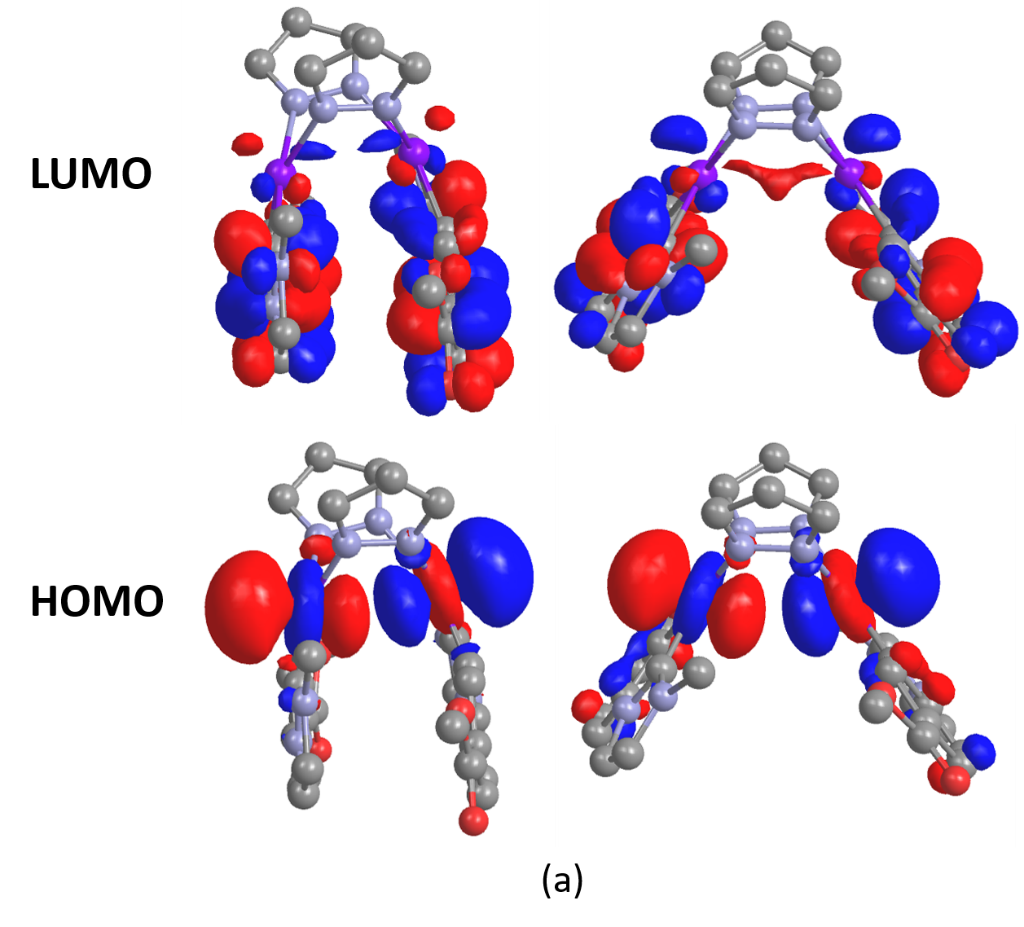

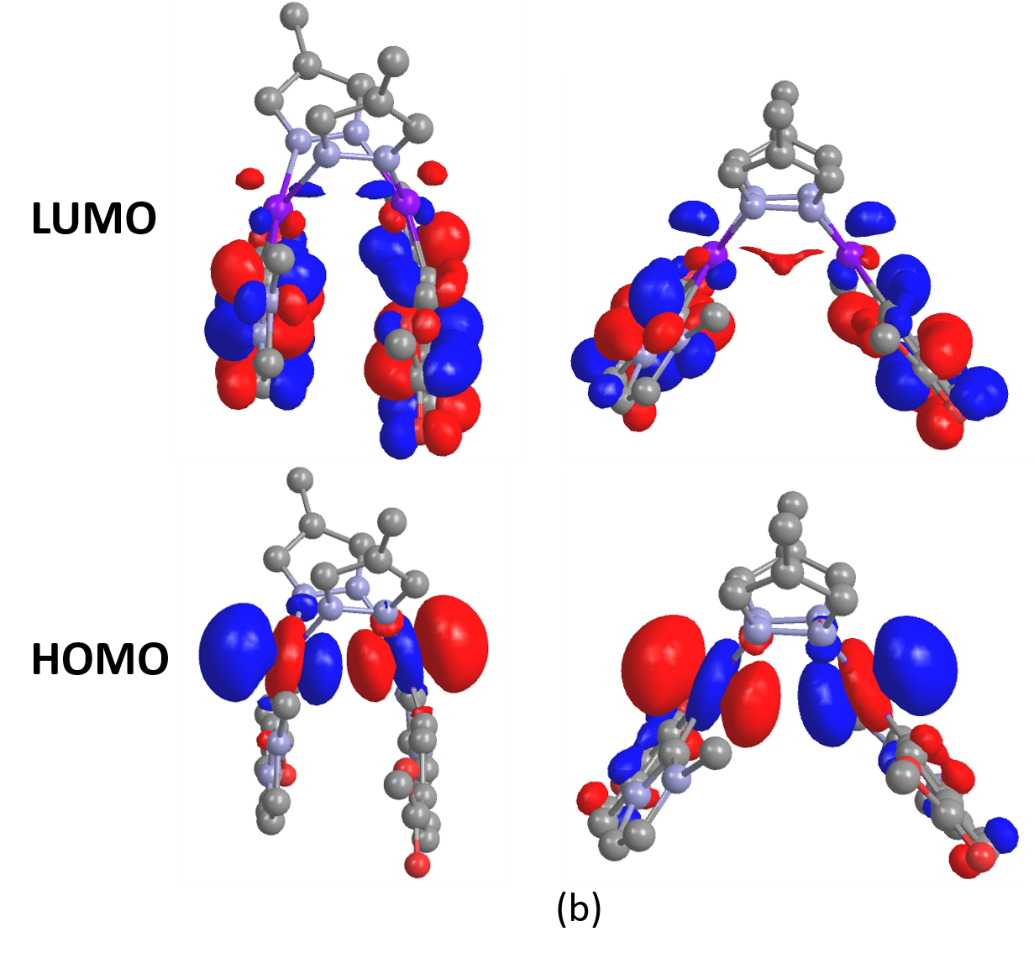


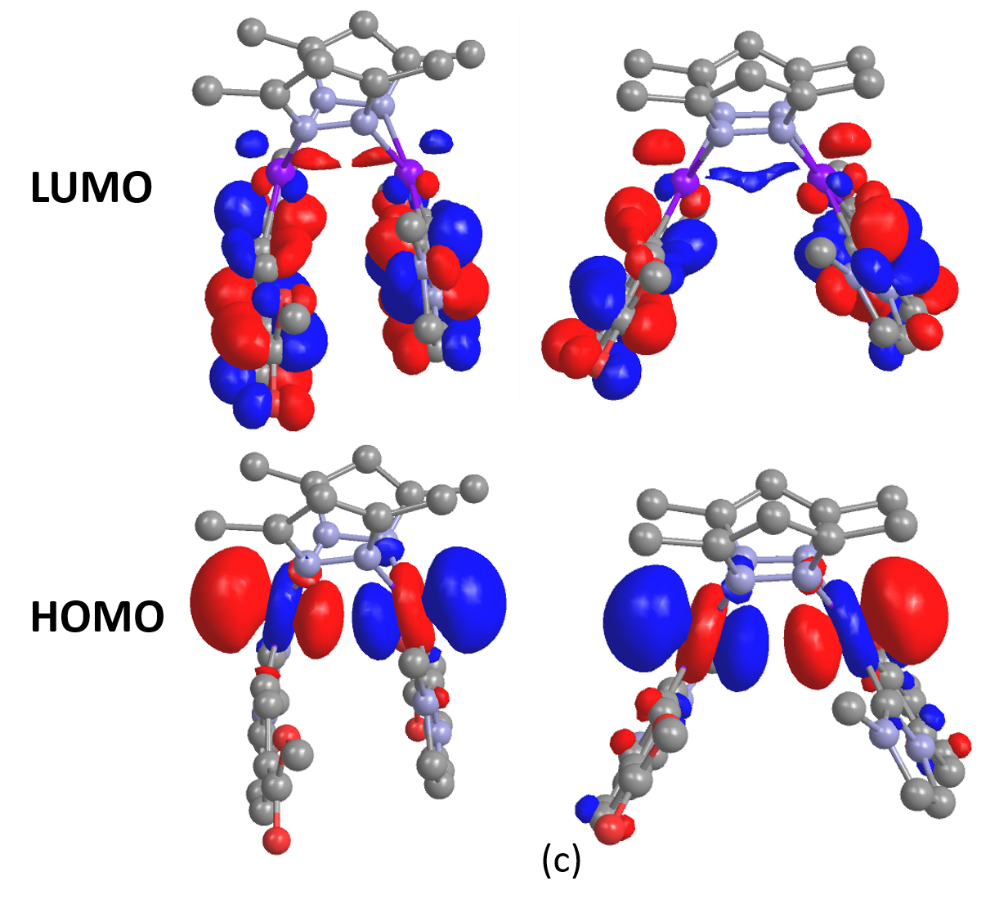


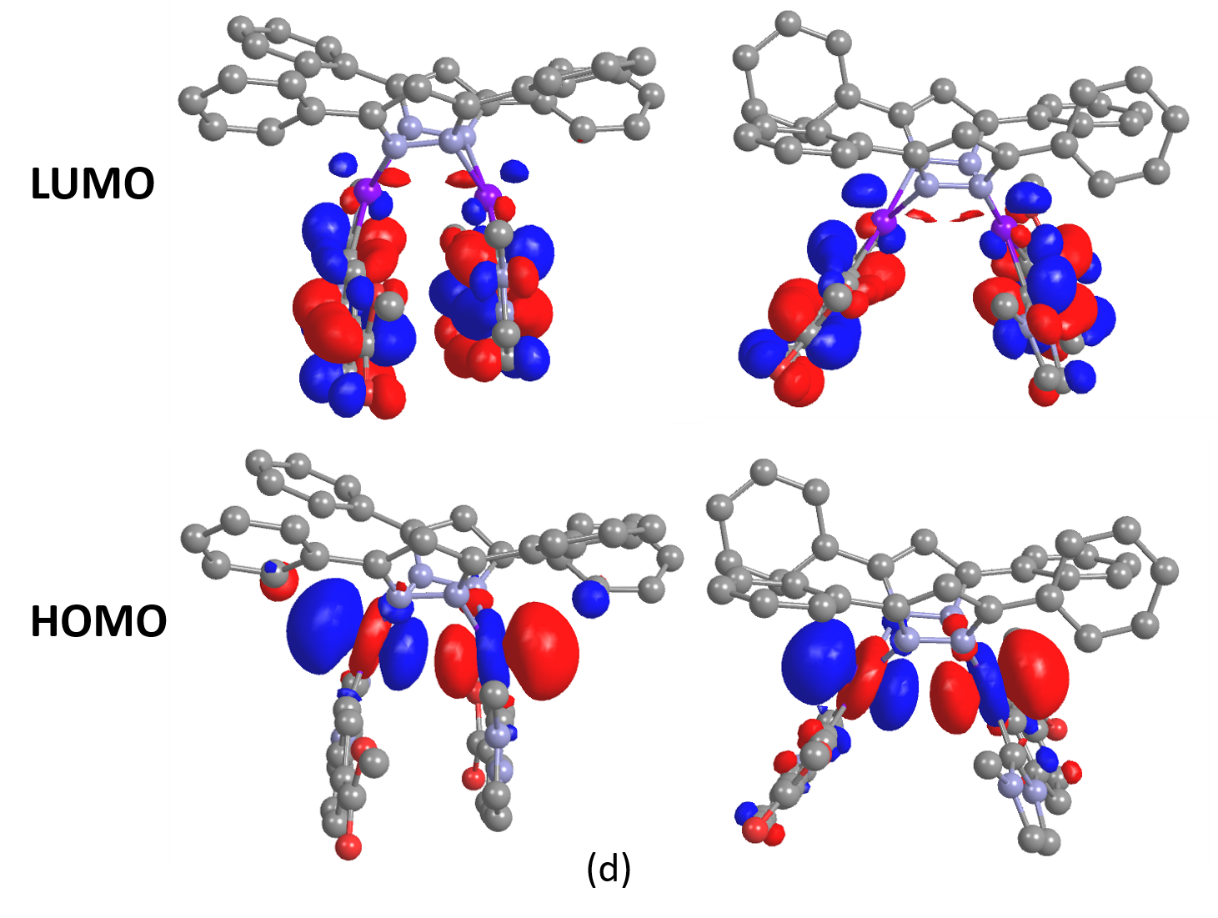


**Figure S11**: Calculated Frontier Molecular Orbitals (Isovalue =0.03) for the butterfly- folded (left) and butterfly-spread (right) conformers of **1** (a), **2**(b), **3**(c) and **4**(d).

**Table S3.** Population Analysis (%) of Frontier MOs in the S_0_ in THF solution for **1**-**4**.

| **Comp.** | **eV** | | **Pt** | | **C^C*** | | **Rpz** | |
| --- | --- | --- | --- | --- | --- | --- | --- | --- |
|  | **HOMO** | **LUMO** | **HOMO** | **LUMO** | **HOMO** | **LUMO** | **HOMO** | **LUMO** |
| **1f** | -5.45 | -1.58 | 90 | 20 | 7 | 77 | 3 | 3 |
| **1s** | -5.80 | -1.48 | 86 | 20 | 10 | 77 | 4 | 3 |
| **2f** | -5.42 | -1.57 | 90 | 19 | 6 | 79 | 4 | 2 |
| **2s** | -5.79 | -1.46 | 86 | 20 | 10 | 78 | 4 | 2 |
| **3f** | -5.39 | -1.56 | 90 | 22 | 6 | 75 | 4 | 3 |
| **3s** | -5.60 | -1.44 | 88 | 21 | 8 | 75 | 4 | 4 |
| **4f** | -5.40 | -1.56 | 86 | 22 | 6 | 74 | 8 | 5 |
| **4s** | -5.54 | -1.40 | 86 | 18 | 7 | 77 | 7 | 5 |

**Table S4.** S_1_ and T_1_ states calculated for **1**-**4** in THF.

|  | S_1_ | | | | T_1_ | |
| --- | --- | --- | --- | --- | --- | --- |
| **Comp.** | λexc (nm) | o.s. | Transition (%) | Assignment | λem /nm (eV) | Assignment |
| **1f** | 426 | 0.0801 | H→L 98% | IL/MLCT/MMLCT | 572 (2.168) | MMLCT |
| **1s** | 370 | 0.1392 | H→L 95% | IL/MLCT | 517 (2.397) | IL/MLCT |
| **2f** | 428 | 0.0764 | H→L 98% | IL/MLCT/MMLCT | 574 (2.158) | MMLCT |
| **2s** | 370 | 0.1313 | H→L 95% | IL/MLCT | 517 (2.396) | IL/MLCT |
| **3f** | 430 | 0.0775 | H→L 98% | IL/MLCT/MMLCT | 571 (2.171) | MMLCT |
| **3s** | 386 | 0.1322 | H→L 96% | IL/MLCT | 518 (2.397) | IL/MLCT |
| **4f** | 429 | 0.0601 | H→L 97% | IL/MLCT/MMLCT | 570 (2.175) | MMLCT |
| **4s** | 389 | 0.1161 | H→L 96% | IL/MLCT | 509 (2.436) | IL/MLCT |

**Table S5:** DFT-Optimized coordinates of **1-f** in the Ground State in THF

Center Coordinates (Angstroms) Center Coordinates (Angstroms)

X Y Z X Y Z

Pt 0.116973 -0.953218 -1.469869

Pt -0.121424 -0.903652 1.492746

N 2.809054 0.537931 -2.033720

N 0.997538 1.703037 -2.034919

N -2.801978 0.625288 2.028787

N -0.976876 1.767431 2.033334

N -1.410316 -2.232081 -0.745775

N -1.446673 -2.361987 0.603551

N 1.434403 -2.389775 -0.536537

N 1.398438 -2.211939 0.807035

O -5.121997 3.115262 -0.861195

O -5.094508 0.869225 -0.827776

O 5.155437 3.124781 0.853897

O 5.055957 0.890999 0.638389

C 1.467317 0.422882 -1.940820

C 3.173080 1.872692 -2.161341

C 2.034525 2.606818 -2.162211

C 3.755346 -0.560355 -1.947404

C -0.400587 1.864842 -1.901081

C -1.084355 0.638132 -1.770105

C -2.452287 0.722113 -1.519858

C -3.097205 1.962605 -1.400050

C -2.380314 3.149795 -1.564189

C -1.015850 3.107497 -1.820623

C -4.521660 2.070572 -1.019120

C -6.457840 0.893821 -0.419381

C -1.461108 0.492608 1.941150

C -3.149803 1.964913 2.154259

C -2.002449 2.684418 2.157451

C -3.766497 -0.455732 1.924877

C 0.421837 1.915738 1.895598

C 1.093519 0.682964 1.762778

C 2.458244 0.755560 1.490711

C 3.111004 1.990804 1.359078

C 2.408119 3.184198 1.537985

C 1.046475 3.153248 1.808794

C 4.526947 2.088987 0.946101

C 6.419177 0.906570 0.227947

C -2.304434 -3.077283 -1.280932

C -2.940785 -3.784737 -0.262101

C -2.357230 -3.298267 0.906812

C 2.340717 -3.340370 -0.806073

C 2.921498 -3.788009 0.379667

C 2.288331 -3.041888 1.372453

H 4.208761 2.173958 -2.252539

H 1.883149 3.673686 -2.246134

H 3.380549 -1.408940 -2.524005

H 3.902026 -0.861499 -0.902882

H 4.709680 -0.231826 -2.368910

H -3.027581 -0.190462 -1.369112

H -2.899526 4.099732 -1.459997

H -0.443912 4.029361 -1.916927

H -6.747503 -0.149698 -0.280632

H -7.083209 1.363708 -1.185816

H -4.181880 2.278450 2.243068

H -1.837722 3.749304 2.240976

H -3.910363 -0.744763 0.876060

H -4.718816 -0.113871 2.340511

H -3.412237 -1.316849 2.495433

H 3.024033 -0.161716 1.331695

H 2.934240 4.129682 1.428192

H 0.483098 4.079870 1.909624

H 7.057198 1.315754 1.018188

H 6.545308 1.511203 -0.677650

H -2.427673 -3.129199 -2.357794

H -3.705394 -4.545223 -0.357718

H -2.544117 -3.562580 1.942797

H 2.527738 -3.641132 -1.831974

H 3.682822 -4.547855 0.502649

H 2.410987 -3.055544 2.450577

H 6.683370 -0.133868 0.027552

H -6.572565 1.447923 0.519326

**Table S6:** DFT-Optimized coordinates of **1-s** in the Ground State in THF

Center Coordinates (Angstroms) Center Coordinates (Angstroms)

X Y Z X Y Z

Pt -0.880576 -1.334774 0.729719

Pt 0.880576 1.334763 0.729776

N 0.887456 -3.278564 -0.959831

N -1.165933 -3.022823 -1.565492

N -0.887429 3.278613 -0.959726

N 1.165973 3.022905 -1.565359

N -1.609666 0.200448 1.960070

N -0.831218 1.304006 2.016945

N 0.831202 -1.304061 2.016912

N 1.609644 -0.200497 1.960085

C -0.258747 -2.656886 -0.609923

C 0.694007 -4.036700 -2.109029

H 1.495671 -4.618543 -2.543292

C -0.593595 -3.872773 -2.493798

H -1.135433 -4.277655 -3.336833

C 2.169683 -3.141324 -0.285549

H 2.225098 -2.151597 0.175607

H 2.969615 -3.239898 -1.025280

H 2.289104 -3.908182 0.487298

C -2.404351 -2.337152 -1.540775

C -2.482598 -1.394512 -0.494478

C -3.634890 -0.614072 -0.458534

H -3.750928 0.143205 0.315116

C -4.656877 -0.774391 -1.406824

C -4.545807 -1.744544 -2.403757

H -5.352651 -1.858069 -3.124157

C -3.406876 -2.538033 -2.478871

H -3.306584 -3.285400 -3.264576

C -5.867429 0.075670 -1.404831

O -6.809332 -0.058391 -2.160681

O -5.815893 1.041552 -0.472356

C -6.951017 1.899401 -0.417613

H -7.858521 1.326851 -0.198137

H -7.084408 2.424413 -1.369646

C 0.258774 2.656940 -0.609812

C -0.693952 4.036831 -2.108865

H -1.495604 4.618707 -2.543104

C 0.593656 3.872914 -2.493624

H 1.135508 4.277841 -3.336627

C -2.169674 3.141321 -0.285490

H -2.225030 2.151629 0.175749

H -2.969584 3.239766 -1.025263

H -2.289188 3.908240 0.487282

C 2.404375 2.337199 -1.540677

C 2.482606 1.394526 -0.494408

C 3.634889 0.614073 -0.458476

H 3.750904 -0.143239 0.315144

C 4.656883 0.774404 -1.406755

C 4.545833 1.744590 -2.403658

H 5.352685 1.858129 -3.124046

C 3.406913 2.538099 -2.478757

H 3.306642 3.285498 -3.264434

C 5.867426 -0.075671 -1.404776

O 6.809350 0.058418 -2.160596

O 5.815837 -1.041631 -0.472386

C 6.950943 -1.899508 -0.417674

H 7.858447 -1.326991 -0.198111

H 7.084363 -2.424438 -1.369747

C -2.665104 0.380037 2.771032

H -3.413638 -0.399616 2.871456

C -2.575178 1.631788 3.376526

H -3.258945 2.075187 4.088984

C -1.395812 2.173733 2.867707

H -0.927790 3.136142 3.049626

C 1.395774 -2.173801 2.867674

H 0.927754 -3.136218 3.049557

C 2.575138 -1.631872 3.376516

H 3.258901 -2.075295 4.088962

C 2.665046 -0.380086 2.771094

H 3.413562 0.399579 2.871570

H 6.749585 -2.612604 0.383728

H -6.749703 2.612430 0.383859

**Table S7:** DFT-Optimized coordinates of **1-TS** in the Ground State in THF

Center Coordinates (Angstroms) Center Coordinates (Angstroms)

X Y Z X Y Z

Pt -0.431330 0.828752 -1.442373

Pt 0.431409 0.829716 1.442122

N 1.847447 -0.956571 -2.627634

N -0.120421 -1.819987 -2.479797

N -1.847460 -0.955066 2.628001

N 0.120478 -1.818457 2.481005

N -1.569759 2.152216 -0.262414

N -1.180206 2.195141 1.033571

N 1.180322 2.194447 -1.034661

N 1.569837 2.152409 0.261365

O -6.154268 -2.520654 -0.465851

O -5.694594 -0.372536 0.005787

O 6.154306 -2.520239 0.467364

O 5.694052 -0.372786 -0.006720

C 0.571606 -0.653315 -2.304158

C 1.949185 -2.291060 -3.001021

C 0.713475 -2.836341 -2.905878

C 2.974011 -0.038782 -2.572893

C -1.472050 -1.815730 -2.064554

C -1.885146 -0.562129 -1.570890

C -3.186080 -0.495623 -1.078252

C -4.022743 -1.622108 -1.069338

C -3.570274 -2.839218 -1.581887

C -2.281113 -2.943253 -2.090540

C -5.386881 -1.579004 -0.498676

C -0.571561 -0.651893 2.304682

C -1.949251 -2.289419 3.001851

C -0.713492 -2.834683 2.907248

C -2.974039 -0.037336 2.572680

C 1.472156 -1.814384 2.065915

C 1.885226 -0.561088 1.571460

C 3.186112 -0.494899 1.078660

C 4.022777 -1.621386 1.070407

C 3.570408 -2.838128 1.583917

C 2.281275 -2.941847 2.092709

C 5.386748 -1.578699 0.499320

C -2.598256 2.998451 -0.428209

C -2.887726 3.617190 0.786937

C -1.958921 3.077713 1.675955

C 1.959021 3.076616 -1.677614

C 2.887786 3.616719 -0.788934

C 2.598305 2.998785 0.426619

H 2.893353 -2.722609 -3.304498

H 0.367582 -3.841415 -3.101563

H 2.994432 0.465484 -1.601675

H 2.897785 0.710277 -3.367683

H 3.895760 -0.612787 -2.700695

H -3.563484 0.442630 -0.674833

H -4.234517 -3.700226 -1.565500

H -1.916081 -3.893390 -2.477560

H -2.893484 -2.720886 3.305245

H -0.367612 -3.839674 3.103373

H -3.895832 -0.611382 2.699954

H -2.993908 0.466963 1.601481

H -2.898296 0.711700 3.367543

H 3.563469 0.443082 0.674566

H 4.234675 -3.699129 1.568093

H 1.916295 -3.891721 2.480423

H -3.651822 4.356625 0.990130

H 3.651862 4.356045 -0.992602

H 3.059609 3.111875 1.402497

H 1.812715 3.260192 -2.737179

H -3.059591 3.110872 -1.404150

H -1.812605 3.261988 2.735397

C -6.988172 -0.263529 0.590451

H -7.073320 0.767016 0.939590

H -7.766810 -0.480574 -0.148398

H -7.091830 -0.959201 1.430399

C 6.987367 -0.264285 -0.592055

H 7.072335 0.765938 -0.942189

H 7.766339 -0.480648 0.146638

H 7.090648 -0.960733 -1.431411

**Table S8:** DFT-Optimized coordinates of **2-f** in the Ground State in THF

Center Coordinates (Angstroms) Center Coordinates (Angstroms)

X Y Z X Y Z

Pt -0.109301 0.778075 -1.482517

Pt 0.113869 0.749120 1.490256

N -2.793579 -0.731298 -2.072125

N -0.978201 -1.889781 -2.037970

N 2.785318 -0.790169 2.070456

N 0.956183 -1.926086 2.057987

N 1.422877 2.065498 -0.752779

N 1.457816 2.204385 0.592566

N -1.444529 2.227127 -0.559490

N -1.409585 2.058282 0.782309

O 5.128929 -3.282799 -0.776188

O 5.096976 -1.036701 -0.756213

O -5.162513 -3.277604 0.798637

O -5.057349 -1.044445 0.579408

C -1.454297 -0.610984 -1.960330

C -3.151190 -2.068328 -2.195015

C -2.010201 -2.798217 -2.173528

C -3.744970 0.364015 -2.007149

C 0.418290 -2.047629 -1.883404

C 1.099558 -0.819900 -1.753722

C 2.463969 -0.898537 -1.485040

C 3.108782 -2.137122 -1.346516

C 2.395367 -3.326642 -1.509161

C 1.034428 -3.288597 -1.783890

C 4.528625 -2.240220 -0.947539

C 6.455505 -1.056043 -0.331798

C 1.446771 -0.653992 1.964284

C 3.126984 -2.130187 2.208579

C 1.977178 -2.845745 2.200923

C 3.753673 0.288162 1.974326

C -0.441011 -2.072031 1.901117

C -1.109401 -0.839886 1.751512

C -2.469913 -0.910064 1.460332

C -3.123329 -2.144792 1.327620

C -2.425106 -3.338409 1.522736

C -1.067341 -3.308706 1.812471

C -4.533884 -2.242453 0.896573

C -6.413774 -1.059028 0.147201

C 2.338595 2.886364 -1.291269

C 2.994069 3.594180 -0.280019

C 2.391047 3.122709 0.887317

C -2.377555 3.152248 -0.833280

C -2.979898 3.597926 0.344603

C -2.324671 2.867240 1.339448

H -4.184522 -2.373486 -2.299131

H -1.853803 -3.865132 -2.247220

H -3.349778 1.220107 -2.558060

H -3.930110 0.650777 -0.964671

H -4.682618 0.039275 -2.467775

H 3.035124 0.016701 -1.334392

H 2.914364 -4.274914 -1.389877

H 0.464948 -4.212109 -1.878802

H 6.739198 -0.011375 -0.188988

H 7.091682 -1.522551 -1.091400

H 4.157053 -2.446300 2.310943

H 1.807632 -3.909522 2.288965

H 3.918507 0.565853 0.925594

H 4.696825 -0.050654 2.412855

H 3.388797 1.155765 2.528056

H -3.030277 0.007952 1.286505

H -2.951512 -4.283526 1.411078

H -0.507508 -4.235958 1.926295

H -7.065475 -1.461945 0.929447

H -6.526941 -1.668714 -0.756685

H 2.469361 2.926929 -2.368829

H 2.589521 3.395672 1.920309

H -2.576824 3.447949 -1.859793

H -2.455359 2.883055 2.417697

H -6.671970 -0.019065 -0.063438

H 6.561838 -1.610621 0.607601

C -4.052617 4.624824 0.495347

H -4.855969 4.282048 1.160710

H -3.671287 5.567294 0.912302

H -4.507447 4.861076 -0.474792

C 4.067600 4.623543 -0.406119

H 4.879099 4.289970 -1.066284

H 3.690100 5.570826 -0.815517

H 4.510896 4.847906 0.572165

**Table S9:** DFT-Optimized coordinates of **2-s** in the Ground State in THF

Center Coordinates (Angstroms) Center Coordinates (Angstroms)

X Y Z X Y Z

Pt -0.853444 -1.365025 0.571250

Pt 0.853458 1.365074 0.571428

N 0.970090 -3.261694 -1.121659

N -1.099359 -3.093749 -1.703415

N -0.969881 3.262045 -1.121330

N 1.099553 3.093949 -1.703101

N -1.627420 0.167569 1.794812

N -0.872295 1.283462 1.857120

N 0.872192 -1.283504 1.857103

N 1.627331 -0.167613 1.794941

C -0.195005 -2.681498 -0.764986

C 0.791848 -4.041877 -2.258638

H 1.609993 -4.597682 -2.696319

C -0.505945 -3.933181 -2.628357

H -1.041983 -4.369796 -3.459112

C 2.255582 -3.060499 -0.469869

H 2.261331 -2.076616 0.006576

H 3.044897 -3.100665 -1.226380

H 2.434353 -3.831010 0.287680

C -2.362785 -2.455343 -1.673526

C -2.466935 -1.498026 -0.643317

C -3.649465 -0.764918 -0.604768

H -3.785221 0.001012 0.156983

C -4.676961 -0.985285 -1.535136

C -4.537890 -1.966484 -2.517667

H -5.348705 -2.126533 -3.224617

C -3.367873 -2.712827 -2.595043

H -3.247158 -3.469135 -3.369222

C -5.924528 -0.190452 -1.526752

O -6.864369 -0.368039 -2.276196

O -5.911396 0.776891 -0.594534

C -7.082188 1.584638 -0.533363

H -7.962780 0.973767 -0.307628

H -7.244162 2.102800 -1.484715

C 0.195159 2.681716 -0.764702

C -0.791564 4.042309 -2.258243

H -1.609658 4.598222 -2.695882

C 0.506220 3.933522 -2.627968

H 1.042299 4.370152 -3.458687

C -2.255397 3.060895 -0.469573

H -2.261118 2.077084 0.007020

H -3.044684 3.100908 -1.226123

H -2.434234 3.831513 0.287852

C 2.362916 2.455412 -1.673262

C 2.466970 1.498013 -0.643117

C 3.649439 0.764807 -0.604595

H 3.785125 -0.001173 0.157120

C 4.676967 0.985148 -1.534931

C 4.537991 1.966418 -2.517405

H 5.348834 2.126450 -3.224326

C 3.368041 2.712871 -2.594747

H 3.247410 3.469251 -3.368869

C 5.924467 0.190211 -1.526576

O 6.864310 0.367731 -2.276033

O 5.911205 -0.777244 -0.594475

C 7.081896 -1.585145 -0.533397

H 7.962567 -0.974409 -0.307606

H 7.243798 -2.103230 -1.484804

C -2.705923 0.338589 2.578191

H -3.445637 -0.451816 2.671248

C -2.661618 1.599621 3.177225

C -1.477672 2.150951 2.683676

H -1.040076 3.128860 2.866772

C 1.477491 -2.151040 2.683669

H 1.039880 -3.128958 2.866685

C 2.661391 -1.599751 3.177364

C 2.705759 -0.338677 2.578410

H 3.445447 0.451737 2.671588

H 6.906485 -2.307003 0.266364

H -6.906863 2.306435 0.266471

C -3.631318 2.208703 4.134224

H -3.524275 1.802388 5.149560

H -3.486025 3.293964 4.203409

H -4.670224 2.033326 3.826049

C 3.630994 -2.208977 4.134369

H 3.525339 -1.801206 5.149262

H 3.484169 -3.293935 4.205038

H 4.669926 -2.035522 3.825172

**Table S10:** DFT-Optimized coordinates of **2-TS** in the Ground State in THF

Center Coordinates (Angstroms) Center Coordinates (Angstroms)

X Y Z X Y Z

Pt 0.396563 0.670927 1.457664

Pt -0.396616 0.671472 -1.457515

N -1.907063 -1.140255 2.566893

N 0.070628 -1.989231 2.474684

N 1.907241 -1.139333 -2.566791

N -0.070394 -1.988481 -2.474962

N 1.571445 1.997921 0.298034

N 1.219903 2.038542 -1.006040

N -1.220029 2.038073 1.006665

N -1.571549 1.997991 -0.297430

O 6.163630 -2.646708 0.625131

O 5.700045 -0.500423 0.147799

O -6.163794 -2.646478 -0.626846

O -5.699999 -0.500555 -0.148086

C -0.624023 -0.826117 2.287112

C -2.011681 -2.479051 2.923855

C -0.769803 -3.015209 2.863448

C -3.037065 -0.228445 2.487109

C 1.434162 -1.975343 2.100061

C 1.854801 -0.719325 1.620820

C 3.167045 -0.643750 1.162572

C 4.010430 -1.764909 1.175161

C 3.551786 -2.985179 1.674772

C 2.249746 -3.097696 2.147853

C 5.388666 -1.710880 0.640558

C 0.624122 -0.825311 -2.287247

C 2.012066 -2.478146 -2.923615

C 0.770219 -3.014409 -2.863466

C 3.037106 -0.227359 -2.486988

C -1.434007 -1.974707 -2.100617

C -1.854761 -0.718820 -1.621129

C -3.167063 -0.643410 -1.163023

C -4.010431 -1.764576 -1.176084

C -3.551712 -2.984677 -1.676039

C -2.249583 -3.097044 -2.148915

C -5.388720 -1.710732 -0.641597

C 2.624546 2.810283 0.482739

C 2.976394 3.411587 -0.728823

C 2.049743 2.887720 -1.632085

C -2.049912 2.886948 1.633061

C -2.976573 3.411162 0.730011

C -2.624682 2.810389 -0.481803

H -2.962218 -2.919501 3.192742

H -0.423383 -4.020145 3.058836

H -3.032214 0.285290 1.520653

H -2.987962 0.513842 3.290291

H -3.958550 -0.809044 2.583166

H 3.546558 0.297319 0.767992

H 4.221710 -3.841970 1.676636

H 1.880316 -4.050098 2.525146

H 2.962701 -2.918524 -3.192273

H 0.423931 -4.019388 -3.058868

H 3.958671 -0.807767 -2.583420

H 3.032374 0.286126 -1.520401

H 2.987688 0.515135 -3.289959

H -3.546643 0.297520 -0.768176

H -4.221637 -3.841466 -1.678300

H -1.880084 -4.049325 -2.526449

H -3.065694 2.918468 -1.468775

H -1.948506 3.068162 2.699677

H 3.065564 2.917961 1.469752

H 1.948318 3.069385 -2.698622

C 7.007028 -0.378092 -0.403003

H 7.091861 0.654742 -0.745830

H 7.768513 -0.591152 0.354669

H 7.138163 -1.069239 -1.242866

C -7.007040 -0.378409 0.402618

H -7.091831 0.654251 0.745982

H -7.768447 -0.591008 -0.355261

H -7.138335 -1.069989 1.242099

C -4.070369 4.389315 1.002785

H -3.884803 5.363492 0.530039

H -5.040598 4.032358 0.631995

H -4.175455 4.566190 2.080407

C 4.070165 4.389883 -1.001192

H 3.884751 5.363740 -0.527729

H 5.040471 4.032634 -0.630887

H 4.174995 4.567494 -2.078718

**Table S11:** DFT-Optimized coordinates of **3-f** in the Ground State in THF

Center Coordinates (Angstroms) Center Coordinates (Angstroms)

X Y Z X Y Z

Pt -0.006841 0.681052 -1.486696

Pt 0.006884 0.681312 1.486701

N 2.642339 -0.853549 -2.127860

N 0.808612 -1.985940 -2.131801

N -2.642359 -0.853155 2.127924

N -0.808661 -1.985593 2.132086

N -1.468820 1.986437 -0.657162

N -1.374382 2.137310 0.689202

N 1.374453 2.137177 -0.689452

N 1.468905 1.986510 0.656932

O -5.296113 -3.347637 -0.794925

O -5.188002 -1.117935 -0.542134

O 5.295924 -3.347655 0.794960

O 5.188009 -1.117927 0.542338

C 1.303730 -0.716573 -2.022694

C 2.980570 -2.192685 -2.280192

C 1.829285 -2.906554 -2.280574

C 3.609229 0.226131 -2.016619

C -0.585154 -2.132754 -1.937656

C -1.241306 -0.903374 -1.723026

C -2.595983 -0.975853 -1.408610

C -3.258317 -2.209307 -1.314712

C -2.571977 -3.398769 -1.569051

C -1.219433 -3.367405 -1.884648

C -4.667265 -2.311254 -0.879023

C -6.530342 -1.145799 -0.069454

C -1.303739 -0.716223 2.022825

C -2.980633 -2.192265 2.280373

C -1.829369 -2.906166 2.280882

C -3.609225 0.226533 2.016520

C 0.585096 -2.132478 1.937907

C 1.241296 -0.903139 1.723195

C 2.595966 -0.975700 1.408752

C 3.258234 -2.209193 1.314877

C 2.571840 -3.398610 1.569276

C 1.219311 -3.367162 1.884928

C 4.667163 -2.311226 0.879144

C -2.312723 2.914375 -1.139362

C -2.784519 3.680377 -0.071805

C -2.153482 3.161902 1.065063

C -2.574022 3.028670 -2.600565

C -2.234043 3.575840 2.492879

C 2.153541 3.161724 -1.065465

C 2.784590 3.680367 0.071318

C 2.312810 2.914518 1.138992

C 2.234114 3.575476 -2.493336

C 2.574021 3.029087 2.600188

C 6.530355 -1.145875 0.069683

H 4.009785 -2.509162 -2.391008

H 1.658256 -3.969042 -2.379905

H 4.596939 -0.166212 -2.273405

H 3.630401 0.616538 -0.992309

H 3.347712 1.030829 -2.709051

H -3.144962 -0.059284 -1.194683

H -3.104075 -4.343914 -1.489249

H -0.671423 -4.293554 -2.051992

H -6.612159 -1.779260 0.821413

H -7.205817 -1.530568 -0.840698

H -4.009863 -2.508687 2.391200

H -1.658369 -3.968645 2.380360

H -4.596989 -0.165845 2.273035

H -3.630119 0.617026 0.992240

H -3.347912 1.031186 2.709091

H 3.144990 -0.059164 1.194815

H 3.103877 -4.343790 1.489475

H 0.671259 -4.293276 2.052332

H -3.478651 4.512678 -0.120261

H -2.814190 2.051655 -3.042091

H -1.691849 3.413175 -3.133129

H -3.409401 3.710108 -2.797648

H -1.573722 2.943531 3.103412

H -3.254685 3.495068 2.892463

H -1.918574 4.618623 2.628210

H 3.478720 4.512678 0.119636

H 1.573412 2.943425 -3.103722

H 3.254662 3.494158 -2.893056

H 1.919151 4.618402 -2.628718

H 2.812794 2.051919 3.042124

H 1.692278 3.414994 3.132456

H 3.410270 3.709495 2.797146

H 6.612187 -1.779448 -0.821100

H 7.205808 -1.530564 0.840989

H -6.783381 -0.112131 0.175010

H 6.783421 -0.112240 -0.174891

**Table S12:** DFT-Optimized coordinates of **3-s** in the Ground State in THF

Center Coordinates (Angstroms) Center Coordinates (Angstroms)

X Y Z X Y Z

Pt 0.772499 -1.346340 0.536643

Pt -0.772478 1.346180 0.536899

N -1.147227 -3.052260 -1.262404

N 0.897896 -2.839986 -1.911396

N 1.147241 3.052420 -1.261832

N -0.897910 2.840397 -1.910796

N 1.614781 0.089401 1.836830

N 0.919863 1.253468 1.863600

N -0.919850 -1.253886 1.863347

N -1.614726 -0.089788 1.836878

O 6.692640 -0.149420 -2.395400

O 5.855453 0.737724 -0.507880

O -6.692697 0.150075 -2.395348

O -5.855539 -0.737522 -0.508035

C 0.039334 -2.523693 -0.893451

C -1.028589 -3.701730 -2.486203

C 0.253831 -3.564932 -2.897071

C -2.405232 -2.922322 -0.544067

C 2.174285 -2.229199 -1.865695

C 2.337139 -1.397037 -0.740435

C 3.531681 -0.688135 -0.666591

C 4.523197 -0.821753 -1.649585

C 4.327779 -1.681845 -2.731833

C 3.139488 -2.393831 -2.850063

C 5.793517 -0.068289 -1.582157

C 7.056013 1.491982 -0.377186

C -0.039330 2.523809 -0.892965

C 1.028569 3.702257 -2.485432

C -0.253879 3.565644 -2.896270

C 2.405295 2.922031 -0.543662

C -2.174324 2.229665 -1.865171

C -2.337154 1.397211 -0.740125

C -3.531706 0.688310 -0.666422

C -4.523267 0.822239 -1.649336

C -4.327880 1.682638 -2.731343

C -3.139567 2.394606 -2.849443

C -5.793597 0.068774 -1.582096

C 2.655312 0.181999 2.683290

C 2.627682 1.449491 3.273596

C 1.511785 2.089586 2.731530

C 3.609289 -0.944727 2.877903

C 0.964573 3.451335 2.980477

C -1.511747 -2.090165 2.731149

C -2.627596 -1.450149 3.273402

C -2.655249 -0.182550 2.683325

C -0.964541 -3.451968 2.979811

C -3.609229 0.944140 2.878139

C -7.056070 -1.491869 -0.377602

H -1.872928 -4.197102 -2.946400

H 0.749078 -3.912281 -3.792808

H -3.193913 -2.632218 -1.246394

H -2.297190 -2.144439 0.215732

H -2.675418 -3.870554 -0.065752

H 3.700070 -0.007415 0.166000

H 5.111110 -1.777007 -3.480413

H 2.975785 -3.054268 -3.700408

H 7.195593 2.149770 -1.241768

H 7.922641 0.827617 -0.293355

H 1.872909 4.197718 -2.945533

H -0.749167 3.913317 -3.791860

H 2.675539 3.869954 -0.064772

H 3.193912 2.632356 -1.246235

H 2.297281 2.143698 0.215675

H -3.700054 0.007336 0.165972

H -5.111241 1.778030 -3.479862

H -2.975867 3.055252 -3.699626

H 3.313215 1.842268 4.016930

H 4.509751 -0.833206 2.255436

H 3.137379 -1.898465 2.606526

H 3.939006 -1.006144 3.921866

H 0.972840 4.065095 2.067714

H 1.550848 3.974028 3.744533

H -0.079086 3.403381 3.321663

H -3.313110 -1.843070 4.016677

H -0.971854 -4.065160 2.066663

H -1.551448 -3.975207 3.743007

H 0.078789 -3.404073 3.322016

H -4.509746 0.832667 2.255743

H -3.137355 1.897911 2.606815

H -3.938850 1.005457 3.922138

H -7.195584 -2.149421 -1.242375

H -7.922730 -0.827565 -0.293622

H -6.941633 -2.082229 0.533236

H 6.941563 2.082091 0.533812

**Table S13:** DFT-Optimized coordinates of **3-TS** in the Ground State in THF

Center Coordinates (Angstroms) Center Coordinates (Angstroms)

X Y Z X Y Z

Pt -0.399501 0.611777 -1.456692

Pt 0.399481 0.611771 1.456719

N 1.899421 -1.176731 -2.608628

N -0.071392 -2.040844 -2.495358

N -1.899396 -1.176750 2.608693

N 0.071408 -2.040871 2.495337

N -1.557585 1.947502 -0.293460

N -1.207771 1.982203 1.016706

N 1.207693 1.982242 -1.016667

N 1.557548 1.947534 0.293489

O -6.105840 -2.777059 -0.490105

O -5.642103 -0.639808 0.024954

O 6.105826 -2.777045 0.489998

O 5.642128 -0.639759 -0.024948

C 0.618370 -0.874745 -2.306833

C 2.007951 -2.511285 -2.980675

C 0.770852 -3.056909 -2.906634

C 3.024991 -0.258619 -2.534406

C -1.426710 -2.041608 -2.089344

C -1.847135 -0.793205 -1.589347

C -3.144719 -0.737119 -1.087628

C -3.977050 -1.866585 -1.086184

C -3.521715 -3.076292 -1.614428

C -2.232470 -3.171597 -2.124792

C -5.337816 -1.835505 -0.507613

C -0.618358 -0.874770 2.306848

C -2.007916 -2.511304 2.980744

C -0.770822 -3.056933 2.906649

C -3.024946 -0.258611 2.534542

C 1.426715 -2.041623 2.089290

C 1.847133 -0.793204 1.589324

C 3.144711 -0.737109 1.087587

C 3.977045 -1.866575 1.086108

C 3.521710 -3.076300 1.614312

C 2.232470 -3.171618 2.124683

C 5.337816 -1.835481 0.507549

C -2.543120 2.835952 -0.505605

C -2.840980 3.460163 0.707365

C -1.964692 2.897061 1.641527

C 1.964662 2.897023 -1.641535

C 2.841055 3.460046 -0.707426

C 2.543181 2.835892 0.505572

H 2.957656 -2.942431 -3.267169

H 0.429404 -4.062797 -3.105863

H 2.981967 0.301307 -1.595090

H 3.006473 0.441880 -3.376363

H 3.950572 -0.840012 -2.565624

H -3.518617 0.195970 -0.667964

H -4.183169 -3.939582 -1.606119

H -1.864749 -4.117444 -2.519896

H -2.957608 -2.942447 3.267284

H -0.429369 -4.062821 3.105869

H -3.950543 -0.839972 2.565931

H -2.982029 0.301245 1.595178

H -3.006283 0.441947 3.376447

H 3.518601 0.195987 0.667935

H 4.183161 -3.939591 1.605966

H 1.864748 -4.117479 2.519753

H -3.580013 4.235452 0.879114

H 3.580148 4.235268 -0.879220

C -6.932424 -0.543428 0.618823

H -7.016422 0.480084 0.988398

H -7.715039 -0.747049 -0.119623

H -7.030621 -1.255440 1.445758

C 6.932460 -0.543363 -0.618791

H 7.016476 0.480167 -0.988309

H 7.715061 -0.747034 0.119656

H 7.030661 -1.255331 -1.445764

C -3.119809 3.039837 -1.862972

H -3.822475 3.880664 -1.864739

H -2.329986 3.250517 -2.597945

H -3.658058 2.148692 -2.216428

C -1.784023 3.176235 3.092558

H -1.337329 4.165262 3.262718

H -2.738444 3.154334 3.635162

H -1.113112 2.428771 3.538448

C 1.783844 3.176278 -3.092532

H 1.336711 4.165130 -3.262590

H 2.738260 3.154823 -3.635148

H 1.113214 2.428600 -3.538475

C 3.120000 3.039716 1.862894

H 3.822450 3.880724 1.864693

H 2.330225 3.250079 2.598003

H 3.658538 2.148662 2.216144

**Table S14:** DFT-Optimized coordinates of **4-f** in the Ground State in DMSO

Center Coordinates (Angstroms) Center Coordinates (Angstroms)

X Y Z X Y Z

Pt -0.439929 -1.414234 0.125722

Pt 0.417246 1.397201 -0.193323

N 2.008003 -2.523588 1.758421

N 0.276847 -1.870809 2.859704

N -1.971252 2.893911 1.188347

N -0.208867 2.515338 2.367015

N -1.695772 -0.366057 -1.249770

N -1.151509 0.812976 -1.631723

N 1.090852 -1.177874 -1.435910

N 1.645127 0.052158 -1.324917

O -5.282624 1.098578 3.966080

O -5.171720 0.976222 1.724632

O 5.416388 -0.105237 3.953027

O 5.202376 -0.545851 1.759135

C 0.745747 -2.074474 1.591186

C 2.326156 -2.584852 3.109395

C 1.238203 -2.175845 3.804261

C 2.945386 -2.848373 0.696536

C -1.015014 -1.311574 2.971734

C -1.637409 -1.119957 1.723074

C -2.875747 -0.486100 1.740147

C -3.459355 -0.062656 2.943026

C -2.816766 -0.297620 4.160559

C -1.578361 -0.927811 4.182263

C -4.719706 0.710390 2.961132

C -6.353431 1.767976 1.653711

C -0.716093 2.405484 1.101218

C -2.249913 3.285080 2.492096

C -1.142776 3.047990 3.235251

C -2.934039 2.964836 0.102125

C 1.088092 1.994258 2.569923

C 1.664531 1.496704 1.385470

C 2.906813 0.884310 1.510965

C 3.539441 0.770718 2.756851

C 2.942931 1.303267 3.901889

C 1.701874 1.923291 3.814402

C 4.805241 0.022392 2.910202

C -2.866060 -0.540792 -1.899216

C -3.071570 0.558513 -2.741836

C -1.956782 1.377233 -2.553681

C 1.884879 -1.941114 -2.212158

C 3.004667 -1.194649 -2.581872

C 2.813293 0.065344 -2.002115

C 6.384902 -1.335786 1.833961

H 3.293551 -2.933471 3.447430

H 1.072369 -2.083620 4.868275

H 3.791138 -3.385326 1.134425

H 3.312296 -1.935640 0.210587

H 2.457048 -3.487935 -0.043947

H -3.389304 -0.279865 0.802761

H -3.282459 0.046064 5.081384

H -1.054616 -1.083512 5.124089

H -6.188609 2.745872 2.120161

H -7.186995 1.266924 2.156737

H -3.207449 3.712122 2.760885

H -0.946589 3.214126 4.285045

H -3.779068 3.575999 0.429946

H -3.294791 1.963492 -0.162807

H -2.471770 3.431080 -0.772720

H 3.389022 0.456166 0.634470

H 3.446111 1.199904 4.860539

H 1.215529 2.313397 4.707108

H -3.884338 0.704014 -3.445076

H 3.814314 -1.498323 -3.236554

H 6.247697 -2.165091 2.537055

H 7.237475 -0.729199 2.156685

H -6.568540 1.888266 0.590138

H 6.551970 -1.719352 0.825464

C 3.723105 1.214545 -2.028916

C 3.272046 2.539929 -2.048255

C 5.103550 0.975618 -2.014492

C 4.178097 3.595237 -2.056102

H 2.202217 2.745674 -2.053586

C 6.008027 2.030851 -2.024924

H 5.462645 -0.052916 -1.969826

C 5.548433 3.345857 -2.046505

H 3.807887 4.619400 -2.076941

H 7.077080 1.825357 -2.008019

H 6.255829 4.173260 -2.054304

C -1.603643 2.643871 -3.194805

C -0.262101 2.947718 -3.457889

C -2.589962 3.568037 -3.555851

C 0.088523 4.159650 -4.041180

H 0.499588 2.207407 -3.214508

C -2.239337 4.776316 -4.149670

H -3.636250 3.339477 -3.348800

C -0.900123 5.079327 -4.387088

H 1.137775 4.380420 -4.236573

H -3.016119 5.489058 -4.421580

H -0.628138 6.027650 -4.847136

C 1.521412 -3.319487 -2.541542

C 2.501751 -4.305946 -2.694471

C 0.175661 -3.672461 -2.701312

C 2.141264 -5.618765 -2.981156

H 3.551536 -4.039303 -2.565445

C -0.184719 -4.986186 -2.976746

H -0.583063 -2.894693 -2.619798

C 0.798161 -5.964801 -3.115113

H 2.913500 -6.378188 -3.091901

H -1.237464 -5.242785 -3.093201

H 0.518421 -6.994044 -3.332746

C -3.750496 -1.687165 -1.670669

C -3.265958 -2.954899 -1.323628

C -5.135333 -1.507466 -1.783148

C -4.142409 -4.010769 -1.097573

H -2.191689 -3.111179 -1.221331

C -6.010058 -2.564407 -1.559801

H -5.525127 -0.518997 -2.026745

C -5.517121 -3.821339 -1.216207

H -3.744875 -4.989649 -0.832507

H -7.083357 -2.403199 -1.646398

H -6.201863 -4.649009 -1.039706

**Table S15:** DFT-Optimized coordinates of **4-s** in the Ground State in DMSO

Center Coordinates (Angstroms) Center Coordinates (Angstroms)

X Y Z X Y Z

Pt -1.220149 0.948506 -0.272863

Pt 1.220159 -0.948278 -0.272953

N 0.037574 3.123704 -2.133646

N -1.822985 2.235565 -2.766323

N -0.037634 -3.123160 -2.134149

N 1.823088 -2.235104 -2.766483

N -1.462593 -0.674291 1.066736

N -0.386457 -1.488041 1.103741

N 0.386495 1.488081 1.103937

N 1.462633 0.674325 1.066872

O -6.878348 -1.699323 -2.705220

O -5.714873 -2.313214 -0.883923

O 6.878508 1.699644 -2.705105

O 5.715523 2.312940 -0.883288

C -0.913305 2.247794 -1.746530

C -0.280471 3.661686 -3.376032

C -1.447003 3.102144 -3.776297

C 1.238929 3.465430 -1.386470

C -2.875863 1.297485 -2.649401

C -2.761755 0.497641 -1.494746

C -3.757319 -0.454527 -1.298932

C -4.820133 -0.592179 -2.203734

C -4.891864 0.224600 -3.334052

C -3.910297 1.180887 -3.568052

C -5.907082 -1.571885 -1.984903

C -6.752317 -3.237980 -0.573832

C 0.913276 -2.247362 -1.746810

C 0.280551 -3.661040 -3.376544

C 1.447168 -3.101539 -3.776602

C -1.239282 -3.464737 -1.387368

C 2.876003 -1.297085 -2.649386

C 2.761870 -0.497298 -1.494689

C 3.757506 0.454778 -1.298752

C 4.820413 0.592368 -2.203463

C 4.892152 -0.224350 -3.333823

C 3.910507 -1.180521 -3.567958

C 5.907467 1.571932 -1.984532

C -2.348095 -1.067443 2.005309

C -1.821916 -2.176847 2.669472

C -0.571455 -2.399663 2.081803

C 0.571450 2.399520 2.082164

C 1.821905 2.176645 2.669834

C 2.348125 1.067368 2.005482

C 6.753036 3.237626 -0.573202

H 0.358883 4.390060 -3.856173

H -2.022021 3.239933 -4.681090

H 2.077647 3.561384 -2.082472

H 1.451410 2.665456 -0.672586

H 1.102392 4.410809 -0.848934

H -3.724639 -1.097312 -0.421104

H -5.726237 0.105965 -4.021492

H -3.964736 1.824064 -4.444907

H -6.877287 -3.966464 -1.381871

H -7.700513 -2.712675 -0.415401

H -0.358770 -4.389354 -3.856817

H 2.022334 -3.239339 -4.681299

H -1.103295 -4.410503 -0.850378

H -2.077944 -3.559902 -2.083556

H -1.451497 -2.665078 -0.673065

H 3.724859 1.097510 -0.420869

H 5.726569 -0.105729 -4.021212

H 3.964950 -1.823640 -4.444854

H -2.277456 -2.729246 3.484149

H 2.277389 2.728877 3.484656

H 6.877618 3.966482 -1.380975

H 7.701336 2.712327 -0.415391

H 6.442927 3.739535 0.345316

H -6.441866 -3.740286 0.344359

C -0.468050 3.360966 2.466407

C -1.319242 3.955904 1.526328

C -0.654234 3.659959 3.821756

C -2.344406 4.803253 1.933877

H -1.184768 3.739167 0.467568

C -1.676080 4.511126 4.228205

H -0.005275 3.193940 4.562987

C -2.530176 5.080522 3.286487

H -2.999583 5.251316 1.188161

H -1.812000 4.722513 5.287559

H -3.335128 5.740623 3.604865

C 3.626357 0.369859 2.183072

C 4.819530 1.097937 2.221448

C 3.678656 -1.027709 2.261111

C 6.044131 0.442248 2.291971

H 4.779067 2.185864 2.156313

C 4.903961 -1.682285 2.331329

H 2.751271 -1.603045 2.266890

C 6.089905 -0.949731 2.336300

H 6.966977 1.020605 2.300571

H 4.927437 -2.770317 2.388090

H 7.048784 -1.463199 2.384426

C 0.467885 -3.361359 2.465852

C 1.319136 -3.956098 1.525697

C 0.653788 -3.660907 3.821118

C 2.344055 -4.803813 1.933100

H 1.184927 -3.738918 0.466997

C 1.675405 -4.512416 4.227420

H 0.004799 -3.195051 4.562425

C 2.529542 -5.081635 3.285634

H 2.999267 -5.251720 1.187322

H 1.811106 -4.724220 5.286719

H 3.334308 -5.742020 3.603898

C -3.626406 -0.370090 2.182923

C -4.819478 -1.098350 2.221063

C -3.678926 1.027457 2.261198

C -6.044184 -0.442860 2.291509

H -4.778855 -2.186259 2.155714

C -4.904338 1.681836 2.331365

H -2.751642 1.602958 2.267177

C -6.090175 0.949107 2.336058

H -6.966944 -1.021353 2.299940

H -4.927978 2.769855 2.388286

H -7.049137 1.462425 2.384113

**Table S16:** DFT-Optimized coordinates of **1-f** in the Triplet State in THF

Center Coordinates (Angstroms) Center Coordinates (Angstroms)

X Y Z X Y Z

Pt -0.877614 -1.302001 0.743377

Pt 0.906750 1.365681 0.700790

N 0.892098 -3.296353 -0.896508

N -1.158067 -3.046591 -1.514943

N -0.860312 3.309845 -1.014646

N 1.199383 2.993571 -1.651317

N -1.615685 0.268514 1.944687

N -0.833792 1.369464 1.986119

N 0.837042 -1.253155 2.049663

N 1.621870 -0.156160 1.982833

C -0.251487 -2.659532 -0.568353

C 0.697898 -4.084782 -2.024883

H 1.497936 -4.681943 -2.440981

C -0.587885 -3.925088 -2.417583

H -1.129654 -4.350389 -3.250536

C 2.172344 -3.152695 -0.220067

H 2.211980 -2.170507 0.257693

H 2.974214 -3.227398 -0.960579

H 2.301247 -3.930661 0.540152

C -2.400140 -2.367773 -1.503931

C -2.484081 -1.399150 -0.482713

C -3.646052 -0.633632 -0.456363

H -3.766744 0.140383 0.299773

C -4.673109 -0.832670 -1.391988

C -4.554710 -1.824490 -2.366465

H -5.365183 -1.967426 -3.077429

C -3.405811 -2.604160 -2.430198

H -3.301641 -3.370633 -3.196665

C -5.898468 -0.003740 -1.398405

O -6.837776 -0.162336 -2.152642

O -5.864384 0.971187 -0.474980

C -7.013380 1.810914 -0.429358

H -7.911461 1.225962 -0.203920

H -7.154999 2.323663 -1.386841

C 0.279728 2.662984 -0.661923

C -0.656155 4.044383 -2.167679

H -1.450365 4.633703 -2.606755

C 0.625024 3.858449 -2.573615

H 1.159019 4.241031 -3.431340

C -2.132538 3.207417 -0.319485

H -2.268347 2.179221 0.031231

H -2.935355 3.460444 -1.017381

H -2.166174 3.886508 0.539290

C 2.412198 2.361774 -1.603135

C 2.486273 1.379031 -0.482979

C 3.611269 0.564341 -0.443368

H 3.700425 -0.185963 0.339985

C 4.639746 0.681846 -1.387670

C 4.555631 1.722173 -2.425784

H 5.391804 1.813225 -3.114539

C 3.464360 2.531716 -2.528790

H 3.403156 3.285938 -3.312272

C 5.797702 -0.188714 -1.403204

O 6.722503 -0.104820 -2.202891

O 5.762041 -1.136826 -0.434338

C 6.875241 -2.016592 -0.409324

H 7.807527 -1.466315 -0.237229

H 6.962647 -2.565878 -1.354014

C -2.673641 0.466260 2.747084

H -3.425583 -0.308652 2.858063

C -2.581367 1.728099 3.331917

H -3.266187 2.186104 4.034014

C -1.398134 2.256537 2.819140

H -0.925504 3.218984 2.988664

C 1.380787 -2.106368 2.930035

H 0.903689 -3.061709 3.125171

C 2.554112 -1.559381 3.447739

H 3.223170 -1.990750 4.181230

C 2.661373 -0.321570 2.817313

H 3.411724 0.456702 2.913875

H 6.692732 -2.711237 0.413539

H -6.823884 2.535491 0.364589

**Table S17:** DFT-Optimized coordinates of **1-s** in the Triplet State in THF

Center Coordinates (Angstroms) Center Coordinates (Angstroms)

X Y Z X Y Z

Pt 0.066115 0.887532 -1.373517

Pt -0.066154 0.887446 1.373388

N -2.534340 -0.774512 -2.042094

N -0.655282 -1.847230 -1.880689

N 2.534257 -0.774630 2.042060

N 0.655242 -1.847326 1.880169

N 1.497597 2.273311 -0.622014

N 1.403851 2.389487 0.723483

N -1.403718 2.389489 -0.723499

N -1.497585 2.273381 0.621986

O 5.506981 -2.860578 -0.512260

O 5.366459 -0.615980 -0.622951

O -5.507215 -2.860449 0.512529

O -5.366855 -0.615884 0.624086

C -1.203647 -0.591351 -1.849806

C -2.811281 -2.122388 -2.174336

C -1.641032 -2.802337 -2.075034

C -3.534073 0.274605 -2.066167

C 0.722907 -1.937423 -1.677526

C 1.347636 -0.673399 -1.523150

C 2.707968 -0.654237 -1.266801

C 3.438834 -1.850270 -1.139502

C 2.785070 -3.086841 -1.302378

C 1.430575 -3.137076 -1.580185

C 4.847875 -1.859990 -0.743589

C 6.712791 -0.560063 -0.174390

C 1.203621 -0.591454 1.849477

C 2.811202 -2.122541 2.173990

C 1.640951 -2.802468 2.074556

C 3.533984 0.274498 2.066682

C -0.722933 -1.937471 1.676971

C -1.347702 -0.673423 1.522968

C -2.708080 -0.654195 1.266843

C -3.438961 -1.850194 1.139498

C -2.785170 -3.086797 1.301978

C -1.430619 -3.137105 1.579506

C -4.848124 -1.859876 0.743958

C -6.713442 -0.559891 0.176290

C 2.453150 3.104351 -1.063344

C 2.998873 3.788598 0.021918

C 2.298671 3.302423 1.125774

C -2.297804 3.303100 -1.125882

C -2.997604 3.789895 -0.022041

C -2.452410 3.105284 1.063258

H -3.823581 -2.476882 -2.321596

H -1.432041 -3.861380 -2.124793

H -3.220781 1.070673 -2.748342

H -3.682351 0.692805 -1.062846

H -4.477170 -0.152801 -2.418930

H 3.217642 0.297363 -1.121813

H 3.361899 -4.001756 -1.185933

H 0.924350 -4.095613 -1.692109

H 6.961675 0.500511 -0.097956

H 7.383580 -1.055587 -0.885019

H 3.823485 -2.477030 2.321370

H 1.431943 -3.861513 2.124239

H 3.682733 0.692862 1.063500

H 4.476938 -0.152973 2.419791

H 3.220371 1.070428 2.748870

H -3.217762 0.297427 1.121957

H -3.362014 -4.001678 1.185354

H -0.924378 -4.095668 1.691114

H -7.383762 -1.055940 0.887001

H -6.819905 -1.044144 -0.801808

H 2.684866 3.162871 -2.121502

H 3.782424 4.535491 0.008514

H 2.393073 3.552014 2.177468

H -2.392096 3.552606 -2.177603

H -3.780628 4.537341 -0.008668

H -2.684088 3.164050 2.121411

H -6.962522 0.500688 0.100632

H 6.818749 -1.044868 0.803493

**Table S18:** DFT-Optimized coordinates of **1-TS** in the Triplet State in THF

Center Coordinates (Angstroms) Center Coordinates (Angstroms)

X Y Z X Y Z

Pt 0.386887 0.854556 1.430988

Pt -0.412213 0.810194 -1.474079

N -1.928390 -0.907496 2.597387

N 0.028269 -1.798113 2.454739

N 1.927354 -0.976442 -2.568410

N -0.060847 -1.859807 -2.476768

N 1.565246 2.171510 0.260164

N 1.205910 2.199414 -1.044036

N -1.221293 2.232589 0.968534

N -1.583716 2.170411 -0.333918

O 6.099205 -2.551649 0.576704

O 5.689547 -0.385129 0.144649

O -6.030356 -2.567325 -0.418016

O -5.584225 -0.410982 0.101077

C -0.647309 -0.622240 2.280719

C -2.050845 -2.242114 2.962563

C -0.822321 -2.804618 2.871185

C -3.040485 0.028057 2.544816

C 1.387406 -1.806671 2.066080

C 1.828246 -0.554608 1.594015

C 3.141823 -0.495842 1.137164

C 3.966302 -1.631274 1.139810

C 3.485644 -2.848988 1.625076

C 2.182828 -2.943871 2.099077

C 5.347139 -1.597751 0.609857

C 0.634657 -0.670269 -2.287058

C 2.039055 -2.306811 -2.925555

C 0.805656 -2.869208 -2.874555

C 3.043844 -0.050985 -2.494715

C -1.391498 -1.859193 -2.159487

C -1.834546 -0.547920 -1.611512

C -3.121501 -0.492259 -1.087879

C -3.963363 -1.611552 -1.082221

C -3.504485 -2.863828 -1.701106

C -2.249645 -2.978574 -2.221231

C -5.271505 -1.605735 -0.460693

C 2.591577 3.016911 0.438844

C 2.910314 3.619970 -0.777342

C 2.001483 3.071484 -1.680624

C -2.003867 3.134841 1.577480

C -2.907845 3.667658 0.658944

C -2.598605 3.024789 -0.538337

H -3.002832 -2.661957 3.257963

H -0.491828 -3.815346 3.063891

H -3.055866 0.531925 1.573494

H -2.948845 0.776069 3.338998

H -3.971218 -0.530782 2.673792

H 3.537029 0.442553 0.751507

H 4.140429 -3.717306 1.616647

H 1.797550 -3.893701 2.466782

H 2.995381 -2.740432 -3.186941

H 0.479252 -3.880683 -3.068115

H 3.972825 -0.616387 -2.608277

H 3.044680 0.454616 -1.523451

H 2.973274 0.699267 -3.289872

H -3.481664 0.440207 -0.657615

H -4.200043 -3.699292 -1.721298

H -1.914109 -3.916696 -2.661255

H 3.680046 4.355837 -0.971949

H -3.668991 4.417580 0.832638

H -3.037614 3.123731 -1.525894

H -1.878348 3.337467 2.636245

H 3.030800 3.140956 1.423483

H 1.879315 3.242968 -2.745227

C 7.000662 -0.284584 -0.401435

H 7.110324 0.750836 -0.728549

H 7.754904 -0.526366 0.354854

H 7.118071 -0.966196 -1.251014

C -6.844659 -0.358835 0.750026

H -6.941750 0.656835 1.139882

H -7.658605 -0.572468 0.047869

H -6.892742 -1.083421 1.571870

**Table S19:** DFT-Optimized coordinates of **2-f** in the Triplet State in THF

Center Coordinates (Angstroms) Center Coordinates (Angstroms)

X Y Z X Y Z

Pt 0.783711 1.305973 0.650267

Pt -0.819412 -1.463240 0.485271

N -1.125818 3.224121 -0.924812

N 0.941788 3.158935 -1.532479

N 1.034004 -3.194522 -1.364541

N -1.043076 -2.941449 -1.971677

N 1.632304 -0.271275 1.767855

N 0.937063 -1.427336 1.745917

N -0.915745 1.086393 1.953195

N -1.613175 -0.062489 1.851940

C 0.062657 2.668392 -0.607495

C -0.987360 4.064258 -2.023952

H -1.829305 4.610691 -2.427038

C 0.309508 4.020578 -2.409740

H 0.821358 4.515576 -3.222916

C -2.397437 2.952649 -0.271794

H -2.341726 1.976753 0.216362

H -3.188327 2.933300 -1.027889

H -2.622931 3.720286 0.476436

C 2.231274 2.575029 -1.540417

C 2.382655 1.572674 -0.560729

C 3.600016 0.898119 -0.555700

H 3.773774 0.102879 0.167124

C 4.615735 1.215805 -1.470408

C 4.426322 2.233557 -2.406257

H 5.227615 2.468526 -3.103089

C 3.220770 2.924341 -2.448606

H 3.062493 3.711461 -3.184244

C 5.905641 0.491546 -1.488605

O 6.830987 0.742595 -2.235026

O 5.951163 -0.498463 -0.581742

C 7.166583 -1.238596 -0.540826

H 8.011142 -0.583124 -0.302917

H 7.355454 -1.725664 -1.503571

C -0.135265 -2.633387 -0.963701

C 0.860363 -3.856555 -2.565499

H 1.680407 -4.372766 -3.047058

C -0.431250 -3.709216 -2.953677

H -0.950613 -4.058824 -3.834133

C 2.303551 -3.074077 -0.667475

H 2.414495 -2.051572 -0.290456

H 3.110712 -3.290902 -1.372209

H 2.355614 -3.772810 0.174224

C -2.285647 -2.375953 -1.878634

C -2.403244 -1.479353 -0.692741

C -3.562124 -0.718035 -0.601221

H -3.683948 -0.030474 0.233443

C -4.582885 -0.810223 -1.556246

C -4.454020 -1.771379 -2.663390

H -5.284415 -1.848808 -3.360873

C -3.329179 -2.525374 -2.817782

H -3.235147 -3.218883 -3.652414

C -5.774799 0.011752 -1.513438

O -6.699500 -0.056435 -2.314828

O -5.771717 0.895582 -0.484171

C -6.919803 1.724288 -0.393737

H -7.826187 1.126268 -0.243722

H -7.043320 2.325107 -1.302252

C 2.722131 -0.444557 2.533327

H 3.420249 0.374229 2.683650

C 2.747004 -1.748379 3.035608

C 1.590547 -2.321354 2.505114

H 1.202873 -3.330173 2.620568

C -1.533824 1.871687 2.849500

H -1.139820 2.859006 3.075712

C -2.669729 1.230839 3.348203

C -2.670081 0.004491 2.679583

H -3.365198 -0.827608 2.747911

H -6.754673 2.375540 0.467542

H 7.035403 -1.986559 0.243378

C -3.636011 1.733714 4.368228

H -4.675175 1.535835 4.075060

H -3.484109 1.265812 5.350732

H -3.532378 2.817127 4.506169

C 3.755763 -2.380641 3.935429

H 3.728595 -1.960388 4.950056

H 3.576813 -3.459186 4.025649

H 4.778874 -2.246614 3.559617

**Table S20:** DFT-Optimized coordinates of **2-s** in the Triplet State in THF

Center Coordinates (Angstroms) Center Coordinates (Angstroms)

X Y Z X Y Z

Pt 0.066023 0.722334 -1.373532

Pt -0.065899 0.722436 1.373445

N -2.528444 -0.952668 -2.044558

N -0.644559 -2.015959 -1.880320

N 2.528489 -0.952633 2.044631

N 0.644622 -2.015879 1.880212

N 1.497323 2.103228 -0.621018

N 1.406828 2.219067 0.722457

N -1.406656 2.219242 -0.722592

N -1.497019 2.103395 0.620900

O 5.521421 -3.000732 -0.501949

O 5.371803 -0.757335 -0.627857

O -5.521466 -3.000686 0.502349

O -5.371815 -0.757294 0.628005

C -1.198790 -0.762263 -1.851060

C -2.798875 -2.301885 -2.175551

C -1.625493 -2.976031 -2.074370

C -3.533112 0.091452 -2.073474

C 0.733938 -2.100563 -1.676392

C 1.353484 -0.834043 -1.522510

C 2.713527 -0.809568 -1.265435

C 3.449250 -2.002412 -1.136241

C 2.800647 -3.241755 -1.298886

C 1.446527 -3.297349 -1.577930

C 4.858042 -2.004448 -0.739854

C 6.717760 -0.692266 -0.180008

C 1.198883 -0.762218 1.850949

C 2.798872 -2.301859 2.175793

C 1.625479 -2.975966 2.074511

C 3.533190 0.091459 2.073546

C -0.733907 -2.100442 1.676325

C -1.353447 -0.833909 1.522562

C -2.713507 -0.809461 1.265523

C -3.449201 -2.002318 1.136278

C -2.800568 -3.241653 1.298693

C -1.446420 -3.297232 1.577640

C -4.858072 -2.004372 0.740067

C -6.717897 -0.692236 0.180468

C 2.481317 2.903012 -1.059581

C 3.055570 3.574166 0.023892

C 2.333738 3.102441 1.122498

C -2.334093 3.102108 -1.122561

C -3.056084 3.573489 -0.023924

C -2.481397 2.902664 1.059532

H -3.809441 -2.661339 -2.322458

H -1.411313 -4.034123 -2.122558

H -3.225002 0.884552 -2.761553

H -3.680844 0.516395 -1.072987

H -4.474969 -0.342887 -2.421224

H 3.219180 0.144146 -1.120467

H 3.380850 -4.154457 -1.181449

H 0.944561 -4.258074 -1.690360

H 6.961115 0.370078 -0.109167

H 7.391065 -1.187866 -0.888280

H 3.809403 -2.661317 2.322923

H 1.411285 -4.034059 2.122608

H 3.681098 0.516235 1.073016

H 4.474958 -0.342853 2.421562

H 3.224992 0.884685 2.761440

H -3.219205 0.144244 1.120640

H -3.380717 -4.154367 1.181102

H -0.944388 -4.257953 1.689816

H -7.391023 -1.187729 0.888985

H -6.827141 -1.171566 -0.799734

H 2.718982 2.954806 -2.117871

H 2.444573 3.351619 2.174061

H -2.445096 3.351253 -2.174116

H -2.718966 2.954380 2.117848

H -6.961217 0.370107 0.109567

H 6.826761 -1.171458 0.800293

C 4.166609 4.570779 0.018239

H 5.035292 4.210479 -0.548316

H 3.864612 5.527431 -0.429483

H 4.505036 4.782862 1.039912

C -4.167564 4.569612 -0.018260

H -3.866080 5.526279 0.429770

H -4.505841 4.781802 -1.039962

H -5.036230 4.208801 0.547995

**Table S21:** DFT-Optimized coordinates of **2-TS** in the Triplet State in THF

Center Coordinates (Angstroms) Center Coordinates (Angstroms)

X Y Z X Y Z

Pt -0.365747 0.691092 -1.444745

Pt 0.388697 0.641868 1.471507

N 1.961199 -1.112753 -2.523605

N -0.009448 -1.978167 -2.425440

N -1.957779 -1.176708 2.500922

N 0.043344 -2.034325 2.460939

N -1.557830 2.011074 -0.294651

N -1.231664 2.020442 1.016513

N 1.245111 2.056222 -0.956471

N 1.575238 2.004230 0.352992

O -6.142060 -2.633478 -0.711135

O -5.702832 -0.474879 -0.266998

O 6.071589 -2.657741 0.538455

O 5.610090 -0.500725 0.033726

C 0.674189 -0.806184 -2.253194

C 2.079511 -2.456317 -2.855769

C 0.842086 -3.002921 -2.792393

C 3.082577 -0.189174 -2.457483

C -1.379236 -1.966287 -2.076412

C -1.817772 -0.704021 -1.630577

C -3.142342 -0.625332 -1.210703

C -3.980128 -1.750743 -1.223173

C -3.502287 -2.978709 -1.684775

C -2.188016 -3.093816 -2.121716

C -5.372322 -1.693356 -0.726329

C -0.661775 -0.852917 2.256010

C -2.062058 -2.509547 2.851343

C -0.820597 -3.056037 2.831796

C -3.084761 -0.267262 2.393758

C 1.381964 -2.016971 2.177352

C 1.822581 -0.699289 1.644789

C 3.121465 -0.626089 1.153987

C 3.977487 -1.734171 1.164538

C 3.517371 -2.995078 1.763802

C 2.251331 -3.126594 2.253246

C 5.299500 -1.706333 0.574641

C -2.606359 2.828214 -0.479803

C -2.980955 3.401059 0.739295

C -2.072977 2.854890 1.647459

C 2.073775 2.924389 -1.556852

C 2.978840 3.447980 -0.631502

C 2.613730 2.828449 0.566571

H 3.035428 -2.892884 -3.111676

H 0.505731 -4.013969 -2.973100

H 3.082591 0.327374 -1.492706

H 3.017029 0.549458 -3.262853

H 4.009535 -0.760050 -2.558721

H -3.536234 0.321403 -0.844795

H -4.167817 -3.838881 -1.686598

H -1.804721 -4.051391 -2.471023

H -3.019605 -2.956031 3.085143

H -0.486388 -4.063618 3.032413

H -4.007741 -0.842267 2.508699

H -3.081882 0.217120 1.411499

H -3.032896 0.500808 3.172833

H 3.478323 0.312269 0.734277

H 4.220821 -3.823629 1.795910

H 1.916079 -4.071165 2.679513

H 3.035721 2.929274 1.562468

H 1.986959 3.120442 -2.622114

H -3.028356 2.960162 -1.472086

H -1.990314 3.012612 2.719387

C -7.023772 -0.349496 0.248976

H -7.121330 0.688194 0.573226

H -7.765239 -0.577467 -0.524048

H -7.173086 -1.027968 1.096102

C 6.882884 -0.424022 -0.587117

H 6.977332 0.599965 -0.955913

H 7.684510 -0.642491 0.127646

H 6.955824 -1.132064 -1.421578

C 4.071577 4.434656 -0.876502

H 3.948045 5.347092 -0.277976

H 5.060102 4.021875 -0.632783

H 4.093999 4.736327 -1.930956

C -4.079379 4.373382 1.014026

H -3.905884 5.343738 0.529390

H -5.050932 4.005441 0.657555

H -4.174009 4.559819 2.090922

**Table S22:** DFT-Optimized coordinates of **3-f** in the Triplet State in THF

Center Coordinates (Angstroms) Center Coordinates (Angstroms)

X Y Z X Y Z

Pt 0.636022 1.392431 0.586785

Pt -0.657537 -1.414241 0.533249

N -1.417698 3.025921 -1.142172

N 0.603478 2.869582 -1.874174

N 1.374192 -3.004256 -1.265609

N -0.690720 -2.873624 -1.943972

N 1.614147 0.008011 1.848267

N 1.040073 -1.220216 1.850357

N -1.014607 1.135789 1.943040

N -1.596619 -0.086765 1.887062

C -0.202068 2.535232 -0.818839

C -1.370941 3.664045 -2.376175

H -2.249017 4.128984 -2.803844

C -0.102957 3.563427 -2.838821

H 0.344857 3.919393 -3.755991

C -2.645265 2.865200 -0.379129

H -2.437356 2.242071 0.494295

H -3.403397 2.372279 -0.998162

H -3.018239 3.843078 -0.055164

C 1.906326 2.317893 -1.871867

C 2.157296 1.521429 -0.738017

C 3.385069 0.867706 -0.703694

H 3.624056 0.218247 0.136732

C 4.320299 1.013489 -1.738934

C 4.037249 1.837965 -2.829403

H 4.776949 1.943226 -3.619849

C 2.816736 2.499183 -2.904441

H 2.584032 3.129258 -3.761514

C 5.615459 0.300157 -1.725891

O 6.467767 0.393612 -2.586767

O 5.756625 -0.487928 -0.645907

C 6.978657 -1.214537 -0.573664

H 7.834502 -0.531698 -0.548383

H 7.082358 -1.882701 -1.435474

C 0.158359 -2.522951 -0.901017

C 1.286157 -3.659974 -2.480356

H 2.154415 -4.116897 -2.936827

C 0.002352 -3.587627 -2.912452

H -0.464230 -3.956162 -3.814728

C 2.614719 -2.811402 -0.532859

H 2.538385 -1.893955 0.057247

H 3.437490 -2.712263 -1.247744

H 2.812833 -3.657875 0.135243

C -1.969230 -2.380963 -1.894927

C -2.178548 -1.494693 -0.716775

C -3.361366 -0.765409 -0.681840

H -3.533823 -0.075736 0.143491

C -4.337139 -0.899015 -1.678756

C -4.126512 -1.867593 -2.767721

H -4.924746 -1.983849 -3.496813

C -2.967232 -2.579016 -2.871816

H -2.812322 -3.272052 -3.697785

C -5.559557 -0.125931 -1.694842

O -6.450611 -0.239492 -2.529198

O -5.631800 0.774516 -0.679136

C -6.815028 1.556621 -0.648320

H -7.701684 0.923970 -0.525107

H -6.925977 2.138933 -1.570412

C 2.662672 0.001671 2.688537

C 2.764572 -1.275041 3.251262

H 3.489395 -1.613699 3.983753

C 1.715152 -2.011397 2.699826

C -1.632379 1.866049 2.884760

C -2.653415 1.093933 3.444131

H -3.336784 1.381350 4.236064

C -2.592260 -0.138658 2.788691

H -6.711545 2.226614 0.208251

H 6.928813 -1.793457 0.350364

C 1.292958 -3.418096 2.942863

H 2.011575 -3.935397 3.588385

H 0.308136 -3.460077 3.429622

H 1.206800 -3.983665 2.004054

C 3.500258 1.214352 2.897975

H 2.944106 2.116408 2.609584

H 3.799807 1.312453 3.948255

H 4.420266 1.186196 2.295780

C -3.415470 -1.364537 2.976700

H -4.111818 -1.521504 2.140604

H -2.780879 -2.259212 3.039986

H -4.006628 -1.295482 3.896810

C -1.198997 3.259263 3.180015

H -0.150055 3.288615 3.507361

H -1.273876 3.903471 2.291555

H -1.815370 3.700906 3.970933

**Table S23:** DFT-Optimized coordinates of **3-s** in the Triplet State in THF

Center Coordinates (Angstroms) Center Coordinates (Angstroms)

X Y Z X Y Z

Pt -0.068354 0.653776 -1.374035

Pt 0.068354 0.653629 1.374095

N 2.554466 -0.972169 -2.031939

N 0.687373 -2.069656 -1.893276

N -2.554430 -0.972448 2.031857

N -0.687272 -2.069834 1.893273

N -1.496242 2.027324 -0.613601

N -1.375897 2.159177 0.730181

N 1.375833 2.159247 -0.730006

N 1.496255 2.027249 0.613751

O -5.453977 -3.169387 -0.499248

O -5.341196 -0.922518 -0.590537

O 5.454056 -3.169348 0.499070

O 5.341174 -0.922481 0.590205

C 1.219768 -0.807124 -1.853006

C 2.849945 -2.315995 -2.168370

C 1.687266 -3.011166 -2.083273

C 3.537405 0.093533 -2.046173

C -0.690486 -2.177839 -1.689394

C -1.330756 -0.922021 -1.527504

C -2.690094 -0.923224 -1.263815

C -3.405215 -2.128700 -1.140860

C -2.736424 -3.355976 -1.314521

C -1.382254 -3.387198 -1.597501

C -4.809275 -2.159260 -0.729885

C -6.678315 -0.887297 -0.114382

C -1.219729 -0.807325 1.853004

C -2.849833 -2.316283 2.168347

C -1.687128 -3.011400 2.083191

C -3.537442 0.093187 2.045924

C 0.690585 -2.177962 1.689375

C 1.330802 -0.922120 1.527466

C 2.690133 -0.923266 1.263739

C 3.405293 -2.128717 1.140771

C 2.736564 -3.356017 1.314481

C 1.382404 -3.387294 1.597501

C 4.809327 -2.159235 0.729691

C -2.380106 2.930426 -1.068300

C -2.850005 3.664638 0.022270

C -2.180474 3.150064 1.139727

C -2.688352 3.036345 -2.520338

C -2.249387 3.535467 2.575702

C 2.180295 3.150244 -1.139496

C 2.849797 3.664795 -0.022007

C 2.380022 2.930426 1.068506

C 2.249098 3.535800 -2.575434

C 2.688346 3.036197 2.520539

C 6.678217 -0.887225 0.113835

H 3.867785 -2.657537 -2.308431

H 1.493246 -4.072887 -2.136336

H 4.517046 -0.340200 -2.265774

H 3.575774 0.594455 -1.071279

H 3.285204 0.825690 -2.820538

H -3.208379 0.021813 -1.103967

H -3.301095 -4.278805 -1.201102

H -0.863959 -4.338634 -1.715299

H -6.754914 -1.371054 0.867232

H -7.356421 -1.395706 -0.808652

H -3.867655 -2.657871 2.308424

H -1.493058 -4.073113 2.136237

H -4.517070 -0.340596 2.265481

H -3.575756 0.594041 1.070993

H -3.285358 0.825420 2.820258

H 3.208370 0.021790 1.103856

H 3.301279 -4.278822 1.201076

H 0.864162 -4.338750 1.715360

H -3.571657 4.474177 0.001610

H -3.012797 2.070284 -2.931927

H -1.802918 3.350475 -3.092130

H -3.483353 3.768100 -2.700564

H -1.563209 2.911790 3.165782

H -3.260840 3.414282 2.986915

H -1.963536 4.584392 2.726309

H 3.571367 4.474406 -0.001288

H 1.563133 2.911947 -3.165580

H 3.260594 3.414996 -2.986648

H 1.962889 4.584642 -2.725946

H 3.013017 2.070138 2.931955

H 1.802890 3.350060 3.092445

H 3.483211 3.768086 2.700818

H 6.754694 -1.371134 -0.867713

H 7.356472 -1.395470 0.808081

H -6.942734 0.169353 -0.034446

H 6.942534 0.169434 0.033692

**Table S24:** DFT-Optimized coordinates of **3-TS** in the Triplet State in THF

Center Coordinates (Angstroms) Center Coordinates (Angstroms)

X Y Z X Y Z

Pt -0.390312 0.644933 -1.436115

Pt 0.419111 0.563410 1.452808

N 1.943960 -1.099298 -2.611747

N -0.008686 -2.000067 -2.476673

N -1.919114 -1.241079 2.528155

N 0.067749 -2.122425 2.411714

N -1.569071 1.942899 -0.250764

N -1.202680 1.953154 1.055282

N 1.180008 2.045448 -0.957818

N 1.563290 1.964042 0.340976

O -6.051987 -2.830140 -0.534754

O -5.644279 -0.672148 -0.059556

O 6.081956 -2.787333 0.453811

O 5.617224 -0.654745 -0.142337

C 0.661426 -0.819822 -2.295954

C 2.074451 -2.432520 -2.980192

C 0.848715 -3.000778 -2.893502

C 3.067956 -0.179676 -2.547842

C -1.365514 -2.024600 -2.079502

C -1.815319 -0.781226 -1.594155

C -3.121517 -0.742100 -1.114938

C -3.934599 -1.885359 -1.116757

C -3.448579 -3.092381 -1.623723

C -2.150385 -3.169588 -2.113798

C -5.304893 -1.872101 -0.560685

C -0.627883 -0.931991 2.243432

C -2.028818 -2.576196 2.869418

C -0.795577 -3.137441 2.801645

C -3.037555 -0.316149 2.471867

C 1.399037 -2.116070 2.088337

C 1.835086 -0.805377 1.535985

C 3.116635 -0.747431 0.995606

C 3.973458 -1.856054 1.014725

C 3.520727 -3.105445 1.643418

C 2.263689 -3.227367 2.159320

C 5.301787 -1.841728 0.441599

C -2.568762 2.822518 -0.430586

C -2.854805 3.419242 0.800156

C -1.961032 2.846655 1.709962

C 1.836764 3.062227 -1.537421

C 2.680905 3.644097 -0.587230

C 2.467573 2.925153 0.591677

H 3.030592 -2.845545 -3.272368

H 0.523540 -4.012866 -3.088810

H 2.745497 0.733044 -2.040822

H 3.418956 0.059672 -3.557809

H 3.882075 -0.635521 -1.973003

H -3.515387 0.187163 -0.706166

H -4.094328 -3.967500 -1.616087

H -1.760501 -4.112575 -2.494206

H -2.983793 -3.013392 3.129902

H -0.467841 -4.151936 2.976672

H -3.967485 -0.890407 2.509159

H -3.001363 0.250262 1.536039

H -3.004836 0.380576 3.317303

H 3.458293 0.183510 0.544236

H 4.222739 -3.935188 1.673028

H 1.933871 -4.166052 2.602659

H -3.597824 4.183906 0.999412

H 3.347474 4.488183 -0.728751

C -6.945613 -0.591910 0.512427

H -7.056292 0.436063 0.862040

H -7.712245 -0.824434 -0.234231

H -7.042531 -1.290840 1.350541

C 6.911556 -0.591422 -0.721131

H 7.007339 0.414017 -1.136788

H 7.687635 -0.762314 0.033602

H 7.024328 -1.339378 -1.514720

C 3.045891 3.107773 1.951067

H 2.259118 3.318462 2.689803

H 3.574415 2.206976 2.292596

H 3.755324 3.942698 1.962404

C 1.602339 3.408147 -2.966410

H 1.783460 2.547516 -3.626865

H 0.562671 3.724537 -3.132785

H 2.260059 4.224649 -3.284480

C -1.761083 3.102035 3.162783

H -1.081390 2.350786 3.588207

H -1.316382 4.090303 3.342170

H -2.707835 3.067195 3.717721

C -3.184113 3.027691 -1.770597

H -2.444776 2.849859 -2.563393

H -4.024227 2.340184 -1.946971

H -3.567848 4.048983 -1.876456

**Table S25:** DFT-Optimized coordinates of **4-f** in the Triplet State in THF

Center Coordinates (Angstroms) Center Coordinates (Angstroms)

X Y Z X Y Z

Pt 0.440956 1.297054 0.164122

Pt -0.422706 -1.303355 0.007902

N -1.904948 2.396834 1.980246

N -0.065746 1.770754 2.952059

N 1.930031 -2.598607 1.683710

N 0.078113 -2.124069 2.713588

N 1.610030 0.273491 -1.292062

N 1.177662 -0.992283 -1.467777

N -1.166317 1.168356 -1.345981

N -1.588891 -0.112321 -1.312261

O 5.775773 -0.781764 3.580473

O 5.555354 -0.503404 1.356380

O -5.744674 0.378949 3.629106

O -5.534176 0.329254 1.386983

C -0.641537 1.964210 1.720378

C -2.109687 2.460871 3.345679

C -0.966114 2.071725 3.962810

C -2.927682 2.680720 0.992381

C 1.236810 1.270704 2.969527

C 1.765141 1.054100 1.671780

C 3.051634 0.555626 1.569335

C 3.796036 0.234290 2.718970

C 3.234757 0.445439 3.993242

C 1.961771 0.972984 4.125389

C 5.119799 -0.385861 2.630461

C 6.811000 -1.149711 1.196129

C 0.666869 -2.141626 1.474403

C 2.123748 -2.846968 3.029985

C 0.972329 -2.552019 3.683188

C 2.956434 -2.760020 0.671581

C -1.231557 -1.644041 2.784499

C -1.760785 -1.284559 1.518897

C -3.044379 -0.768782 1.478919

C -3.785796 -0.577218 2.658152

C -3.225234 -0.934504 3.899110

C -1.953342 -1.477442 3.967800

C -5.099368 0.071051 2.640311

C 2.675364 0.495739 -2.085646

C 2.936607 -0.674223 -2.801042

C 1.955550 -1.589638 -2.394232

C -1.971769 1.863636 -2.176366

C -2.959076 0.995058 -2.663499

C -2.674308 -0.248037 -2.097766

C -6.769325 1.026872 1.298823

H -3.061208 2.773782 3.756246

H -0.722271 1.986084 5.012111

H -3.817970 3.046428 1.511477

H -3.185657 1.770693 0.436807

H -2.582286 3.448561 0.292077

H 3.487278 0.375772 0.587548

H 3.816546 0.177979 4.872678

H 1.531609 1.135156 5.113055

H 6.771805 -2.175857 1.579865

H 7.601993 -0.604998 1.723150

H 3.075025 -3.203545 3.403774

H 0.720127 -2.608409 4.732498

H 2.666411 -3.526681 -0.055580

H 3.883984 -3.065588 1.163421

H 3.124383 -1.811391 0.149043

H -3.477885 -0.467338 0.526829

H -3.803015 -0.763079 4.804843

H -1.519535 -1.746201 4.930292

H 3.721830 -0.823067 -3.533946

H -3.763614 1.221062 -3.354327

H -6.697005 2.007200 1.784337

H -7.576250 0.456313 1.771442

H -6.970068 1.150875 0.232720

H 7.010788 -1.158381 0.122786

C -1.762896 3.278892 -2.498496

C -0.964992 4.120382 -1.709993

C -2.371618 3.821429 -3.637964

C -0.778049 5.454666 -2.055148

H -0.486036 3.731026 -0.810173

C -2.189162 5.155990 -3.978734

H -2.982623 3.182916 -4.274653

C -1.389133 5.979785 -3.190478

H -0.153539 6.087294 -1.425938

H -2.670437 5.552886 -4.871012

H -1.243038 7.024239 -3.459636

C -3.354870 -1.538754 -2.229856

C -4.748140 -1.612987 -2.138907

C -2.619004 -2.716304 -2.415902

C -5.393255 -2.843290 -2.216956

H -5.319688 -0.698028 -1.978218

C -3.266030 -3.945283 -2.489680

H -1.534098 -2.663550 -2.521326

C -4.654115 -4.012190 -2.387079

H -6.477938 -2.890543 -2.136455

H -2.682060 -4.852793 -2.638421

H -5.160252 -4.974272 -2.444499

C 1.719681 -2.955637 -2.873800

C 0.981420 -3.894786 -2.138788

C 2.236106 -3.345915 -4.116397

C 0.760945 -5.174843 -2.635725

H 0.576115 -3.629154 -1.161312

C 2.020177 -4.627110 -4.609418

H 2.798549 -2.627314 -4.711323

C 1.278647 -5.547952 -3.873108

H 0.184724 -5.885682 -2.045140

H 2.428301 -4.905114 -5.579685

H 1.106128 -6.550189 -4.261039

C 3.353508 1.794386 -2.095674

C 4.749329 1.864935 -2.061296

C 2.610658 2.982142 -2.122831

C 5.390321 3.099729 -2.038583

H 5.328019 0.940989 -2.032384

C 3.253403 4.215018 -2.096941

H 1.522962 2.933288 -2.193374

C 4.644691 4.276878 -2.051028

H 6.477626 3.143147 -2.005410

H 2.663699 5.130501 -2.125739

H 5.148256 5.241798 -2.031922

**2.4.-Photophysical Study**

**Table S26.** Absorption data in solution of 2-MeTHF 10^-3^ M for **1**-**3** and 10^-5^ M for **4** at 298 K.

| **Comp.** | λ_abs_ /nm (10^3^ ε M^-1^ cm^-1^) |
| --- | --- |
| **1** | 245 (45.82), 309 (11.28), 335 (11.84), 354sh (9.03) tail to 425 |
| **2** | 241 (48.70), 249 (51.37), 310 (12.16), 338 (13.97), 359sh (9.41) tail to 400 |
| **3** | 244 (45.27), 249 (41.81), 308 (8.85), 348 (9.52), 362 (9.28) tail to 425 |
| **4** | 475 (45.29), 331 (8.58), 377 (7.80) tail to 450 |


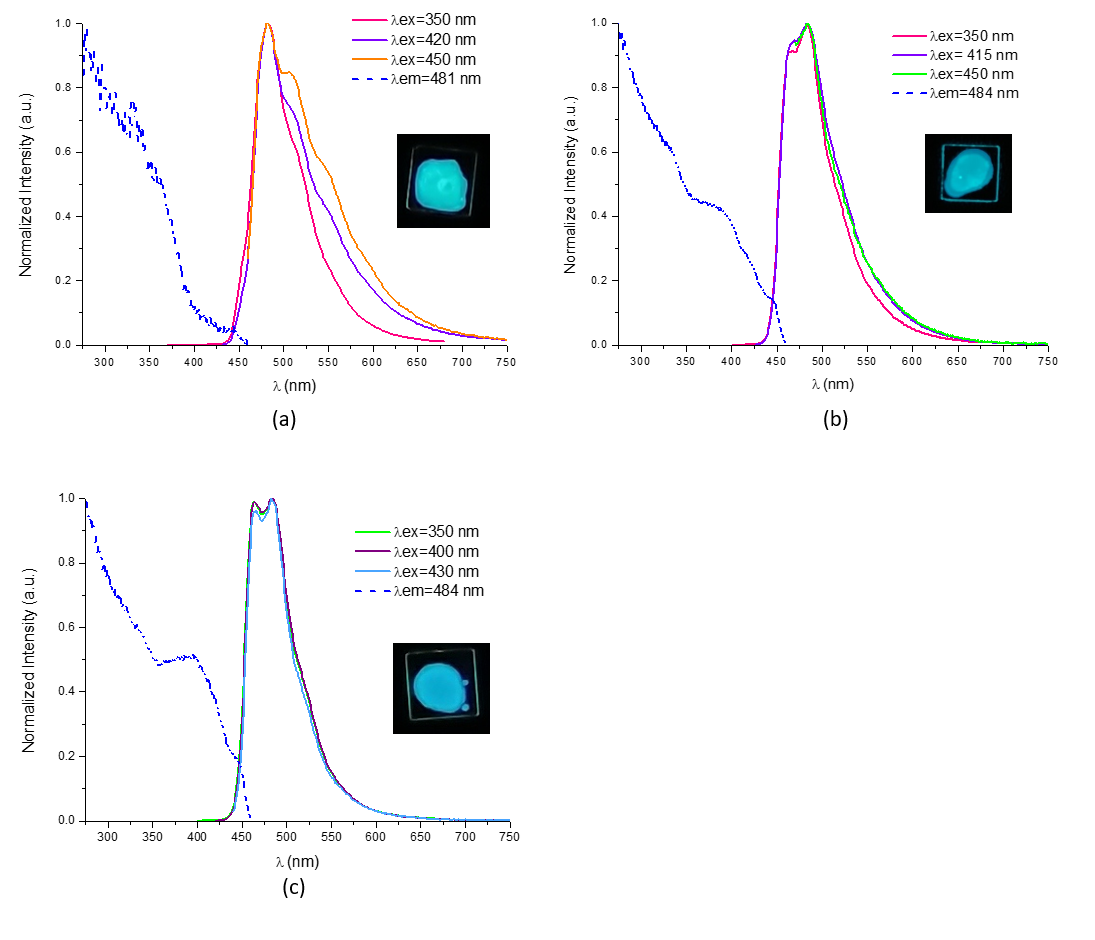


Figure S12.Normalized emission and excitation spectra of complex **1** (a), **2** (b), **3** (c) in 5% wt films of PMMA at room temperature in the air. Pictures were taken under UV light (365 nm).

**Table S27.** Quantum yield data measured in 5% doped PMMA films for **1**-**4** at 298 K in the air.

| Compound | λ_exc_ (nm) | ***φ (%)*** |
| --- | --- | --- |
| **1** | 350 | 72.1 |
|  | 380 | 28.9 |
|  | 390 | 19.9 |
|  | 420 | 12.4 |
|  | 450 | 9.3 |
| **2** | 370 | 83.0 |
|  | 390 | 54.3 |
|  | 420 | 46.1 |
|  | 450 | 41.6 |
| **3** | 380 | 79.0 |
|  | 390 | 53.4 |
|  | 400 | 48.2 |
|  | 430 | 40.5 |
| **4** | 380 | 85.9 |
|  | 390 | 69.0 |
|  | 430 | 59.2 |
|  | 450 | 55.4 |

**
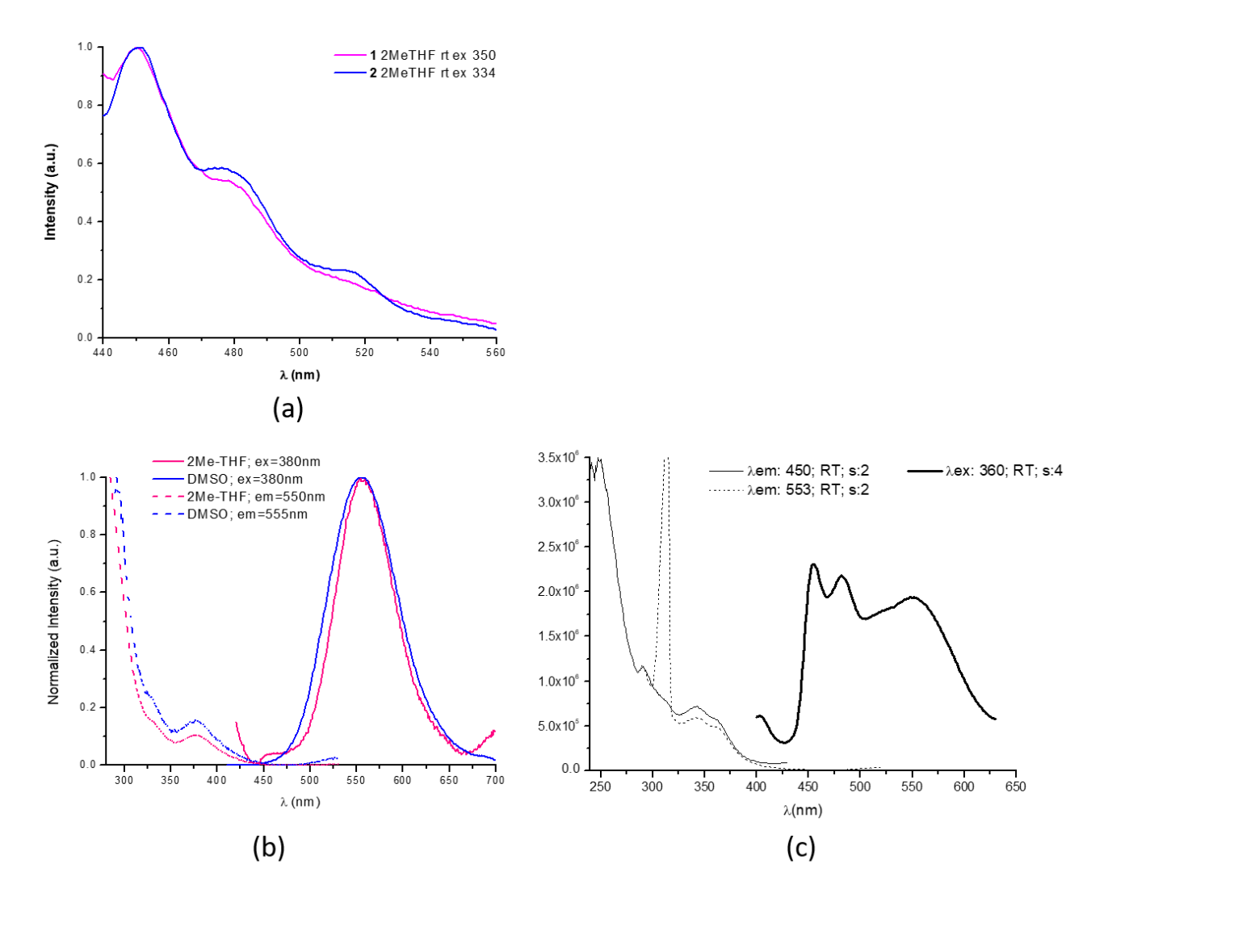
**

**Figure S13**: Normalized emission and excitation spectra at r.t. of complexes **1** and **2** in THF (a) **4** in DMSO or 2-MeTHF (10^-5^M) in the air (b) and of complex **3** in 2-MeTHF (10^-5^M) under Ar (c).

**
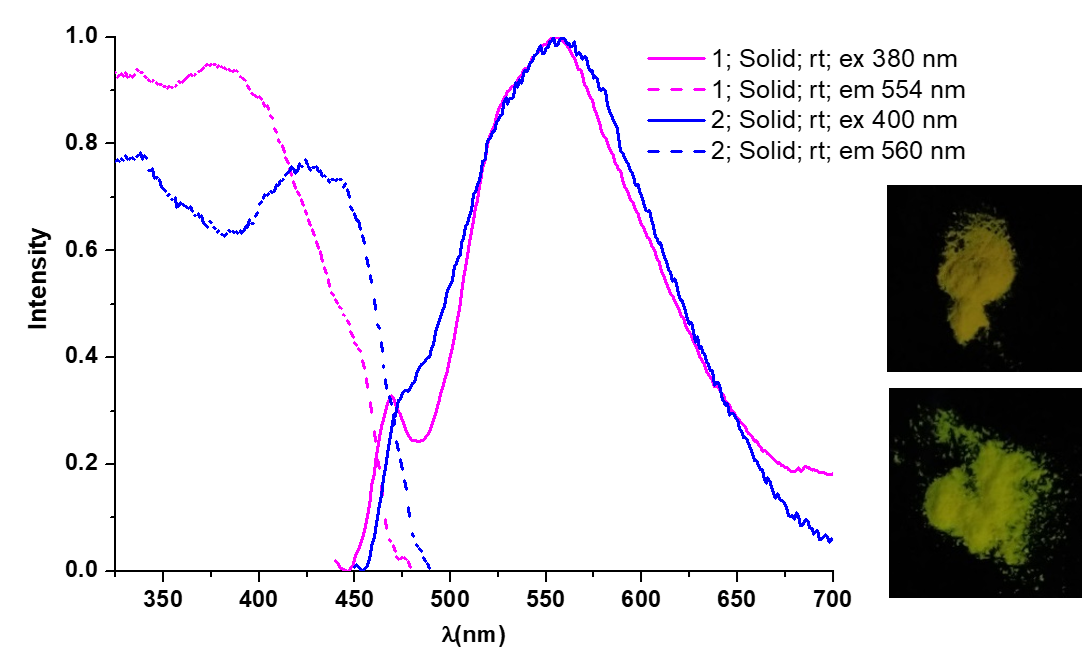
**

**Figure S14**: Normalized emission and excitation spectra of complexes **1** and **2** in solid state at room temperature in the air. Pictures were taken under 365-UV light for **1** (top) and **2** (bottom)

**Figure S15**: Normalized emission and excitation spectra of complex **2** in solid state at room temperature in the air before (purple) and after (blue) grinding.


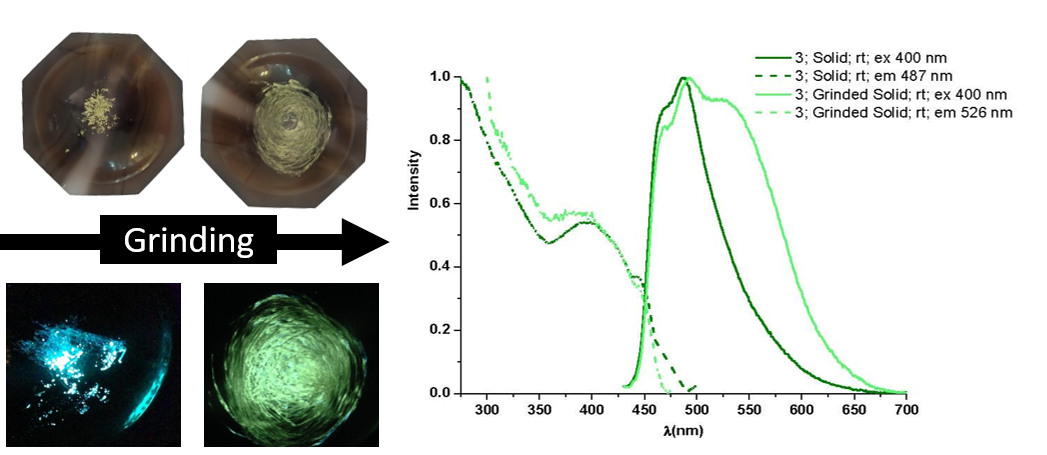


**Figure S16**: Normalized emission and excitation spectra of complex **3** in solid state at room temperature in the air before and after grinding; pictures of solid were taken before and after grinding (up) and under 365 nm-UV light (bottom).

**Figure S17**: Normalized emission spectra of complex **4** in 5% wt and 40% wt PMMA film in the air.

**Figure S18**: Normalized emission and excitation spectra at room temperature and at 77k of the grinded solid, **4-g** in the air.


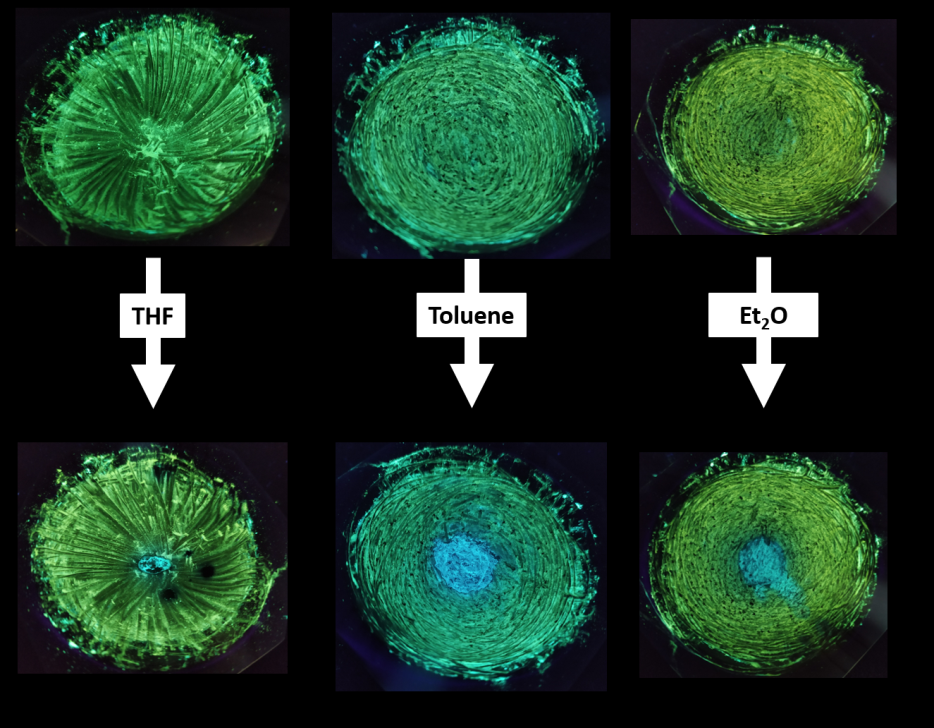


**Figure S19.** Photographic images of mechanical grinding samples of **3** in response to solvent treatment taken under 365 nm-UV light

**3.-REFERENCES.**

1. CrysAlis RED, CCD camera data reduction program. *Rigaku Oxford Diffraction* **2019,** *Oxford Diffraction: Oxford, UK*.

2. Sheldrick, G. M., SHELXT - Integrated Space-Group and Crystal-Structure Determination. *Acta Crystallogr., Sect. A: Found. Adv.* **2015,** *71*, 3-8.

3. Frisch, M. J.; Trucks, G. W.; Schlegel, H. B.; Scuseria, G. E.; Robb, M. A.; Cheeseman, J. R.; Scalmani, G.; Barone, V.; Petersson, G. A.; Nakatsuji, H.; Li, X.; Caricato, M.; Marenich, A. V.; Bloino, J.; Janesko, B. G.; Gomperts, R.; Mennucci, B.; Hratchian, H. P.; Ortiz, J. V.; Izmaylov, A. F.; Sonnenberg, J. L.; Williams; Ding, F.; Lipparini, F.; Egidi, F.; Goings, J.; Peng, B.; Petrone, A.; Henderson, T.; Ranasinghe, D.; Zakrzewski, V. G.; Gao, J.; Rega, N.; Zheng, G.; Liang, W.; Hada, M.; Ehara, M.; Toyota, K.; Fukuda, R.; Hasegawa, J.; Ishida, M.; Nakajima, T.; Honda, Y.; Kitao, O.; Nakai, H.; Vreven, T.; Throssell, K.; Montgomery Jr., J. A.; Peralta, J. E.; Ogliaro, F.; Bearpark, M. J.; Heyd, J. J.; Brothers, E. N.; Kudin, K. N.; Staroverov, V. N.; Keith, T. A.; Kobayashi, R.; Normand, J.; Raghavachari, K.; Rendell, A. P.; Burant, J. C.; Iyengar, S. S.; Tomasi, J.; Cossi, M.; Millam, J. M.; Klene, M.; Adamo, C.; Cammi, R.; Ochterski, J. W.; Martin, R. L.; Morokuma, K.; Farkas, O.; Foresman, J. B.; Fox, D. J. *Gaussian 16 Rev. C.01*, Wallingford, CT, 2016.

4. Zhao, Y.; Truhlar, D. G., The M06 Suite of Density Functionals for Main Group Thermochemistry, Thermochemical Kinetics, Noncovalent Interactions, Excited States, and Transition Elements: two New functionals and Systematic Testing of four M06-Class Functionals and 12 other functionals. *Theor. Chem. Acc.* **2008,** *120*, 215-241.

5. Wang, Y.; Verma, P.; Jin, X.; Truhlar, D. G.; He, X., Revised M06 Density Functional for Main-group and Transition-Metal Chemistry. *Proc. Natl. Acad. Sci. U.S.A.* **2018,** *115*, 10257.

6. Grimme, S.; Antony, J.; Ehrlich, S.; Krieg, H., A Consistent and Accurate ab initio Parametrization of Density Functional Dispersion Correction (DFT-D) for the 94 Elements H-Pu. *J. Chem. Phys.* **2010,** *132*, 154104.

7. Andrae, D.; Häußermann, U.; Dolg, M.; Stoll, H.; Preuß, H., Energy-Adjusted ab initio Pseudopotentials for the Second and Third Row Transition Elements. *Theor. Chim. Acta* **1990,** *77*, 123-141.

8. Ditchfield, R.; Hehre, W. J.; Pople, J. A., Self‐Consistent Molecular‐Orbital Methods. IX. An Extended Gaussian‐Type Basis for Molecular‐Orbital Studies of Organic Molecules. *J. Chem. Phys.* **1971,** *54,* 724-728.

9. Hariharan, P. C.; Pople, J. A., The Influence of Polarization Functions on Molecular Orbital Hydrogenation Energies. *Theor. Chim. Acta* **1973,** *28*, 213-222.

10. Tomasi, J.; Mennucci, B.; Cammi, R., Quantum Mechanical Continuum Solvation Models. *Chem. Rev.* **2005,** *105*, 2999-3094.

11. Arnal. L.; Fuertes, S.; Martín, A.; Baya, M.; Sicilia, V., A Cyclometalated N-Heterocyclic Carbene: The Wings of the First Pt_2_(II,II) Butterfly Oxidized by CHI_3_. *Chem. Eur. J.* **2018,** *24*, 18743-18748.

12. Fuertes, S.; Chueca, A. J.; Martín, A.; Sicilia, V., New NHC cycloplatinated compounds. Significance of the cyclometalated group on the electronic and emitting properties of biscyanide compounds. *J. Organomet. Chem.* **2019,** *889*, 53-61.

13. Sicilia, V.; Arnal, L.; Fuertes, S.; Martín, A.; Baya, M., Metal−Metal Cooperation in the Oxidation of a Flapping Platinum Butterfly by Haloforms: Experimental and Theoretical Evidence. *Inorg. Chem.* **2020,** *59*, 12586-12594.

14. Arnal, L.; Fuertes, S.; Martín, A.; Sicilia, V., *The Use of Cyclometalated NHCs and Pyrazoles for the Development of Fully Efficient Blue Pt^II^ Emitters and Pt/Ag Cluster. Chem. Eur. J.* **2018,** *24*, 9377-9384.

15. Ma, B.; Li, J.; Djurovich, P. I.; Yousufuddin, M.; Bau, R.; Thompson, M. E., Synthetic Control of Pt···Pt Separation and Photophysics of Binuclear Platinum Complexes. *J. Am. Chem. Soc.* **2005,** *127*, 28-29.

16. Umakoshi, K.; Kimura, K.; Kim, Y. H.; Tsukimoto, Y.; Arikawa, Y.; Onishi, M.; Ishizaka, S.; Kitamura, N., Pyrazolato- and 3,5-Dimethylpyrazolato-Bridged Dinuclear Platinum(II), Palladium(II), and Their Mixed-Metal Complexes of 2,2′-Bipyrimidine. Syntheses, Structures, and Luminescent Properties. *Bull. Chem. Soc. Jpn.* **2010,** *83*, 1504–1510

17. Ghavale, N.; Wadawale, A.; Dey, S.; Jain, V. K., Synthesis, structures and spectroscopic properties of platinum complexes containing orthometalated 2-phenylpyridine. *J. Organomet. Chem.* **2010,** *695*, 1237-1245.

18. Sicilia, V.; Forniés, J.; Casas, J. M.; Martín, A.; López, J. A.; Larraz, C.; Borja, P.; Ovejero, C.; Tordera, D.; Bolink, H., Highly Luminescent Half-Lantern Cyclometalated Platinum(II) Complex: Synthesis, Structure, Luminescence Studies, and Reactivity. *Inorg. Chem.* **2012,** *51*, 3427-3435.

19. Sicilia, V.; Borja, P.; Casas, J. M.; Fuertes, S.; Martín, A., Selective Synthesis of new half-Lantern Benzoquinolate Platinum Complexes. DFT and Photophysical Studies on the Platinum (II,II) Derivative. *J. Organomet. Chem.* **2013,** *731*, 10-17.

20. Sicilia, V.; Baya, M.; Borja, P.; Martín, A., Oxidation of Half-Lantern Pt_2_(II,II) Compounds by Halocarbons. Evidence of Dioxygen Insertion into a Pt(III)–CH_3_ Bond. *Inorg. Chem.* **2015,** *54*, 7316-7324.

21. Forniés, J.; Sicilia, V.; Borja, P.; Casas, J. M.; Díez, A.; Lalinde, E.; Larraz, C.; Martín, A.; Moreno, M. T., Luminescent benzoquinolate-Isocyanide platinum(II) Complexes: Effect of Pt···Pt and π-π Interactions on their Photophysical properties. *Chem. Asian J.* **2012,** *7*, 2813-2823.

22. Doerrer, L. H., Steric and Electronic Effects in Metallophilic Double Salts. *Dalton Trans.* **2010,** *39*, 3543-3553.
